# Distribution and bioactivities of tirucallane-type triterpenoids from Meliaceae family

**DOI:** 10.1007/s13659-026-00631-1

**Published:** 2026-05-28

**Authors:** Fajar Fauzi Abdullah, Sabrina Aisyah Putri, Noor Aishah Najihah, Nurlelasari, Wahyu Safriansyah, Khalijah Awang, Unang Supratman

**Affiliations:** 1 Department of Chemistry, Faculty of Mathematics and Natural Sciences, Universitas Padjadjaran, Jatinangor, Sumedang, 45363 Indonesia; 2 Central Laboratory, Universitas Padjadjaran, Jatinangor, Sumedang, 45363 Indonesia; 3 Eijkman Research Center for Molecular Biology, National Research and Innovation Agency, Jalan Raya Bogor Km. 46, Cibinong, Bogor, 16911 Indonesia; 4 Department of Chemistry, Faculty of Mathematics and Natural Sciences, Universitas Garut, Garut, 44151 Indonesia; 5 Department of Chemistry, Faculty of Science, University of Malaya, 50603 Kuala Lumpur, Malaysia

**Keywords:** Tirucallane, Meliaceae, Triterpenoids, Biological activities, Secondary metabolites

## Abstract

Tirucallane-type triterpenoids are one of the most structurally diverse classes of secondary metabolites in Meliaceae family, which is predominantly distributed across tropical and subtropical regions. A total of 373 tirucallane derivatives have been reported between 1967 and 2025, from 48 species belonging to 17 genera. These compounds were isolated from different plant parts, including bark, stem bark, roots, leaves, fruits, twigs, seeds, and aerial tissues. Therefore, this review aims to integrate current reports on tirucallane-type triterpenoids from 17 Meliaceae genera, emphasizing structural complexity, taxonomic occurrence, and pharmacological relevance to guide future chemotaxonomic and drug discovery efforts. Literature collection was performed through Google Scholar, Reaxys, PubMed, and SciFinder. The results showed that based on the structural characteristics, tirucallane derivatives were categorized into intact, seco, heteroatom-substituted, degraded, and highly rearranged, with the intact subgroup being the most abundant. Furthermore, tirucallane derivatives have a broad spectrum of biological properties, including cytotoxic, antimicrobial, antiplasmodial, antidiabetic, anti-inflammatory, immunomodulatory, FXR agonistic, and insecticidal effects, indicating significant potential therapeutic value. Among the reported compounds, laxiracemosin E (**361**) showed the strongest anticancer potential with IC_50_ = 1.5 µM, while aphanamgrandin C (**372**), aphanamgrandin D (**373**), and dubione B (**227**) demonstrated potent antibacterial activity (MIC = 1.57–3.13 µg/mL). Hispidol A (**2**) indicated remarkable antiplasmodial activity (IC_50_ = 2.45 µM), while meliasenin G (**64**), phellochin (**66**), and methyl angolensate (**343**) were identified as promising α-glucosidase inhibitors (IC_50_ = 6.1 µM). In conclusion, tirucallane triterpenoids from Meliaceae offer an outstanding structural platform for future drug discovery and bioactivity optimization.

**Graphical Abstract:**

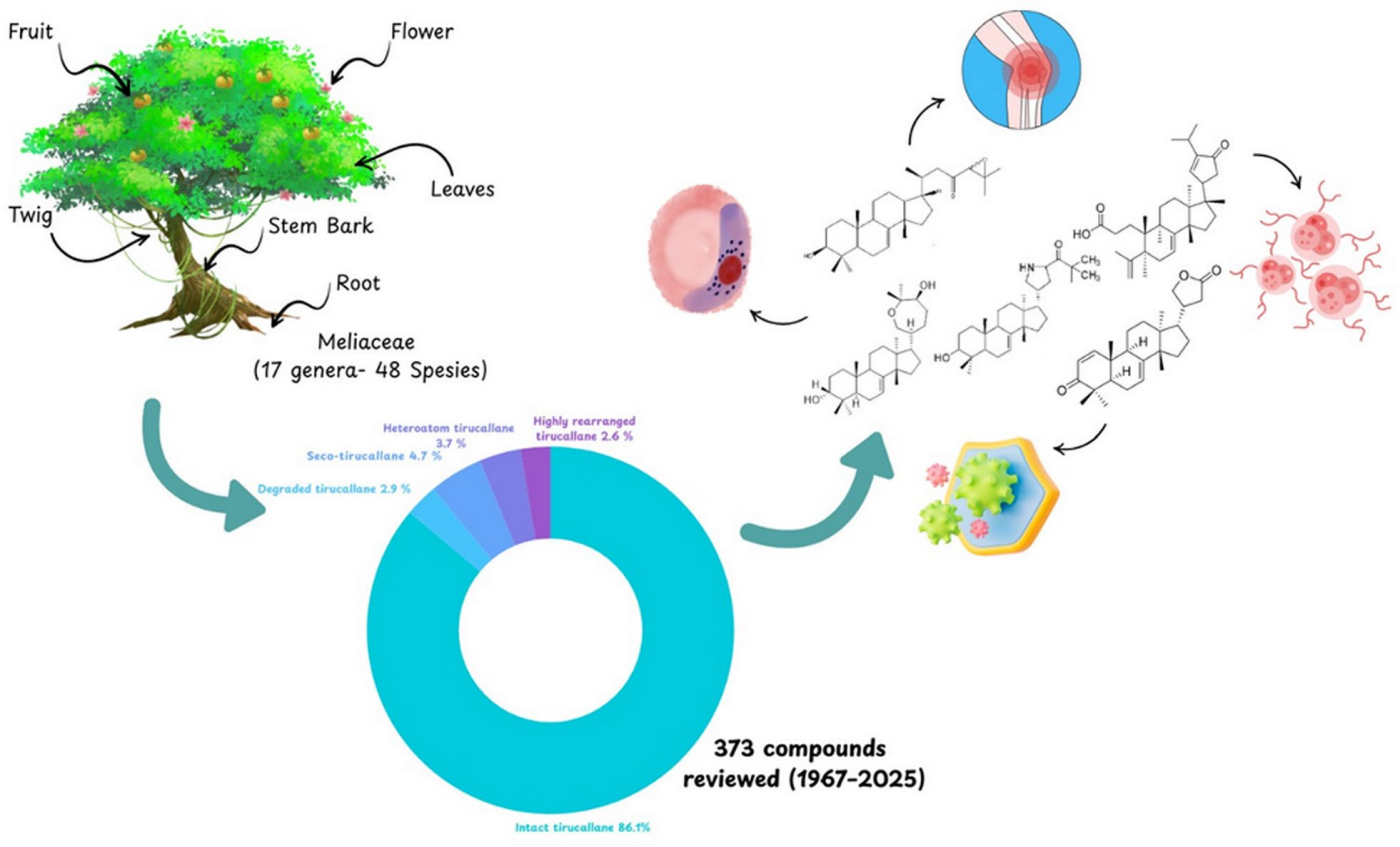

## Introduction

Triterpenoids are one of the most structurally diverse classes of plant-derived secondary metabolites characterized by a C30 carbon framework, originating from a head-to-tail assembly of six isoprene units through the mevalonate pathway. These compounds are not only widely distributed across many plant species but also have an extensive range of structural types and biological activities [1]. The extensive range of biological activities, such as cytotoxic, anti-inflammatory, hepatoprotective, antimicrobial, and antiparasitic effects make triterpenoids an important reservoir of bioactive natural products for drug discovery [2, 3].

In the broad class, tirucallane-type triterpenoids represent a distinctive subgroup defined by a sterol-like tetracyclic carbon framework, typically featuring $\Delta$ ^7^,^8^ or $\Delta$ ^24^,^25^, double bonds, often accompanied by diverse oxidative substituents, including hydroxyl, carbonyl, and carboxyl groups. These oxidative decorations contribute significantly to the chemical diversity and serve as crucial intermediates in the formation of limonoids, which are typical constituents of Meliaceae family [4].

The Meliaceae family comprises woody plants consisting of approximately 740 species distributed among 58 genera, predominantly found in tropical regions across Africa–Madagascar, Malaya–Indo, and Australia–Asia. Phytochemical studies have shown that Meliaceae produces a wide range of secondary metabolites, including phenolics, namely flavonoids, lignans, and chromones, as well as terpenoids in the form of sesquiterpenoids, diterpenoids, triterpenoids, and limonoids [5–8]. Representative reports include bioactive limonoids with reported anti-inflammatory relevance and lignan glycosides from the bark of *Aglaia eximia* [9, 10]. Additionally, representative studies also include the discovery of new cytotoxic limonoid from the stem bark of *Chisocheton pentandrus* as well as sesquiterpenoids and sesquiterpenoid dimers from the stem bark of *Dysoxylum parasiticum* [11–13].

Tirucallane-type triterpenoids have been reported across multiple Meliaceae genera, characterized by substantial structural variability, particularly in oxygenation patterns and side-chain modifications, which have been associated with diverse biological activities [14–17]. Despite numerous studies across individual species, a detailed genus-level synthesis of tirucallane distribution in Meliaceae remains scarce. Therefore, this review aims to integrate current reports on tirucallane-type triterpenoids from 17 Meliaceae genera, emphasizing structural complexity, taxonomic occurrence, and pharmacological relevance to guide future chemotaxonomic and drug discovery efforts.

## Method and materials

Relevant studies were retrieved from major scientific databases, including Google Scholar, SciFinder, PubMed, Web of Science, and Reaxys, using keyword combinations, namely “tirucallane”, “tirucallane-type triterpenoid”, “Meliaceae triterpenoid”, “limonoid precursor”, “cyclic side-chain triterpenoid”, and “*Seco-*tirucallane”. The screening process included duplicate removal, title and abstract assessment, as well as full-text evaluation. Only peer-reviewed studies reporting purified tirucallane-type triterpenoids from taxonomically verified Meliaceae species were included, with structural confirmation based on NMR, MS, IR, UV, ECD, or X-ray data. Studies published between 1967 and 2025 were incorporated, covering all Meliaceae genera reported as natural sources of tirucallane-type triterpenoids. Extracted variables included compound name, plant species, anatomical part used, extraction solvent, and bibliographic reference. All compounds were subsequently organized and classified according to the integrity of tirucallane core and the structural architecture of the side chain to develop a comprehensive chemotype-based categorization.

## Phytochemistry

### Overview of tirucallane-type from Meliaceae plant

A comprehensive review of phytochemical discoveries from 1967 to 2025 produced a total of 373 tirucallane-type triterpenoids isolated from different anatomical parts of Meliaceae species, including the bark, stem bark, wood, roots, leaves, fruits, twigs, seeds, and aerial tissues. These compounds originate from 17 genera and 48 species of Meliaceae plants reported as natural sources of tirucallane-type metabolites. The genus *Aglaia* contributed three species, namely, *A. andamanica, A. duperreana*, and *A. leucophylla*, while *Amoora* was represented by *A. dasyclada* and *A. tetrapetala*. The genus *Aphanamixis* included two frequently investigated species, *A. grandifolia* and *A. polystachya*, and *Azadirachta* was represented exclusively by *A. indica*. *Cedrela* contributed four species, including *C. fissilis, C. glaziovii, C. odorata,* and *C. sinensis*, while *Chisocheton* showed the second-highest species richness, represented by *C. cumingianus ssp. balansae, C. lasiocarpus, C. paniculatus, C. patens*, and *C. pentandrus*. In contrast, *Dysoxylum* constituted the most diverse genus, comprising eight species*,* namely *D. binectariferum, D. gaudichaudianum, D. hainanense, D. laxiracemosum, D. lenticellatum, D. lukii, D. macranthum*, and *D. variabile*. Additional genera included *Chukrasia (C. tabularis*), *Entandrophragma* (*E. angolense, E. congoënse*), *Guarea* (*G. guidonia*), and *Melia* (*M. azedarach, M. toosendan, M. volkensii*). *Munronia* was represented by *M. delavayi*, and *Swietenia* by *S. humilis*. The genus *Toona* showed significant taxonomic breadth with *T. ciliata* var. *ciliata,* var. *henryi,* var. *pubescens, T. ciliatawas*, and *T. sinensis*, while four species of *Trichilia,* including *T. connarozdes, T. hispida, T. maynasiana*, and *T. hispida* from a separate report, were also included. The remaining genera, *Walsura* (*W. piscidia, W. trichostemon*) and *Xylocarpus* (*X. granatum, X. moluccensis*), are known for abundant limonoid and tirucallane profiles. This broad distribution underscores the extensive chemical diversity of tirucallane derivatives in Meliaceae family.

**Fig. 1:**
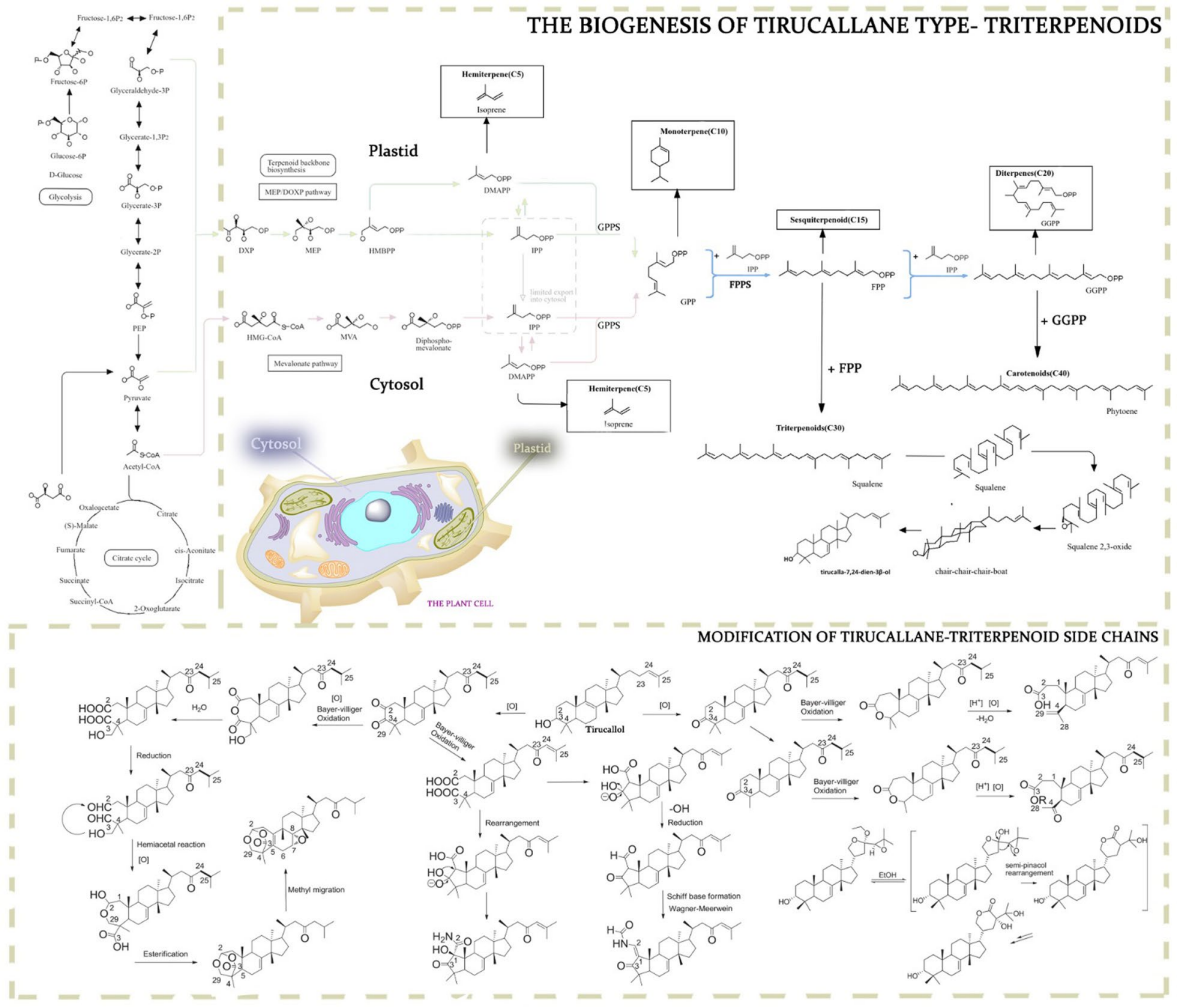
The biogenesis relationship between tirucallane-types

The proposed biogenetic pathway of tirucallane-type triterpenoids is illustrated in Fig. 1. Primary metabolism provides the fundamental precursors that enter into isoprenoid biosynthesis. During glycolysis, glucose-derived intermediates are converted through key enzymatic steps, including isomerization and phosphorylation reactions that direct carbon flux toward pyruvate formation [17]. Pyruvate can then be transformed into acetyl-CoA, which supports the formation of isoprenoid building blocks. Terpenoid biosynthesis proceeds through the generation of isopentenyl pyrophosphate (IPP) and dimethylallyl pyrophosphate (DMAPP) via the mevalonate (MVA) and methylerythritol phosphate (MEP) pathways [18]. Sequential prenyltransferase-catalyzed condensations generate prenyl diphosphates, including geranyl diphosphate (GPP) formed by GPPS and ultimately farnesyl pyrophosphate (FPP), a central intermediate that provides the immediate precursor pool for triterpenoid biosynthesis. The head-to-head coupling of two FPP units yields squalene, which is subsequently oxidized to 2,3-oxidosqualene [19]. Cyclization of 2,3-oxidosqualene by oxidosqualene cyclases, following enzyme-directed substrate folding, produces tetracyclic triterpenoid frameworks, including tirucallane core represented by tirucallol (tirucalla-7,24-dien-3β-ol).

Tirucallol serves as the crucial precursor for structurally diverse tirucallane-type triterpenoids. A series of post-cyclization modifications, including reduction, oxidation, methyl migration, skeletal rearrangement, and esterification, drives the diversification. The reaction starts with tirucallol, which serves as the base structure for tirucallane-type compounds. Structural modification of tirucallane skeleton occurs mainly through oxidation, Baeyer–Villiger oxidation, ring scission, rearrangement, and heteroatom incorporation, which collectively generate intact, degraded, *Seco-*, heteroatom-containing, and highly rearranged tirucallane derivatives. Early oxidative tailoring produces oxidized intermediates that are amenable to further modification [20]. Oxidosqualene cyclases then catalyze tetracyclic scaffolds, including tirucalla-7,24-dien-3$\upbeta$-ol, which serve as an intact tirucallane core before ring A modification. For instance, in *Aphanamixis grandifolia*, tirucallane triterpenoids such as 3$\upbeta$,25-dihydroxy-tirucalla-7,23-diene and (23*Z*)-25-hydroxy-tirucalla-7,23-diene-3-one retain the full C30 skeleton and four fused rings. The compounds experience oxidation, epoxidation, or olefin migration at C-3, C-7, and the C-23–C-25 side chain. These intact tirucallanes represent initial structures in biosynthetic pathways before Baeyer–Villiger oxidation at ring A.

*Seco-*tirucallanes are formed when oxidative cleavage opens ring A of tirucallane structure, a process well-supported in triterpenoid biosynthesis. In general, triterpenoids with a 3-oxo precursor are subjected to Baeyer–Villiger oxidation, which leads to the formation of 7-membered lactones or anhydrides. Subsequent hydrolysis or dehydration leads to the formation of 3,4-*seco* or 2,3-*seco-*triterpenoids, which are common intermediates in triterpenoid biosynthesis. Several *Seco-*tirucallane triterpenoids have been isolated from *Aphanamixis grandifolia*, demonstrating the occurrence of these transformations in nature. When further oxidative alteration of *seco-*precursors results in side-chain truncation or carbon–carbon bond loss, degraded tirucallanes are produced. Oxidative cleavage at C-21/C-22 or C-23/C-24 is one of these reactions, as well as the conversion of intact C30 tirucallanes into C26 or C29 congeners. Analogous C24 and C26 compounds from *Aphanamixis* confirm oxidative side-chain shortening as a prevalent biosynthetic route, despite degraded tirucallanes being significantly represented in Meliaceae literature [21].

Heteroatom-containing tirucallanes are formed when oxidation at C-1, C-2, C-17, C-21, or C-23 generates carbonyl or epoxide functionalities. In *Dysoxylum lenticellatum*, several tirucallanes contain a hemiketal moiety, formed by cyclisation between a carbonyl at C-23 and proximal hydroxyl groups, while others feature a six-membered lactone ring at C-17, demonstrating oxygen-based heterocycle formation on tirucallane core. Highly rearranged tirucallanes, including those bearing *abeo*-type skeletons, have been isolated from plants, showing that significant carbon–carbon bond reorganization can occur in this class of triterpenoids. For example, dysolenticin A (**365**) from *Dysoxylum lenticellatum* is explicitly characterized as a rearranged skeleton tirucallane, providing clear evidence for the natural occurrence of *abeo*-tirucallane frameworks, comprising major carbon–carbon bond reconfiguration [22]. Biosynthetic relationships underlying the major tirucallane framework types reported in Meliaceae family are shown in Fig. 1.

Tirucallane-type triterpenoids originate through enzymatic cyclization of squalene in the mevalonate pathway, producing a C30 tetracyclic nucleus analogous to sterol frameworks [23], which also serves as a biosynthetic precursor for limonoids, a hallmark of Meliaceae secondary metabolism [24]. Tirucallane skeleton is characterized by an A–D ring tetracyclic core with Δ⁷,⁸ or Δ^24^,^25^ double bonds and diverse oxidative substituents including hydroxyl, carbonyl, and carboxyl groups [25]. Structural diversification arises from oxidative tailoring, skeletal rearrangement, and glycosylation. Hydroxylation at C-3, C-21, or C-24 enhances polarity and may facilitate biomolecular interactions [26], while carbonyl substitution at C-3 or C-21 is often associated with cytotoxic or anti-inflammatory effects. Carboxylation at C-21 is connected to hepatoprotective and immunomodulatory properties [27]. Glycosylated tirucallanes, particularly from *Aphanamixis* and *Dysoxylum,* also have enhanced solubility that may influence pharmacokinetics [28].

Functionalization patterns vary substantially among *Meliaceae* genera, with *Azadirachta indica* producing highly oxidized tirucallanes such as 3-oxo-tirucalla-7,24-dien-21-oic acid, while *Melia azedarach* generates predominantly hydroxylated congeners including tirucalla-7,24-dien-3β-ol [29]. *Toona ciliata* and *Aglaia odorata* frequently yield ketone-bearing derivatives, namely tirucalla-8,24-dien-3-one and aglaiol [30]. These inter-generic differences reflect enzymatic diversification in tirucallane biosynthetic pathways. From structure–activity relationship (SAR) perspective, hydroxylation at C-3 contributes to antimicrobial membrane interactions, while double-bond positions at C-7,8 or C-24,25 alter molecular conformation and influence cytotoxic potential [31]. Collectively, these features underscore the critical role of structural modification in shaping biological properties of tirucallane-type triterpenoids.

**Fig. 2:**
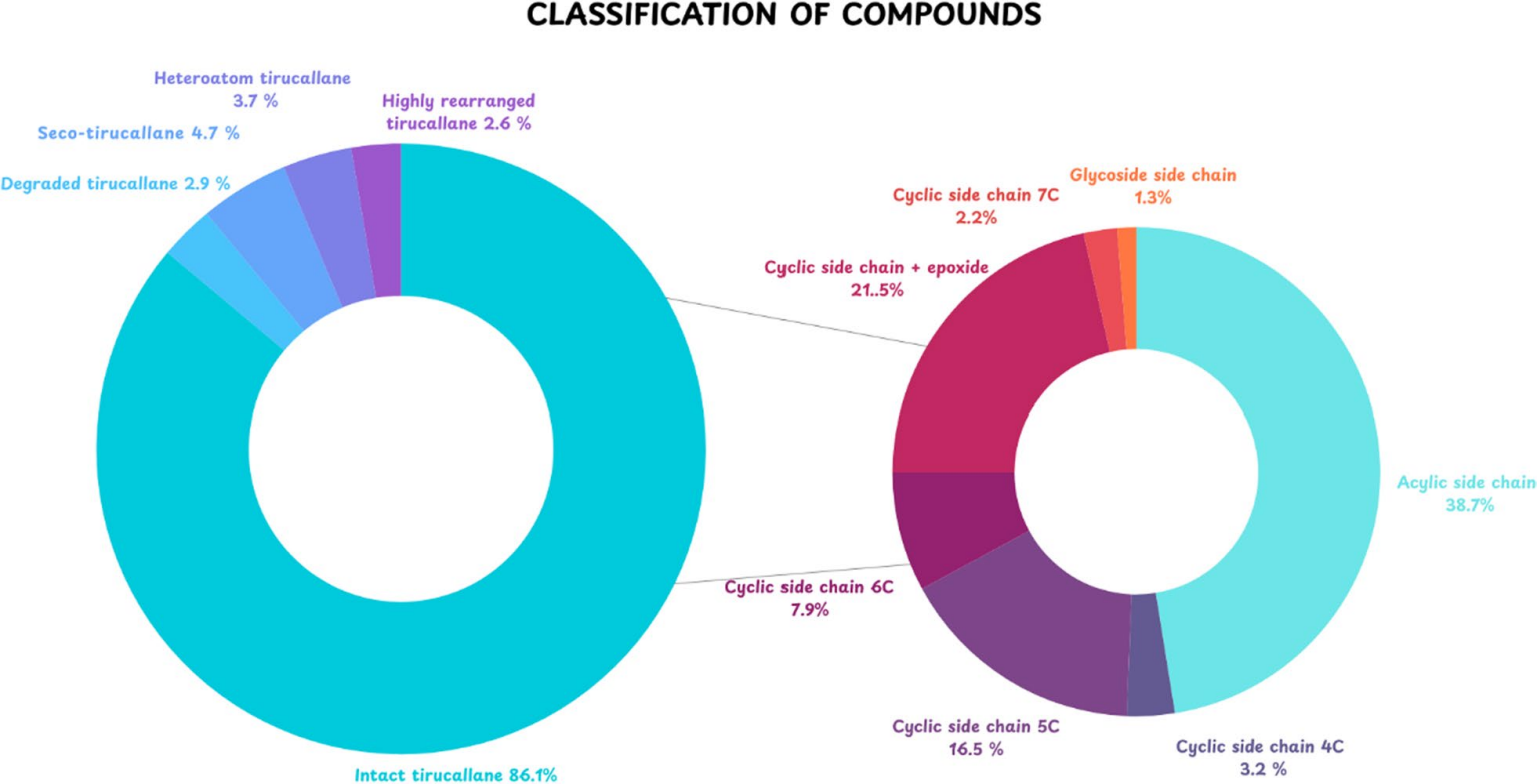
Classification of tirucallane-type triterpenoids from the Meliaceae family

The structural distribution of the compiled metabolites is shown in Fig. 2. Intact tirucallane framework constitutes the predominant category (86.1%), followed by seco (4.7%), heteroatom-containing (3.7%), degraded (2.9%), and highly rearranged (2.6%). In the intact category, diversification is driven primarily by C-17 side-chain architecture, with acyclic side-chain derivatives representing the largest subclass (38.7%), followed by cyclic side-chain plus epoxide (21.5%), cyclic side-chain 5C (16.5%), cyclic side-chain 6C (7.9%), cyclic side-chain 4C (3.2%), cyclic side-chain 7C (2.2%), and glycoside side-chain derivatives (1.3%). Overall, these distributions indicate that side-chain remodelling and oxidative tailoring are central drivers of tirucallane diversification in Meliaceae.

### Intact tirucallane-type

**Fig. 3:**
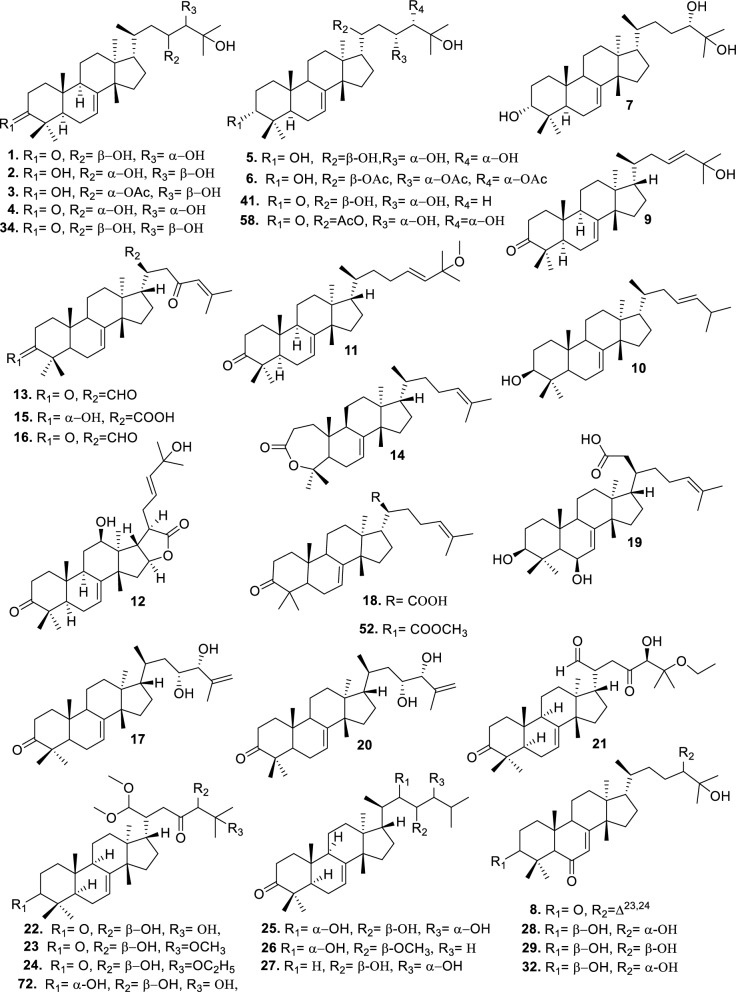

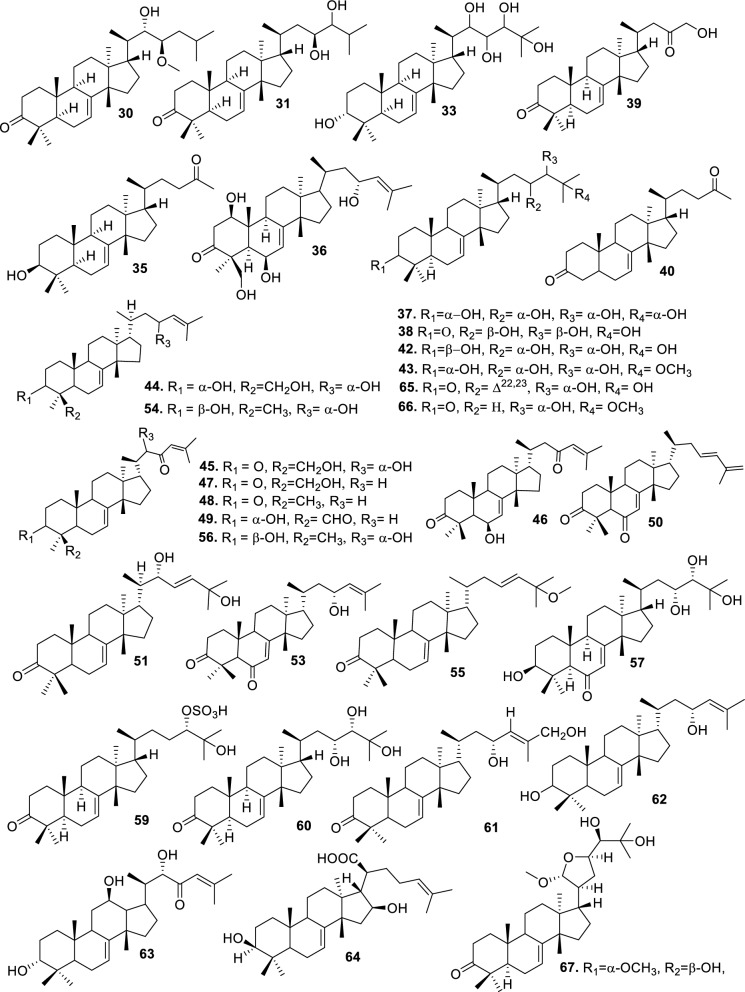

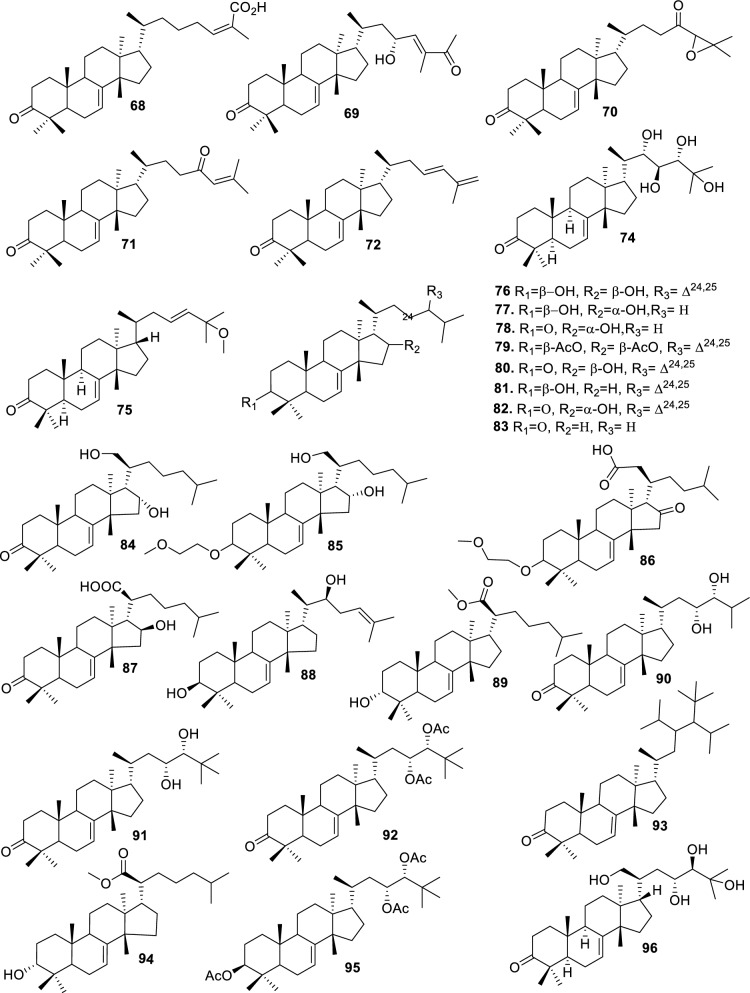

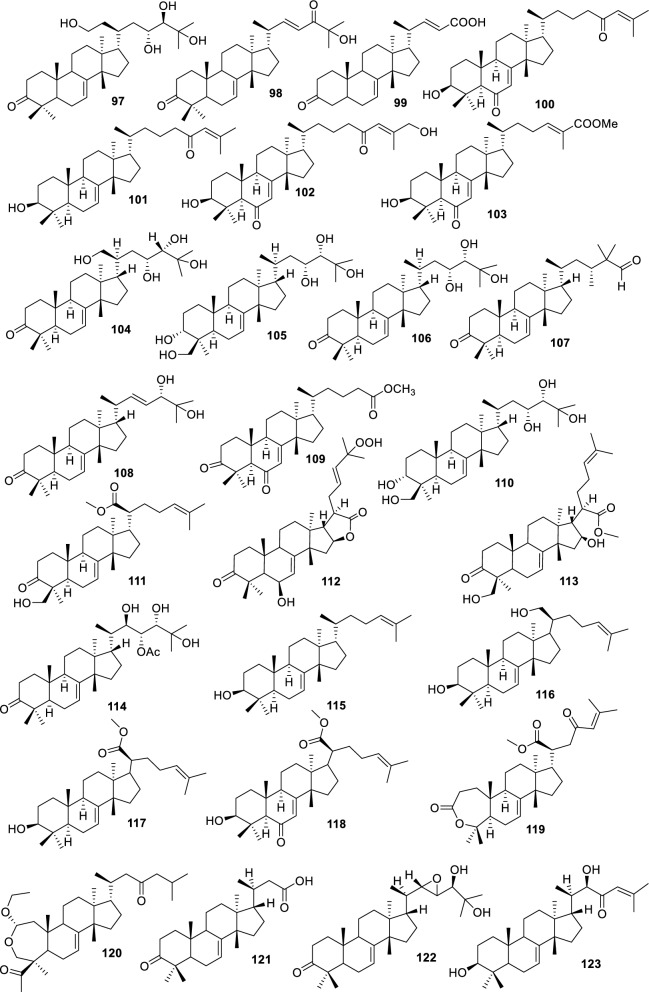

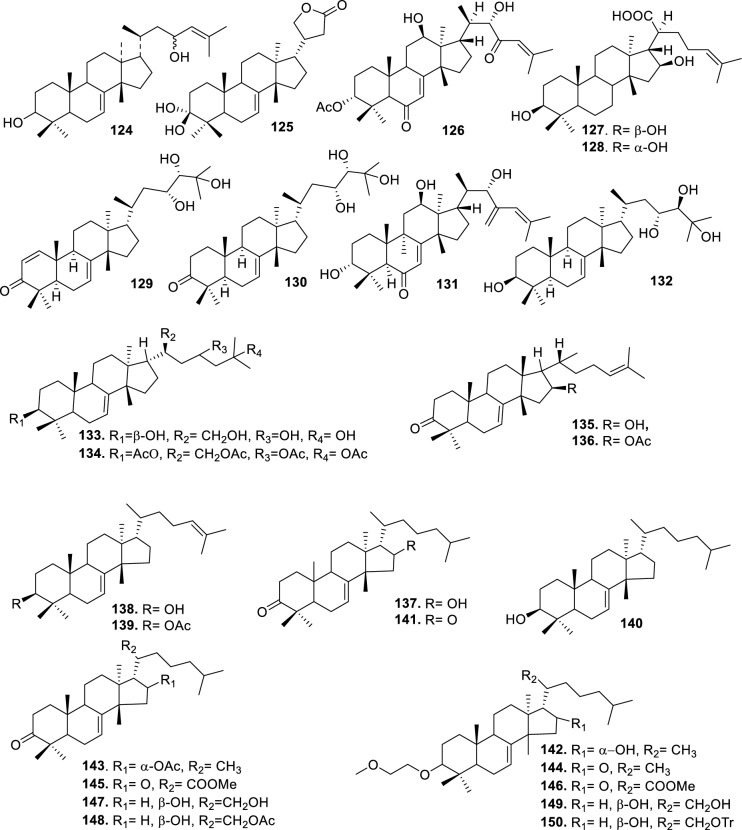
Intact tirucallane-type (Acyclic side-chain derivatives **1–150**)

**Fig. 4:**
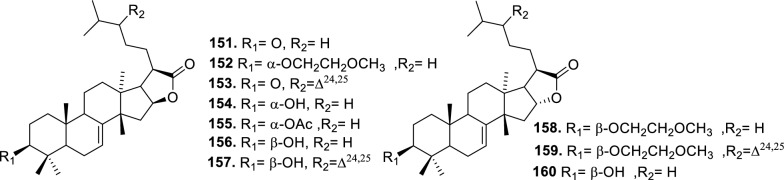
Intact tirucallane-type (Acyclic side-chain 4C derivatives **151–160**)

**Fig. 5:**
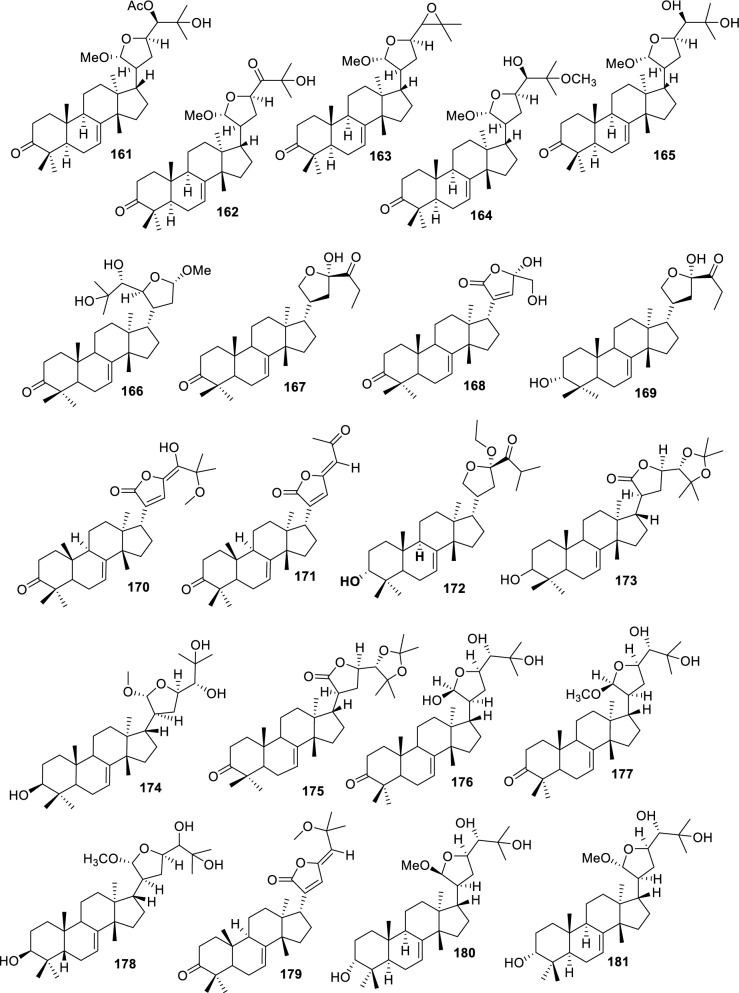

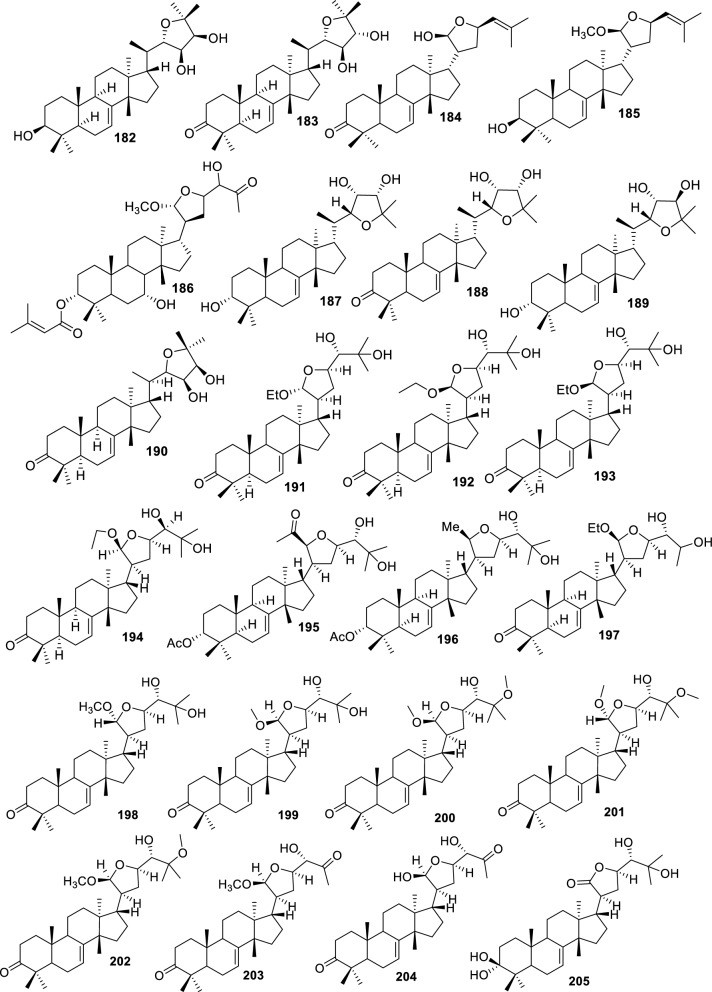

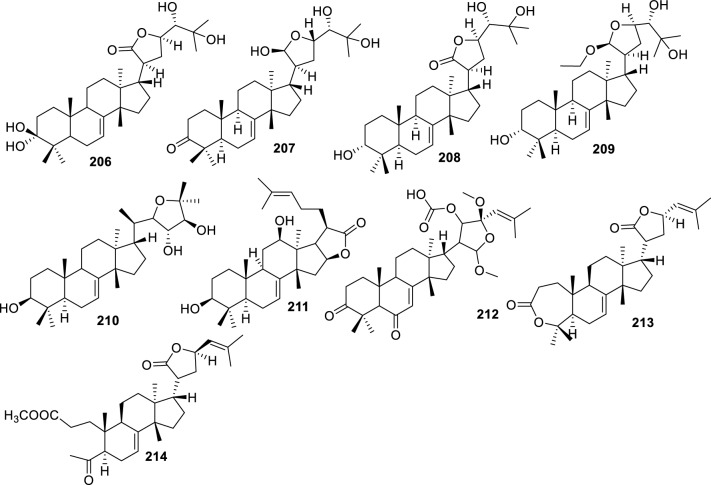
Intact tirucallane-type (Acyclic side-chain 5C derivatives **161–214**)

**Fig. 6:**
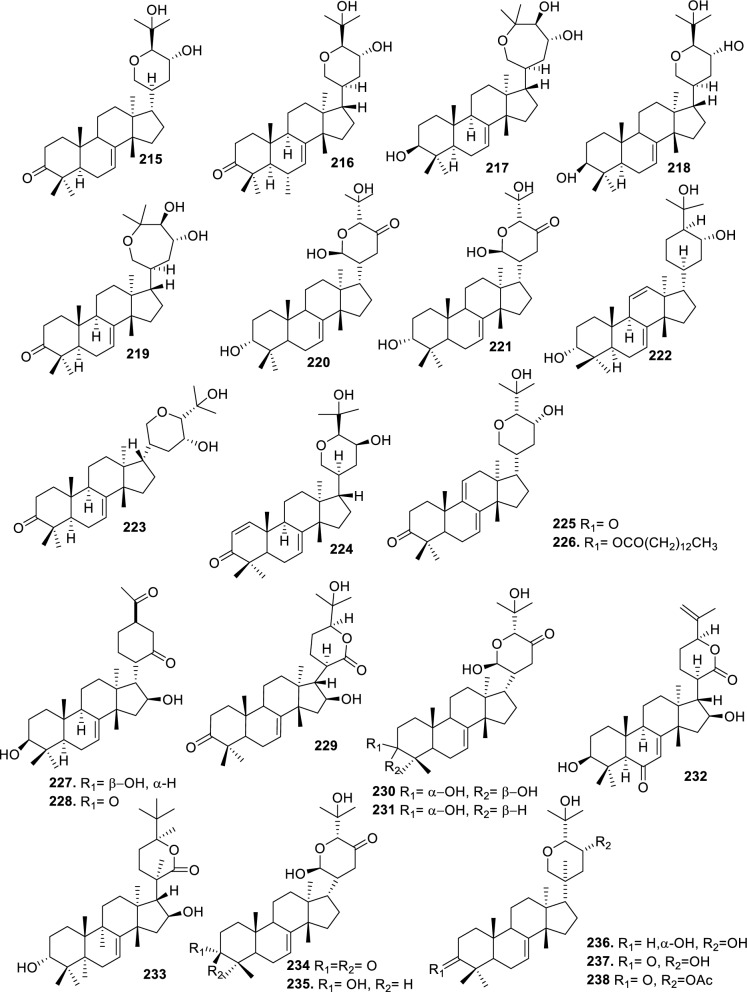
Intact tirucallane-type (Cyclic side-chain 6C derivatives **215–238**)

**Fig. 7:**
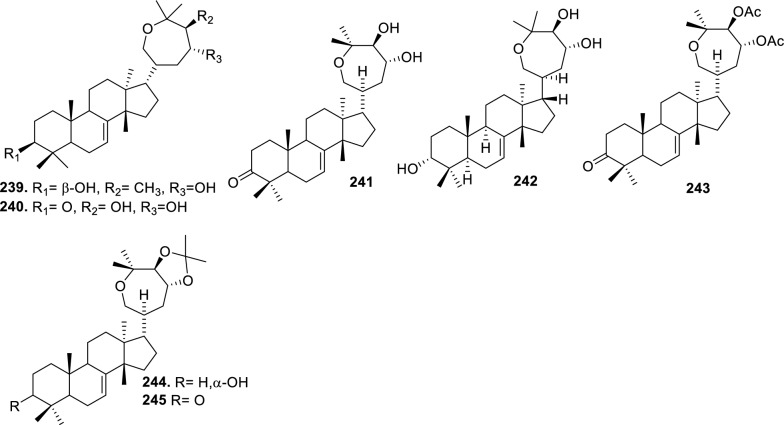
Intact tirucallane-type (Cyclic side-chain 7C derivatives **239–245**)

**Fig. 8:**
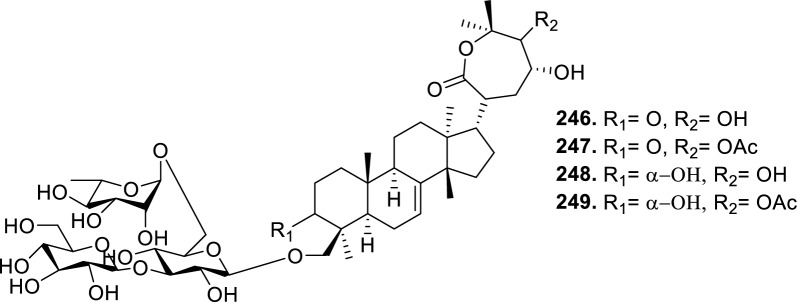
Intact tirucallane-type (Cyclic–glycoside conjugates **246–249**)

**Fig. 9:**
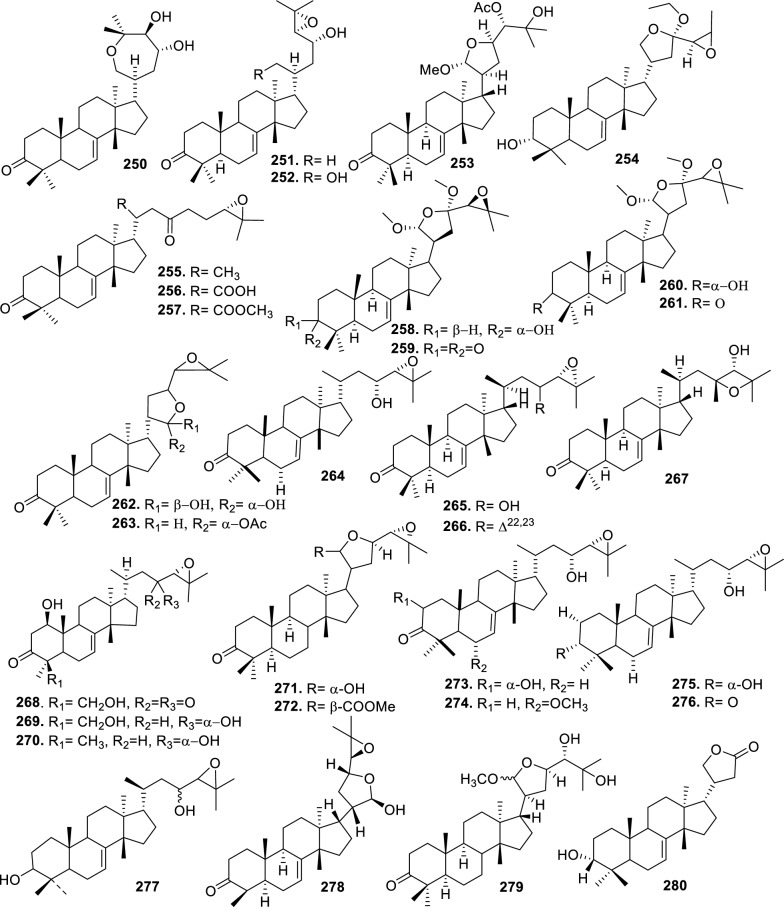

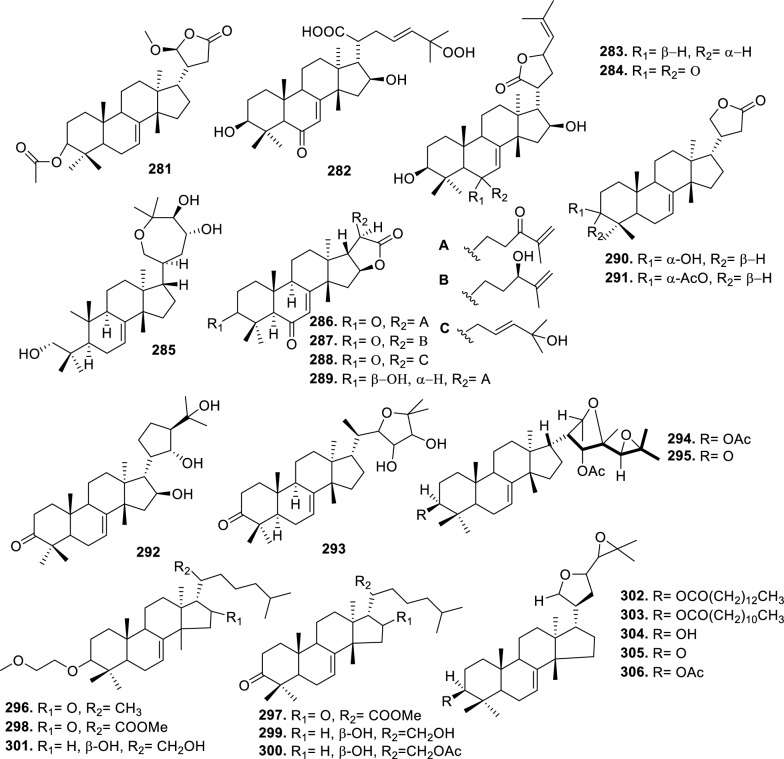

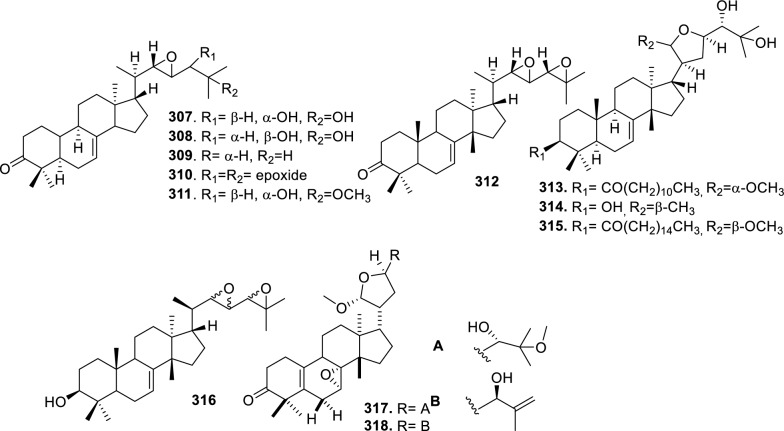
Intact tirucallane-type (Epoxide-modified cyclic side-chain **250–318**)

Based on the structural variation of C-17 side chain, a total of 318 intact tirucallane-type triterpenoids were classified into eight subclasses, with the main categories being acyclic, cyclic, and epoxide derivatives. Additionally, there are minor subclasses, including cyclic side-chain derivatives with 4 carbon (4C), 5 carbon (5C), 6 carbon (6C) or 7 carbon (7C) atoms in the side chain, as well as cyclic–glycoside conjugates. Acyclic side-chain derivatives represent the largest group, consisting of 150 compounds (**1–150**), followed by the cyclic side-chain 5C subgroup, which includes 54 compounds (**161–214**), and the epoxide-modified cyclic side-chain subgroup comprising 68 compounds (**250–318**). Other minor subclasses are cyclic side-chain 4C derivatives (10 compounds; **151–160**), cyclic side-chain 6C derivatives (25 compounds; **215–238**), cyclic side-chain 7C derivatives (7 compounds; **239–245**), and cyclic–glycoside conjugates (4 compounds; **246–249**). These results underscore the predominance of acyclic and epoxidized cyclic derivatives, suggesting that side-chain oxidative transformation plays a crucial role in the structural diversification of tirucallane scaffolds in Meliaceae family (Table 1, Figs. 3, 4, 5, 6, 7, 8, 9).

**Table 1:** Intact tirucallane compounds from Meliaceae family

No	Compounds	Species	Parts	Extract	References
**1**	Piscidinol A	*Entandrophragma congoënse*	Root	CH_2_Cl_2_/MeOH	[32]
**2**	Hispidol A	*Trichilia hispida*	Leaves	EtOH	[33]
**3**	Hispidol B	*T. hispida*	Leaves	EtOH	[33]
**4**	Bourjotinolone C	*T. hispida*	Leaves	EtOH	[33]
**5**	Sapelin F	*T. hispida*	Leaves	EtOH	[33]
**6**	Sapelin F tetraacetate	*T. hispida*	Leaves	EtOH	[33]
**7**	3*S*,24*S*,25-trihydroxytirucall-7-ene	*T. hispida*	Leaves	EtOH	[33]
**8**	Aphanamgrandin K	*Aphanamixis grandifolia*	Stems	EtOH	[34]
**9**	(23*Z*)-25-hydroxy-tirucalla-7,23-diene-3-one	*A. grandifolia*	Stems	EtOH	[34]
**10**	3β,25-dihydroxy-tirucalla-7,23-diene	*A. grandifolia*	Stems	EtOH	[34]
**11**	Leucophyllone	*A. grandifolia*	Stems	EtOH	[34]
**12**	Indicalilacol D	*Azadirachta indica*	Fruit	MeOH	[36]
**13**	3,23-dioxotirucalla-7,24-dien-21-al	*Entandrophragma Angolese*	Leaves	*n-*hexane	[37]
**14**	Entandrolide	*E. Angolese*	Seeds	*n-*hexane	[37]
**15**	Congoensin B	*Entandrophragma congoënse*	Stem bark	CH_2_Cl_2_/MeOH	[39]
**16**	3,23-dioxotirucalla-7,24-dien-21-acid	*E. congoënse*	Stem bark	CH_2_Cl_2_/MeOH	[39]
**17**	Bourjotinolone B	*T. sinensis*	Stem bark	EtOH	[38]
**18**	Methyl 3β-hydroxy-tirucalla-7,24-dien-21-oate	*A. grandifolia*	Stem bark	EtOH	[40]
**19**	Methyl (3β,5β,9β)-3,5-dihydroxytirucalla-7,24-dien-21-oate	*A. grandifolia*	Stem bark	EtOH	[40]
**20**	(3*R*,5*R*,9*R*,10*S*,13*S*,14*R*,17*S*)-9-hydroxy-10,13-dimethyl-3-oxo-2,3,4,5,6,7,8,9,11,12,14,15,16,17-tetradecahydro-1H-cyclopenta[a]phenanthrene-17-carboxylate	*A. grandifolia*	Stem bark	EtOH	[40]
**21**	3-oxo-urs-12-en-28-oic acid 17β-O-[3-(1-ethoxy-1-hydroxy-2-methyl)propionyl] ester	*A. grandifolia*	Stem bark	EtOH	[40]
**22**	3-oxo-urs-12-en-28-oic acid 17β-O-[3-(hydroxy-2-methylpropanoyl)oxy]propyl ether (dimethoxy substituted)	*A. grandifolia*	Stem bark	EtOH	[40]
**23**	3-oxo-urs-12-en-28-oic acid 17β-O-[2-(1-hydroxy-2-methylpropanoyl)oxy]propyl ester	*A. grandifolia*	Stem bark	EtOH	[40]
**24**	3-oxo-urs-12-en-28-oic acid 17β-O-[2-(1-hydroxy-2-methylpropoxy)propionyl] ester	*A. grandifolia*	Stem bark	EtOH	[40]
**25**	Xylocarpol A	*Xylocarpus granatum, Xylocarpus moluccensis*	Seeds, stems and twigs	EtOH	[41]
**26**	Xylocarpol B			EtOH	[41]
**27**	Xylocarpol C			EtOH	[41]
**28**	Agallochol A	*X. granatum, X. moluccensis*	Seeds, stems and twigs	EtOH	[41]
**29**	Agallochol B	*X. granatum, X. moluccensis*	Seeds, stems and twigs	EtOH	[41]
**30**	Agallochol C	*X. granatum, X. moluccensis*	Seeds, stems and twigs	EtOH	[41]
**31**	Agallochol D	*X. granatum, X. moluccensis*	Seeds, stems and twigs	EtOH	[41]
**32**	Dysoxyhaine D	*Dysoxylum hainanense*	Stem barks	EtOAc	[43]
**33**	Pentaol	*Cedrela glaziovii, Cedrela odorata, Cedrela fissilis*	Heartwood, and stems	EtOH	[25]
**34**	3-oxo-threo-23,24,25-trihydroxy-tirucall-7-ene	*C. odorata*	Heartwood	EtOH	[25]
**35**	Toonaciliatin L	*Toona ciliata* var.* ciliata*	Leaves and twigs	EtOH	[24]
**36**	Piscidinal A	*C. odorata*	Leaves and twigs	EtOH	[25]
**37**	Piscidinol B	*T. ciliata* var.* henryi*	Stem barks	EtOH	[25]
**38**	25-methoxyhispidol	*C. odorata*	Leaves and twigs	EtOH	[25]
**39**	Dysomollide A	*Dysoxylum macranthum*	Stem barks	EtOH	[26]
**40**	Dymacrin A	*D. macranthum*	Stem barks	EtOH	[26]
**41**	Dymacrin B	*D. macranthum*	Stem barks	EtOH	[26]
**42**	Dymacrin C	*D. macranthum*	Stem barks	EtOH	[26]
**43**	Dymacrin D	*D. macranthum*	Stem barks	EtOH	[26]
**44**	Dymacrin E	*D. macranthum*	Stem barks	EtOH	[26]
**45**	Dymacrin F	*D. macranthum*	Stem barks	EtOH	[26]
**46**	Dymacrin G	*D. macranthum*	Stem barks	EtOH	[26]
**47**	Methyl kulonate	*Melia azedarach*	Fruits	MeOH	[27]
**48**	Dyvariabilin A	*Dysoxylum variabile*	Stem bark	MeOH	[28]
**49**	Tirucalla-7,24-diene-3β,23-diol	*D. variabile*	Stem bark	MeOH	[28]
**50**	Tirucalla-7,23,25-triene-3,6-dione	*D. variabile*	Stem bark	MeOH	[28]
**51**	Tirucalla-7,23,25-trien-3β-ol	*D. variabile*	Stem bark	MeOH	[28]
**52**	2α-ethoxy-2,3-secotirucalla-2,29-epoxy-7-ene-23-oxo-3-oic acid	*Aphanamixis grandifolia*	Leves and root	EtOH	[31]
**53**	(23*E*)-2α-hydroxytirucall-7,23,25-triene-3-one	*A. grandifolia*	Leves and root	EtOH	[31]
**54**	2,3-*seco-*tirucalla-2,3;2,29-diepoxy-7-ene-3,23-dione	*A. grandifolia*	Leves and root	EtOH	[31]
**55**	25-methoxy-tirucall-7-23 (trans) diene-3-one	*Aglaia Leucophylla*	Stem barks	MeOH	[44]
**56**	3β,22*S*-dihydroxy-tirucalla-7,24-dien-23-one	*Dysoxylum hainanense*	Stem bark	MeOH	[23]
**57**	23,26-dihydroxy-tirucalla-7,24-dien-3-one	*D. hainanense*	Stem bark	MeOH	[23]
**58**	3β,23-dihydroxy-tirucalla-7,24-diene	*D. hainanense*	Stem bark	MeOH	[23]
**59**	3β,24-dihydroxy-tirucalla-7,25-diene, 24-sulfate	*Trichilia maynasiana*	Leaves	MeOH	[45]
**60**	21-oxo-melianodiol 14 21-oxo-meliantriol 15 toosendanic acid A	*T. maynasiana*	Leaves	MeOH	[45]
**61**	Meliastatin	*Melia azedarach*	Fruit	MeOH	[27]
**62**	3-epimesendanin S 12-acetate	*Walsura trichostemon*	Roots	EtOAc, MeOH	[47]
**63**	3-epimesendanin S	*W. trichostemon*	Roots	EtOAc, MeOH	[47]
**64**	Meliasenin G	*Melia toosendan*	Stem bark	EtOH	[48]
**65**	Chisopanin M	*Chisocheton paniculatus*	Twigs	EtOH	[49]
**66**	Phellochin	*Chisocheton cumingianus* subsp.* balansae*	Twigs	EtOH	[49]
**67**	21α-methylmeliandiol	*Toona sinensis*	Bark	MeOH	[50]
**68**	Chisopaten A	*Chisocheton patens Blume*	Stem bark	MeOH	[14]
**69**	Chisocarpene A	*Chisocheton lasiocarpus*	Stem bark	MeOH	[14]
**70**	24,25-epoxy-tirucall-7-ene-3,23-dione	*Amoora dasyclada*	Stems	MeOH	[51]
**71**	22*E*-hydroxytirucalla-7,24-dien-3,3-dione	*Amoora tetrapetala*	Branches	MeOH	[51]
**72**	3α,24β,25-trihydroxy-21,21-dimethoxy-23-oxotirucall-7-ene	*Aphanamixis grandifolia*	Stem barks	CDCl_3,_MeOH	[52]
**73**	(23*E*)-2α-hydroxytirucalla 7,23,25-triene-3-one	*A. grandifolia*	Leaves and twigs	EtOH	[53]
**74**	Toonaciliatavarin E	*Toona Ciliatawas*	Bark	MeOH	[54]
**75**	( −)-Leucophyllone	*Aglaia leucophylla*	Bark	MeOH	[55]
**76**	16-hydroxybutyrospermol	*Melia azedarach*	Bark	*n*-hexane	[37]
**77**	Eupho-7,26-dien-3β,16β-diol diacetate	*M. azedarach*	Bark	*n*-hexane	[37]
**78**	Eupha-7-en-3β,16β-diol	*M. azedarach*	Bark	*n*-hexane	[37]
**79**	16 α,21-dihydroxyeupha-7,24-dien-3-one diacetate	*M. azedarach*	Bark	*n*-hexane	[37]
**80**	16β-hydroxyeupha-7,24-dien-3-one	*M. azedarach*	Bark	*n*-hexane	[37]
**81**	3β,16β-dihydroxy-eupha-7,24-diene	*M. azedarach*	Bark	*n*-hexane	[37]
**82**	16-epikulinone	*M. azedarach*	Bark	*n*-hexane	[37]
**83**	Butyrospermone	*M. azedarach*	Bark	*n*-hexane	[37]
**84**	16α,21-dihydroxyeupha-7,24-dien-3-one	*M. azedarach*	Bark	*n*-hexane	[37]
**85**	16α,21-dihydroxyeupha-7,24-dien-3-one ethylene ketal	*M. azedarach*	Bark	*n*-hexane	[37]
**86**	21-hydroxyeupha-7,24-dien-3,16-dione 3-ketal	*M. azedarach*	Bark	*n*-hexane	[37]
**87**	Kulonic acid	*M. azedarach*	Bark	*n*-hexane	[37]
**88**	Eupha-7,24-diene	*M. azedarach*	Bark	*n*-hexane	[37]
**89**	Methyl 3-oxotirucalla-8,24-dien-21-oate	*M. azedarach*	Bark	*n*-hexane	[37]
**90**	Piscidinol A acetyl	*Walsura piscidia*	Leaves	*n*-hexane, EtOAc	[38]
**91**	7,9(11)-tirucalladiene	*W. piscidia*	Leaves	*n*-hexane, EtOAc	[38]
**92**	Piscidinol A acetonide	*W. piscidia*	Leaves	*n*-hexane, EtOAc	[38]
**93**	Acetonide	*W. piscidia*	Leaves	*n*-hexane, EtOAc	[38]
**94**	Lupenone aldehyde	*W. piscidia*	Leaves	*n*-hexane, EtOAc	[38]
**95**	7α,12α,21-triacetoxy-4,4,8,10,13-pentamethyl-17-(2,2-dimethylpropionyloxy)-pregnane	*W. piscidia*	Leaves	*n*-hexane, EtOAc	[38]
**96**	24-epi-piscidinol A	*Aglaia andamanica*	Leaves	MeOH	[58]
**97**	Aglaiodiol	*A. andamanica*	Leaves	MeOH	[58]
**98**	Moluccensin P	*A. andamanica*	Leaves	MeOH	[58]
**99**	Moluccensin Q	*A. andamanica*	Leaves	MeOH	[58]
**100**	3β-hydroxytirucalla-7,24-diene-6,23-dione	*Dysoxylum lukii*	Stem bark	EtOH	[59]
**101**	3β-hydroxytirucalla-7,24-dien-23-one	*D. lukii*	Stem bark	EtOH	[59]
**102**	3β,26-dihydroxytirucalla-7,24-diene-6,23-dione	*D. lukii*	Stem bark	EtOH	[59]
**103**	Methyl 6-oxomasticadienolate	*D. lukii*	Stem bark	EtOH	[59]
**104**	Toonapubesin D	*Toona ciliata* var *Pubescens*	Stem bark	MeOH	[60]
**105**	Toonapubesin E	*T. ciliata* var *Pubescens*	Stem bark	MeOH	[60]
**106**	Toonapubesin F	*T. ciliata* var *Pubescens*	Stem bark	MeOH	[60]
**107**	Toonapubesin G	*T. ciliata* var *Pubescens*	Stem bark	MeOH	[60]
**108**	Dihydroniloticin	*T. ciliata* var *Pubescens*	Stem bark	MeOH	[60]
**109**	Methyl-24,25,26,27-tetranortirucall-7-en-3-oxo 23-oate	*T. ciliata* var *Pubescens*	Stem bark	MeOH	[60]
**110**	24*S*,25-dihydroxytirucall-7-en-3-one	*T. ciliata* var *Pubescens*	Stem bark	MeOH	[60]
**111**	Meliasenin E	*Melia azedarach*	Fruit	EtOAc	[61]
**112**	Meliasenin A	*Melia toosendan*	Stem bark	EtOH	[48]
**113**	Meliasenin H	*M. toosendan*	Stem bark	EtOH	[48]
**114**	Hispidol C	*Chukrasia tabularis*	Fruit	EtOH	[42]
**115**	Tirucalla-7,24-dien-3β-ol	*C. tabularis*	Fruit	EtOH	[42]
**116**	(3*S*,5*R*,9*R*,10*R*,13*S*,14*S*,16*S*,17*S*,20*S*)-3,16,21-trihydroxy-tirucalla-7,24-diene	*Melia toosendan*	Fruits	MeOH	[62]
**117**	(3*S*,5*R*,9*R*,10*R*,12*R*,13*S*,14*R*,16*S*,17*S*,20*S*)-3,12-dihydroxy-tirucallane-7,24-diene-21-Methyl ester	*M. toosendan*	Fruits	MeOH	[62]
**118**	Toosendine E	*M. toosendan*	Fruits	MeOH	[62]
**119**	Entanolide	*E. angolense*	Bark	*n*-hexane, EtOAc	[16]
**120**	2α-ethoxy-2,3 *seco*tirucalla-2,29-epoxy-7-ene-23-oxo-3-oic acid	*Aphanamixis grandifolia*	Leaves and twigs	EtOH	[53]
**121**	4,4,14-trimethyl3-oxo-24-nor-5α,13α,14β,17α,20S-chol-7-en-23-oic	*D. chaudianum*	Stem bark	*n*-hexane	[63]
**122**	Toonapubesin B	*D. chaudianum*	Stem bark	*n*-hexane	[63]
**123**	3β,22*S*-dihydroxy-tirucalla-7,24-dien-23-one	*D. chaudianum*	Stem bark	*n*-hexane	[63]
**124**	(3β)-22,23-epoxytirucall-7-ene-3,24,25-triol	*Munronia delavayi*	Leaves	EtOH	[64]
**125**	3-oxo-24,25,26,27-tetranortirucall-7-ene-23(21)-lactone	*A. dasyclada*	Stems	CDCl_3,_MeOH	[52]
**126**	Trichostemonoate	*Walsura trichostemon*	Stem bark	EtOAc	[74]
**127**	Toosendanone acid A	*M. toosendan*	Bark	EtOH	[66]
**128**	Toosendanone Acid B	*M. toosendan*	Bark	EtOH	[66]
**129**	Toonamicrocarpavarin	*Toona Ciliatawas*	Bark	MeOH	[54]
**130**	Phellocin	*T. Ciliatawas*	Bark	MeOH	[54]
**131**	Trichostemonol	*W. trichostemon*	Twigs	CH_2_Cl_2_	[69]
**132**	3β-hydroxy-3-decarbonyl-24-epi-piscidinol A	*Xylocarpus moluccensis*	Seeds	EtOH	[67]
**133**	Tetraol diene	*M. azedarach* L	Fruits	CHC1_3_	[70]
**134**	Bromoacetal	*M. azedarach* L	Fruits	CHC1_3_	[70]
**135**	Kulinone	*M. azedarach* L	Bark	C_6_H_14_	[37]
**136**	Kulinone acetate	*M. azedarach* L	Bark	C_6_H_14_	[37]
**137**	7,9(11)-heteroannular diene euphane type	*M. azedarach* L	Bark	C_6_H_14_	[37]
**138**	Butyrospermol	*M. azedarach* L	Bark	C_6_H_14_	[37]
**139**	Dihydro butyrospermol acetate	*M. azedarach* L	Bark	C_6_H_14_	[37]
**140**	Euphenol	*M. azedarach* L	Bark	C_6_H_14_	[37]
**141**	16-dehydrokulinone	*M. azedarach* L	Bark	C_6_H_14_	[37]
**142**	3-ketal derivative	*M. azedarach* L	Bark	C_6_H_14_	[37]
**143**	16-epikulinone acetate	*M. azedarach* L	Bark	C_6_H_14_	[37]
**144**	16-dehydrokulinone 3-ethylene ketal	*M. azedarach* L	Bark	C_6_H_14_	[70]
**145**	Methyl 16-dehydrokulonate	*M. azedarach* L	Bark	C_6_H_14_	[70]
**146**	Methyl 16-dehydrokulonate 3-ketal	*M. azedarach* L	Bark	C_6_H_14_	[70]
**147**	16β,21-dihydroxyeupha-7,24-dien-3-one	*M. azedarach* L	Bark	C_6_H_14_	[70]
**148**	16α,21-dihydroxyeupha-7,24-dien-3-one diacetate	*M. azedarach* L	Bark	C_6_H_14_	[70]
**149**	16β,21-dihydroxyeupha-7,24-dien-3-one 3-ketal,21-trityl ether	*M. azedarach* L	Bark	C_6_H_14_	[70]
**150**	16β21-dihydroxyeuphu-7,24-dien-3-one 3-ketal,21-trityl ether	*M. azedarach* L	Bark	C_6_H_14_	[70]
**151**	Kulactone	*Melia azedarach*	Bark	C_6_H_14_	[37]
**152**	Kulactone ketal	*M. azedarach*	Bark	C_6_H_14_	[37]
**153**	24,25-dihydrokulactone	*M. azedarach*	Bark	C_6_H_14_	[37]
**154**	Kulolactone	*M. azedarach*	Bark	C_6_H_14_	[37]
**155**	Kulolactone acetate	*M. azedarach*	Bark	C_6_H_14_	[37]
**156**	24,25-dihydro-3-epikulolactone	*M. azedarach*	Bark	C_6_H_14_	[37]
**157**	24,25-dihydro-14d-epoxide kulactone	*M. azedarach*	Bark	C_6_H_14_	[37]
**158**	16-epikulactone 3-ethylene ketal	*M. azedarach*	Bark	C_6_H_14_	[37]
**159**	24,25-dyhidro-16-epikulactone	*M. azedarach*	Bark	C_6_H_14_	[37]
**160**	3-epi-16-epikulactone	*M. azedarach*	Bark	C_6_H_14_	[37]
**161**	(+)-21*R*,23*R*-epoxy-21α-methoxy-24*S*,25-dihydro xyapotirucall-7-en-3-one	*Dysoxylum binectariferum*	Stem bark	EtOH	[71]
**162**	(+)-21*R*,23*R*-epoxy-21α methoxy-25-hydroxyapotirucall-7-en-3,24-dione	*D. binectariferum*	Stem bark	EtOH	[71]
**163**	(+)-21*R*,23*R*-epoxy-21α-methoxy-24,25-dihy droxyapotirucall-7-en-3-one	*D. binectariferum*	Stem bark	EtOH	[71]
**164**	(+)-21*R*,23*R*-epoxy-21α,25-dimethoxy-24-hydroxyapotirucall-7-en-3-one	*D. binectariferum*	Stem bark	EtOH	[71]
**165**	Methyl 3,21,22-trihydroxyolean-12-en-28-oate	*Cedrela sinensis*	Cortex	MeOH	[72]
**166**	21α-methoxy-24,25-dihydroxyapotirucall-7-en-3-one	*C. sinensis*	Cortex	MeOH	[72]
**167**	Dysolenticin C	*C. sinensis*	Cortex	MeOH	[72]
**168**	Dysolenticin D	*C. sinensis*	Cortex	MeOH	[72]
**169**	Dysolenticin E	*C. sinensis*	Cortex	MeOH	[72]
**170**	(13α,14β,17α,23*Z*)-25-methoxy-21,23-epoxylanosta-7,20(22),23-triene-3,21-dione	*Aphanamixis grandifolia*	Stem bark	EtOH	[73]
**171**	(13α,14β,17β,23*Z*)-21,23-epoxylanosta-7,20(22),23,25-tetraene-3,21-dione	*A. grandifolia*	Stem bark	EtOH	[73]
**172**	(3α,13α,14 β,17α,20*S*,23*R*)-23-ethoxy-3-hydroxy-21,23-epoxylanost-7-en-24-one	*A. grandifolia*	Stem bark	EtOH	[73]
**173**	3-hydroxy*-*21-oxo-melianodiol 24,25-acetonide	*Azadirachta indica*	Fruit	MeOH	[36]
**174**	21-oxo-melianodiol 24,25-acetonide	*A. indica*	Fruit	MeOH	[36]
**175**	Meliasenin S	*A. indica*	Fruit	MeOH	[36]
**176**	Meliantriol	*A. indica*	Fruit	MeOH	[36]
**177**	Meliasenin T	*A. indica*	Fruit	MeOH	[36]
**178**	Indicalilacol C	*A. indica*	Fruit	MeOH	[36]
**179**	Methyl (3*R*,5*S*,10*S*,13*R*,14*R*,17*S*)-10,13-dimethyl-3-oxo-2,3,4,5,6,7,8,9,11,12,14,15,16,1tetradecahydro-1H-cyclopenta[a]phenanthrene-17-carboxylate	*Aphanamixis grandifolia*	Stem bark	EtOH	[40]
**180**	Agladupol D	*Aglaia duperreana*	Leaves and bark	EtOH	[74]
**181**	Agladupol E	*A. duperreana*	Leaves and bark	EtOH	[74]
**182**	Xylocarpol D	*Xylocarpus granatum, Xylocarpus moluccensis*	Seeds, stems and twigs	EtOH	[22]
**183**	Flindssone	*Guarea guidonia*	Aerial	EtOH	[52]
**184**	Picroquassin E	*G. Guidonia*	Aerial	EtOH	[52]
**185**	Toonaciliatine A	*Toona ciliata*	Leaves	*n*-hexane, CDCl_3_	[53]
**186**	Odoratol	*Toona ciliata* var.* henryi*	Stem barks	EtOH/H_2_O	[25]
**187**	6-dehydromexicanol	*Cedrela odorata, Cedrela fissilis*	Heartwood, stems	EtOH/H_2_O	[25]
**188**	Isoodoratol	*C. odorata, C. fissilis*	Heartwood, stems	EtOH/H_2_O	[76]
**189**	22*S*,3α-dihydrotirucalla-7,24-dien-23-one	*C. fissilis*	Stems	EtOH/H_2_O	[76]
**190**	Meliatetraolenone	*T. ciliata* var.* ciliata*	Leaves and twigs	EtOH/H_2_O	[76]
**191**	21α-methylmelianodiol	*C. odorata*	Leaves and twigs	EtOH/H_2_O	[76]
**192**	Toonaciliatin K	*T. ciliata* var.* ciliata*	Leaves and twigs	EtOH/H_2_O	[76]
**193**	21β-ethylmelianodiol	*M. azedarach*	Ripe fruit	EtOH	[30]
**194**	Polystanins C	*Aphanamixis polystachya*	Fruits	EtOH	[77]
**195**	Polystanins D	*A. polystachya*	Fruits	EtOH	[77]
**196**	25-dihydroxyapotirucalla-7-en-3-one	*T. ciliata*	Leave and twigs	EtOH	[78]
**197**	21β-methylmelianodiol	*Melia azedarach*	Ripe fruit	EtOH	[29]
**198**	(21*S*,23*R*)-epoxy-21β,25-dimethoxy	*M. azedarach*	Ripe fruit	EtOH	[29]
**199**	21β,25-dimethylmelianodiol	*M. azedarach*	Ripe fruit	EtOH	[29]
**200**	21, 25-dimethylmelianodiol	*M. azedarach*	Ripe fruit	EtOH	[29]
**201**	(21*R*,23*R*)-epoxy-24-hydroxy-21β-methoxytirucall-7, 25-dien-3-one	*M. azedarach*	Ripe fruit	EtOH	[29]
**202**	Methoxytirucall-7, 25-dien-3-one	*M. azedarach*	Ripe fruit	EtOH	[29]
**203**	21*R*,23*R*-epoxy-21β, 24-dihydroxy-	*M. azedarach*	Ripe fruit	EtOH	[29]
**204**	Tirucall-7, 25-dien-3-one	*M. azedarach*	Ripe fruit	EtOH	[29]
**205**	Melianodiol	*Chisocheton pentandrus*	Stem bark	MeOH	[79]
**206**	Indicalilacol B	*C. pentandrus*	Stem bark	MeOH	[79]
**207**	(3*R*,5*R*,9*R*,10*R*,13*S*,14*S*,17*S*,20*S*,21*S*,23*R*,24*R*)-21-ethoxy-3,24,25-trihydroxy-21,23-epoxy-tirucalla-7-ene	*Melia toosendan*	Fruit	EtOH	[62]
**208**	(3*S*,5*R*,9*R*,10*R*,13*S*,14*S*,17*S*,20*R*,22*R*,23*S*,24*S*)-lanost-7-ene-3,23,24-triol-22,25-epoxy	*M. toosendan*	Fruit	EtOH	[62]
**209**	Cinamodiol	*M. toosendan*	Fruit	EtOH	[62]
**210**	Entandrophin A	*Entandrophragma angolense*	Bark	*n*-hexane, EtOAc	[16]
**211**	Entandrophin B	*E. angolense*	Bark	*n*-hexane, EtOAc	[16]
**212**	Entandrophin C	*E. angolense*	Bark	*n*-hexane, EtOAc	[16]
**213**	3-*O*-[(*S*)-α-methoxy-α-(trifluoromethyl)phenylacetyl] ester	*Melia volkensii*	Root bark	MeOH	[14]
**214**	3-*O*-[(*R*)-α-methoxy-α-(trifluoromethyl)phenylacetyl] ester	*M. volkensii*	Root bark	MeOH	[99]
**215**	2-endo-acetoxy-1-benzyl-1,4-epoxy-1,2,3,4-tetrahydronaphthalene	*Trichilia hispida*	Leaves	EtOH	[71]
**216**	Toonaciliatavarin D	*Toona ciliata* var. *ciliata, Toona ciliata* var.* henryi*	Stem bark, Leaves and Twigs	EtOH	[25]
**217**	3-episapelin A	*T. ciliata* var. *ciliata, T. ciliata* var.* henryi*	Stem bark, leaves and twigs	EtOH	[25]
**218**	Hispidone	*T. ciliata* var. *ciliata, T. ciliata* var.* henryi*	Stem bark, leaves and twigs	EtOH	[25]
**219**	3α,21β, 25-triol-tirucalla-21,24-epoxy-23-one	*Amoora dasyclada*	Twigs	EtOH/H_2_O	[29]
**220**	21β, 25-diol-tirucalla-21,24-epoxy-3, 23-dione	*A. dasyclada*	Twigs	EtOH/H_2_O	[29]
**221**	Sapelinone A	*A. dasyclada*	Twigs	EtOH/H_2_O	[29]
**222**	Bourjotinoline A	*Chisocheton. Paniculatus*	Twigs	EtOH	[49]
**223**	Chisiamols G	*C. paniculatus*	Twigs	EtOH	[30]
**224**	Hispidone diene	*Trichillia hispida*	Leaves	EtOH	[59]
**225**	Lipo-3-episapelin A	*Trichilia Connarozdes*	Root	EtOH	[81]
**226**	Dysoxylumstatin A	*Dysoxylum lukii*	Stem bark	EtOH	[59]
**227**	Dubione B	*D. lukii*	Stem bark	EtOH	[59]
**228**	Meliasenin F	*Melia toosendan*	Stem bark	EtOH	[48]
**229**	2-acetoxy-1-benzyl-naphthalene	*T. hispida*	Leaves	EtOH	[33]
**230**	3α,21β,25-trioltirucalla-21,24-epoxy-23-one	*Amoora dasyclada*	Twigs	CDCl_3,_MeOH	[52]
**231**	21β,25-dioltirucalla-21,24-epoxy-3,23-dione	*A. dasyclada*	Twigs	CDCl_3,_MeOH	[52]
**232**	Toosendine F	*M. delavayi* Franch	Bark	EtOAc	[82]
**233**	Toosendine G	*M. delavayi* Franch	Bark	EtOAc	[82]
**234**	24,25,26,27-tetranortirucalla-7-ene-3,21-dione	*A. dasyclada*	Twigs	EtOAc	[83]
**235**	3α,21β-dihydroxy-24,25,26,27-tetranortirucalla-7-ene-23-one	*A. dasyclada*	Twigs	EtOAc	[83]
**236**	Sapelin A	*Trichillia hispida*	Leaves	EtOH	[59]
**237**	Bourjotinoione A	*T. hispida*	Leaves	EtOH	[59]
**238**	Bourjotinolone A monoacetate	*T. hispida*	Leaves	EtOH	[59]
**239**	Toona triterpenoids A	*Toona sinensis*	Bark	MeOH	[50]
**240**	Bourjotinolone A diene	*T. hispida*	Leaves	EtOH	[59]
**241**	2-acetoxy-1-(α-acetoxybenzyl)naphthalene	*Trichilia hispida*	Leaves	EtOH	[33]
**242**	Sapelin B	*Munronia delavayi* Franch	Bark	EtOAc	[82]
**243**	Hispidone diacetate	*T. hispida*	Leaves	EtOH	[59]
**244**	Sapelin B acetonide	*T. hispida*	Leaves	EtOH	[59]
**245**	Hispidone acetonide	*T. hispida*	Leaves	EtOH	[59]
**246**	Munronoside I	*Munronia delavayi*	Leaves	EtOH	[64]
**247**	Munronoside II	*M. delavayi*	Leaves	EtOH	[64]
**248**	Munronoside III	*M. delavayi*	Leaves	EtOH	[64]
**249**	Munronoside IV	*M. delavayi*	Leaves	EtOH	[64]
**250**	2-acetoxy-1-(α-acetoxybenzyl)naphthalene	*Trichilia hispida*	Leaves	EtOH	[33]
**251**	1,2-diacetoxynaphthalene	*T. hispida*	Leaves	EtOH	[33]
**252**	(+) 21*R*,23*R*-epoxy-21α,25-dimethoxyapotirucall-7-en-3,24 dione	*Dysoxylum binectariferum*	Stem bark	EtOH	[71]
**253**	Dysolenticin F	*D. lenticellatum*	Twig and leaves	EtOH	[42]
**254**	25-epoxytirucall-7-ene-3,23-dione	*D. lenticellatum*	Twig and leaves	EtOH	[101]
**255**	Dysolenticin H	*D. lenticellatum*	Twig and leaves	EtOH	[101]
**256**	Dysolenticin I	*D. lenticellatum*	Twig and leaves	EtOH	[101]
**257**	(3*R*,5*R*,9*R*,10*R*,13*S*,14*S*,17*S*)-17-{(2*R*,3*S*,5*R*)-5-[(2*S*)-3,3-dimethyloxiran-2-yl]-2,5-dimethoxytetrahydrofuran-3 yl}-2, 3, 4, 5, 6, 9, 10, 11, 12, 13, 14,15,16,17-tetradecahydro-4,4,10,13,14-pentamethyl-1H-cyclopenta[a]phenanthren-3-ol	*Aphanamixis grandifolia*	Stem bark	EtOH	[73]
**258**	(5*R*,9*R*,10*R*,13*S*,14*S*,17*S*)-17-{(2*R*,3*S*,5*R*)-5-[(2*S*)-3,3-dimethyloxiran-2-yl]-2,5-dimethoxytetrahydrofuran-3-yl}-1, 2, 4, 5, 6, 9, 10, 11, 12, 13, 14, 15, 16, 17-tetradecahydro-4,4,10,13,14-pentamethyl-3H-cyclopenta[a]phenanthren-3-one	*A. grandifolia*	Stem bark	EtOH	[73]
**259**	(3*R*,5*R*,9*R*,10*S*,13*S*,14*R*,17*S*)-3-oxo-10,13-dimethyl-2,3,4,5,6,7,8,9,11,12,14,15,16,17-tetradecahydro-1H-cyclopenta[a]phenanthrene-17-carboxylate	*A. grandifolia*	Stem bark	EtOH	[40]
**260**	3β-hydroxy-urs-12-en-28-oic acid 17β-[4,4-dimethoxy-2,2-dimethyl-tetrahydrofuran-3-yl] ester	*A. grandifolia*	Stem bark	EtOH	[40]
**261**	3-oxo-urs-12-en-28-oic acid-17β-[4,4-dimethoxy-5,5-dimethyl-γ-lactone]	*A. grandifolia*	Stem bark	EtOH	[40]
**262**	21α-acetylmelianone	*Swietenia humilis Zucc*	Seeds	EtOAc	[43]
**263**	Melianone	*S. humilis Zucc*	Seeds	EtOAc	[43]
**264**	Dysoxyhaine C	*Dysoxylum hainanense*	Stem bark	EtOH/H_2_O	[76]
**265**	Nilocetin	*Cedrela fissilis*	Roots		[25]
**266**	Dyvariabilin H	*Dysoxylum variabile*	Stem bark	MeOH	[28]
**267**	Altissimanin A	*Cedrela odorata*	Leaves and twigs	EtOH	[25]
**268**	Dymacrin H	*Dysoxylum macranthum*	Stem bark	EtOH	[26]
**269**	Dymacrin I	*D. macranthum*	Stem bark	EtOH	[26]
**270**	Dymacrin J	*D. macranthum*	Stem bark	EtOH	[26]
**271**	3-β-tigloylmelianol	*M. azedarach*	Fruits	MeOH	[27]
**272**	21-β-acetoxymelianone	*M. azedarach*	Fruits	MeOH	[27]
**273**	Dyvariabilin E	*Dysoxylum variabile*	Stem bark	MeOH	[28]
**274**	Dyvariabilin F	*D. variabile*	Stem bark	MeOH	[28]
**275**	Dyvariabilin G	*D. variabile*	Stem bark	MeOH	[28]
**276**	Niloticin	*D. variabile*	Stem bark	MeOH	[28]
**277**	24,25-epoxy-3β,23-dihydroxy-7-tirucallene	*D. variabile*	Stem bark	EtOAc	[23]
**278**	Melianone (24,25-epoxyflindissone, cneorin NP37)	*Chisocheton paniculatus*	Root wood	EtOH	[49]
**279**	3-*O-*acetyl-21*R*-O-methyltoosendanpentol	*Toona sinensis*	Bark	MeOH	[50]
**280**	3α-hydroxy-24, 25, 26, 27-tetranortirucall-7-ene-23(21)-lactone	*A. dasyclada*	Stems	CDCl_3,_MeOH	[52]
**281**	3α-acetoxy-21β-methoxy-24,25,26,27-tetranortirucall-7-ene-23(21)-lactone	*Aphanamixis grandifolia*	Stem bark	EtOH	[99]
**282**	3β,16-dihydroxy-25-hydroperoxytirucalla-7,23(24)-dien-6-oxo acid	*Melia toosendan*	Stem bark	EtOH	[100]
**283**	3β,16β-hydroxytirucalla-7,24(25)-dien-21,23-olide	*M. toosendan*	Stem bark	EtOH	[100]
**284**	3β,16β-hydroxytirucalla-7,24(25)-dien-6-oxo-21,23-olide	*M. toosendan*	Stem bark	EtOH	[100]
**285**	Toosendine A	*M. delavayi* Franch	Bark	EtOAc	[82]
**286**	Toosendine B	*M. delavayi* Franch	Bark	EtOAc	[82]
**287**	Toosendine C	*M. delavayi* Franch	Bark	EtOAc	[82]
**288**	Toosendine D	*M. delavayi* Franch	Bark	EtOAc	[82]
**289**	3α,21β,25-triol-tirucalla-21,24-epoxy-23-one	*Amoora dasyclada*	Twigs	EtOAc	[83]
**290**	21β,25-diol-tirucalla-21,24-epoxy-3,23-dione	*A. dasyclada*	Twigs	EtOAc	[83]
**291**	3α-acetoxy-24,25,26,27-tetranortirucalla-7-ene-23(21)-lactone	*A. dasyclada*	Twigs	EtOAc	[83]
**292**	Toosendanone A	*Melia toosendan*	Bark	EtOH	[66]
**293**	Odoratone	*T. Ciliatawas*	Bark	MeOH	[54]
**294**	Melianol diacetate	*Melia azedarach* L	Fruits	CHC1_3_	[70]
**295**	Melianone acetate	*M. azedarach* L	Fruits	CHC1_3_	[70]
**296**	Melianone lactone	*M. azedarach* L	Fruits	CHC1_3_	[70]
**297**	Melianone epoxy-triol	*M. azedarach* L	Fruits	CHC1_3_	[70]
**298**	Melianone tetraol	*M. azedarach* L	Fruits	CHC1_3_	[70]
**299**	Melianone 7,9(11)-heteroannular diene	*M. azedarach* L	Fruits	CHC1_3_	[70]
**300**	Lipomelianol	*Melia Toosendan*	Fruit	MeOH	[102]
**301**	Melianone γ-lactone	*M. azedarach* L	Bark	C_6_H_14_	[70]
**302**	Melianol	*M. azedarach* L	Bark	C_6_H_14_	[70]
**303**	Melianol (C_21_-α-OH epimer)	*M. azedarach* L	Bark	C_6_H_14_	[70]
**304**	Melianone turraenthin acetate	*M. azedarach* L	Bark	C_6_H_14_	[70]
**305**	24-epi-melianodiol	*Aglaia andamanica*	Leaves	MeOH	[58]
**306**	Dysoxylumstatin B	*Dysoxylum lukii*	Stem barks	EtOH	[59]
**307**	Toonapubesin A	*Toona ciliata* var *pubescens*	Stem bark	MeOH	[60]
**308**	Toonapubesin B	*T. ciliata* var *pubescens*	Stem bark	MeOH	[60]
**309**	Toonapubesin C	*T. ciliata* var	Stem bark	MeOH	[60]
**310**	22*S*,23*S*,24*S*,25-diepoxy-tirucalla-7-en-3-one	*pubescens*	Stem bark	MeOH	[60]
**311**	24-methoxy-25-hydroxy-tirucalla-7-en-3-one	*T. ciliata* var	Stem bark	MeOH	[60]
**312**	Cneorin-NP36	*pubescens*	Stem bark	MeOH	[60]
**313**	Meliasenin R	*Melia toosendan*	Fruits	EtOAc	[87]
**314**	Meliasenin Q	*M. toosendan*	Fruits	EtOAc	[87]
**315**	24,25-epoxy-3β-hydroxy-20-oxo-7-tirucallene	*M. toosendan*	Fruits	EtOAc	[87]
**316**	22,23,24,25-diepoxy-3β-hydroxy-7-tirucallene	*M. toosendan*	Fruits	EtOAc	[87]
**317**	(24*S*)-7α,8α-epoxy-24-hydroxy-21α,25-dimetoxy-19(10 → 9β)*abeo*-tirucallane-5(10)-en-3-on	*Chukrasia tabularis*	Fruits	EtOAc	[42]
**318**	(24*R*)-7α,8α-epoxy-24-hydroxy-21α-metoxy-19(10 → 9β)*abeo*-tirucallane-5(10),25-dien-3-on	*C. tabularis*	Fruits	EtOAc	[42]

#### Acyclic tirucallane-type

Acyclic side-chain tirucallane derivatives represent the largest subclass in intact tirucallane-type triterpenoids, characterized by an open, non-cyclized C-17 side chain, typically experiencing oxidation, acetylation, or esterification, while retaining the fundamental 6/6/6/5 tetracyclic tirucallane skeleton. This subclass is structurally distinguished from cyclic-modified groups by the absence of terminal ring closure comprising C-20 to C-24 or C-25 carbon extensions (Table 2).

**Table 2:** Degraded tirucallane compounds from Meliaceae family

No	Compounds	Species	Part	Extract	References
**319**	Bourjotone	*Trichilia hispida*	Leaves	EtOH	[33]
**320**	Dysolenticin G	*Dysoxylum lenticellatum*	Twigs and leaves	EtOH	[101]
**321**	3α-hydroxy-21α-methoxy-24,25,26,27-tetranortirucall-7-ene 23(21)-lactone	*Aphanamixis grandifolia*	Stem bark	EtOH	[53]
**322**	3-oxo-urs-12-en-28-oic acid 17β-(2-oxotetrahydrofuran-3-yl) ester	*A. grandifolia*	Stem bark	EtOH	[53]
**323**	3β-hydroxy-urs-12-en-28-oic acid 17β-(2-hydroxy-2-oxotetrahydrofuran-3-yl) ester	*A. grandifolia*	Stem bark	EtOH	[53]
**324**	3-oxo-urs-12-en-28-oic acid 17β-(3-methoxy-2-oxotetrahydrofuran-3-yl) ester	*A. grandifolia*	Stem bark	EtOH	[53]
**325**	3β,19-dihydroxy-urs-12-en-28-oic acid-23-al	*A. grandifolia*	Stem bark	EtOH	[53]
**326**	3β-hydroxy-urs-12-en-28,23-olide	*A. grandifolia*	Stem bark	EtOH	[53]
**327**	3-oxo-24,25,26,27-tetranortirucall-7-ene-23(21)-lactone	*Dysoxylum laxiracemosum*	Bark	MeOH	[92]
**328**	3-hydroxy-24,25,26,27-tetranortirucall-7-ene-23(21)-lactone	*D. laxiracemosum*	Bark	MeOH	[92]
**329**	(20*S*)-3-oxo-tirucalla-25-nor-7-en-24-oic acid	*E. congoënse*	Stem bark	CH_2_Cl_2_/MeOH	[39]
**330**	4,4,14-trimethyl-3-oxo-24-nor-5α,13α,14β,17α,20*S*-chol-7-en-23-oic acid	*Toona sinensis*	Stem bark	EtOH	[38]
**331**	Methyl 3-oxotirucalla-7,24-dien-21-oate	*Aphanamixis grandifolia*	Stem bark	EtOH	[40]

Representative acyclic tirucallane-type triterpenoids include piscidinol A (**1**) from the roots of *Entandrophragma congoënse* [32], hispidols A–B (**2–3**) from leaves of *Trichilia hispida* [33], bourjotinolone C (**4**) from the same species, and sapelin F, along with the tetra-acetyl derivatives (**5–6**) [33]. Hispidols A (**2**) and B (**3**), bourjotinolone C (**4**), and sapelin F, along with the tetraacetates (**5–6**), are tirucallane-type triterpenoids isolated from *Trichilia hispida*. NMR analyses confirmed a common carbon skeleton and closely related side-chain hydroxylation at C-23–C-25, with spectral comparisons between the alcohols and the acetates supporting the assigned side-chain configurations. Hispidol A and B differ only at C-3, occurring as C-3 epimers, with an axial hydroxyl group in hispidol A and an equatorial hydroxyl group in hispidol B, indicated by characteristic ^13^C NMR chemical-shift differences [33].

Another significant compound, 3*S*,24*S*,25-trihydroxytirucall-7-ene (**7**), was isolated from the twigs and leaves of *Dysoxylum lenticellatum* [33]. Aphanamgrandin K (**8**) was obtained from stems of *Aphanamixis grandifolia*, through EtOH extraction, along with (23*Z*)-25-hydroxy-tirucalla-7,23-dien-3-one (**9**) and 3β,25-dihydroxy-tirucalla-7,23-diene (**10**) [34]. Leucophyllone (**11**) was also reported from the same source. Compound **9** is distinguished by the presence of a carbonyl group at C-3, which allowed unequivocal structural determination. Biogenetically, C-3 keto functionality is consistent with earlier proposals indicating that A-ring cleavage at C-2–C-3 requires oxygen substituents at both carbons, while cleavage at C-3–C-4 can occur with only a C-3 carbonyl group present [23, 35]. Indicalilacol D (**12**) was isolated from the fruit of *Azadirachta indica* using MeOH extraction, the positions of the disubstituted and trisubstituted double bonds were assigned to C-23 (24) and C-7 (8), respectively. Furthermore, the linkage between C-16 and C-21 was established as an ester bond based on the characteristic chemical shifts of H-16 and C-21 [36]. Two compounds 3,23-dioxotirucalla-7,24-dien-21-al (**13**) and entandrolide (**14**), were reported from *Entandrophragma angolese* leaves and seeds, respectively [37]. Compound **13** was identified as a tetracyclic tirucallane-type triterpene based on ^13^C NMR data showing two trisubstituted double bonds, one ketone, and one aldehyde carbonyl, accounting for four rings. The presence of seven methyls, eight methylenes, and four methines further supported this classification. ^1^H and 2D NMR (COSY, NOESY, HMBC) analyses showed an isopropylidene ketone fragment and a connected side-chain system, with key correlations confirming their connectivity. The partial structures closely resemble those of dymacrin D, with the main structural difference being the replacement of a methyl group at C-20 by an aldehyde [26].

Congensin B (**15**), 3,23-dioxotirucalla-7,24-dien-21-acid (**16**), and bourjotinolone B (**17**) isolated from *Toona sinensis* stem bark [38, 39], further underscore the diversity of acyclic tirucallane triterpenoids across Meliaceae species. Furthermore, methyl 3β-hydroxy-tirucalla-7,24-dien-21-oate (**18**), methyl (3β,5β,9β)-3,5-dihydroxytirucalla-7,24-dien-21-oate (**19**), (3*R*,5*R*,9*R*,10*S*,13*S*,14*R*,17*S*)-9-hydroxy-10,13-dimethyl-3-oxo-tetradecahydro-1H-cyclopenta[a]phenanthrene-17-carboxylate (**20**), 3-oxo-urs-12-en-28-oic acid 17β-*O*-[3-(1-ethoxy-1-hydroxy-2-methyl)propionyl] ester (**21**), 3-oxo-urs-12-en-28-oic acid 17β-*O*-[3-(hydroxy-2-methylpropanoyl)oxy]propyl ether (dimethoxy substituted) (**22**), 3-oxo-urs-12-en-28-oic acid 17β-*O*-[2-(1-hydroxy-2-methylpropanoyl)oxy]propyl ester (**23**), and 3-oxo-urs-12-en-28-oic acid 17β-*O*-[2-(1-hydroxy-2-methylpropoxy)propionyl] ester (**24**) [40]. In tirucallane derivatives **18–19**, the presence of a 3β-hydroxyl group enhances biological activity, while further hydroxylation at C-5 and C-9 increases polarity, which may strengthen interactions with biological targets but potentially limit membrane permeability. The C-21 methyl ester is probably important for improving structural stability and bioavailability. In contrast, compound **20**, featuring a truncated cyclopenta[a]phenanthrene core, emphasizes the importance of preserving an intact triterpene skeleton for optimal activity. For the ursane-type compounds (**21–24**), the conserved 3-oxo-urs-12-ene framework is critical, while variations in C-17 ester or ether side chains modulate activity through changes in polarity, hydrogen-bonding capacity, and steric properties [40].

Acyclic tirucallane-type triterpenoids, characterized by diverse C-17 side-chain structures, including seven tirucallane derivatives, are widely distributed among members of Meliaceae family and show considerable structural and biosynthetic diversity. From *Xylocarpus granatum*, and *X. moluccensis,* xylocarpols A–C (**25–27**) and agallochols A–D (**28–31**) were isolated from seeds, stems, and twigs using ethanol extraction [41]. Compound **25** differs in the stereochemical orientations of three chiral centers on C-17 side chain (C-22, C-23, and C-24), while compound **26** is structurally related to aphagranin D but lacks the 24-OH and 25-OH groups [42]. In the case of agallochols B and C (**29–30**), the absolute configurations, particularly of the tetracyclic tirucallane core, were confirmed to be identical to that of agallochol A (**28**), except for the absence of a chiral center at C-24 [41]. Dysoxyhaine D (**32**) was obtained from the stem bark of *Dysoxylum hainanense* using CHCl_3_ [43]. Multiple tirucallane analogues have also been identified from the genus *Cedrela*. For example, pentaol (**33**) and 3-oxo-threo-23,24,25-trihydroxy-tirucall-7-ene (**34**) were isolated from the heartwood of *C. glaziovii, C. odorata,* and *C. fissilis* through ethanolic extraction. The relative configuration of the 23,24-diol moiety was determined by the mechanism of acetonide formation [25]. Meanwhile, piscidinal A (**36**) and 25-methoxyhispidol (**38**) were extracted from leaves and twigs of *C. odorata* [25]. The spectroscopic differences between piscidinal A (**36**) and 25-methoxyhispidol (**38**) primarily arise from *O*-methoxylation at C-25 in compound **38**. This is reflected by the presence of a distinct methoxy singlet in ^1^H-NMR spectrum and a corresponding *O*–CH_3_ carbon resonance in ^13^C NMR spectrum, accompanied by a downfield shift of C-25. In contrast, piscidinal A (**36**) lacks these features and shows signals characteristic of a free hydroxyl group [25]. Toonaciliatin L (**35**) was also obtained from leaves and twigs of *Toona ciliata* var. ciliata [24]. While piscidinol B (**37**) was isolated from the stem bark of *T. ciliata* var. *henryi* [25].

A particularly rich source of acyclic tirucallanes is *Dysoxylum macranthum*, from which dysomollide A (**39**) and seven structurally related dymacrin derivatives including A (**40**), B (**41**), C (**42**), D (**43**), E (**44**), F (**45**), and G (**46**) were isolated exclusively from stem bark using EtOH extraction [26]. Each of these compounds represents sequential oxidative and hydroxylation variations at C-3, C-21, C-23, or C-25, illustrating a micro-diversification pathway specific to *Dysoxylum* species extraction [26]. Methyl kulonate (**47**) was reported from the fruits of *Melia azedarach* [27], while dyvariabilins A (**48**) and a series of related metabolites including tirucalla-7,24-diene-3β,23-diol (**49**), tirucalla-7,23,25-triene-3,6-dione (**50**), and tirucalla-7,23,25-trien-3β-ol (**51**) were isolated from the stem bark of *Dysoxylum variabile* using MeOH [28]. Under mildly acidic conditions, dyvariabilin A (**48**) and tirucalla-7,24-diene-3β,23-diol (**49**) showed significant instability. Compounds **48** and **49**, which possess an allylic hydroxyl group in the side chain, experienced complete elimination of the C-23 hydroxyl group, leading to the formation of a conjugated 23,25-diene system [28]. Furthermore, three rearranged or epoxy-modified tirucallanes 2α-ethoxy-2,3-*seco-*tirucalla-2,29-epoxy-7-ene-23-oxo-3-oic acid (**52**), (23*E*)-2α-hydroxy-tirucall-7,23,25-triene-3-one (**53**), and 2,3-*seco-*tirucalla-2,3;2,29-diepoxy-7-ene-3,23-dione (**54**) were obtained from leaves and roots of *Aphanamixis grandifolia* through EtOH extraction [31]. These compounds showed significant structural diversification compared to intact tirucallanes, including C-2/C-3 *seco* cleavage, epoxide formation at C-2/C-29, and varying degrees of oxidation at C-3 and C-23 [28]. In the genus *Aglaia*, 25-methoxy-tirucall-7,23(trans)-diene-3-one (**55**) was isolated from the stem bark of *A. leucophylla* [44]. ^13^C NMR signals corresponding to C-8 and C-7 appeared at δ_C_ 145.8 and 117.8 ppm. These chemical shift values are characteristic of the Δ^7−^tirucallane and Δ^7−^euphane frameworks [44].

Additional acyclic derivatives were identified from *Dysoxylum hainanense*, including 3β,22*S*-dihydroxy-tirucalla-7,24-dien-23-one (**69**), 23,26-dihydroxy-tirucalla-7,24-dien-3-one (**57**), and 3β,23-dihydroxy-tirucalla-7,24-diene (**58**), all isolated from stem bark using MeOH–H_2_O extraction [23]. From *Trichilia maynasiana*, two metabolites type were obtained, namely 3β,24-dihydroxy-tirucalla-7,25-diene-24-sulfate (**59**) and a mixture of 21-oxo-melianodiol, 21-oxo-meliantriol, and toosendanic acids A (**60**) using a solvent combination of *n*-hexane, CHCl_3_, and MeOH [45]. Sulfation at C-24 in 3β,24-dihydroxy-tirucalla-7,25-diene-24-sulfate (**59**) enhances molecular polarity and may affect biological activity. Similarly, side-chain oxidation in 21-oxo-melianodiol, 21-oxo-meliantriol, and toosendanic acids A indicates that the extent of oxidation is a key factor in regulating activity by altering polarity and molecular interactions [45]. Fruit extracts of *Melia azedarach* yielded meliastatin (**61**) [27, 46], while two mesendanin derivatives, 3-epimesendanin S 12-acetate (**62**) and 3-epimesendanin S (**63**) were isolated from the roots of *Walsura trichostemon* using EtOAc and MeOH extraction [47]. Compound **62** indicates that the presence of a hydroxyl group at C-12 may play a crucial role in enhancing biological activity [47].

Other acyclic tirucallane congeners include meliasenin G (**64**) from the stem bark of *Melia toosendan* [48], chisopanin M (**65**) from twigs of *Chisocheton paniculatus*, as well as phellochin (**66**) from *Chisocheton cumingianus* subsp. *balansae*, both obtained using EtOH extraction [49]. Further acyclic side-chain tirucallane-type triterpenoids include 21α-methylmeliandiol (**70**), isolated from the bark of *Toona sinensis* using MeOH extraction. **67** represents another oxygenated diol-type derivative reported from the species [50]. From the stem bark of *Chisocheton* species, chisopaten A (**68**) and chisocarpene A (**69**) were obtained from *C. patens* and *C. lasiocarpus* [14], respectively, while 24,25-epoxy-tirucall-7-ene-3,23-dione (**70**) was isolated from the stems of *Amoora dasyclada* using MeOH, further illustrating the diversity of hydroxyl- and carbonyl-substituted acyclic congeners in the genera [14]. Successive studies on *Aphanamixis grandifolia* and *Amoora tetrapetala* reported structurally modified acyclic tirucallanes, including 22*E*-hydroxytirucalla-7,24-dien-3,3-dione (**71**) from *A. tetrapetala* branches [51], 3α,24β,25-trihydroxy-21,21-dimethoxy-23-oxotirucall-7-ene (**72**) from *A. grandifolia* stem barks [52], and (23*E*)-2α-hydroxytirucalla 7,23,25-triene-3-one (**73**) from leaves and twigs [53]. These compounds show related functional-group modifications on tirucallane skeleton, with variations in oxidation, hydroxylation, and methoxylation at C-2, C-3, C-21 [53]. Additionally, toonaciliatavarin E (**74**) was isolated from the bark of *Toona ciliatawas*, representing a less oxygenated acyclic derivative in the compound class [54].

Non-oxygenated representatives of acyclic side-chain tirucallane subgroup include ( −)-leucophyllone (**75**), isolated from the bark of *Aglaia leucophylla* using MeOH extraction [55]. Additional related compounds were also obtained from the same C₆H₁₄ bark extract. These include 16-hydroxybutyrospermol (**76**), eupho-7,26-dien-3β,16β-diol diacetate (**77**), eupha-7-en-3β,16β-diol (**78**), 16α,21-dihydroxyeupha-7,24-dien-3-one diacetate (**79**), 16β-hydroxyeupha-7,24-dien-3-one (**80**), butyrospermol (**81**), 16-epikulinone (**82**), butyrospermone (**83**), 16α,21-dihydroxyeupha-7,24-dien-3-one (**84**), 16α,21-dihydroxyeupha-7,24-dien-3-one ethylene ketal (**85**), and 21-hydroxyeupha-7,24-dien-3,16-dione 3-ketal (**86**) [56]. The compounds reported expand the range of structures representing possible oxidative intermediates in which a precursor such as butyrospermol is hydroxylated at C-16 to form kulinone, followed by oxidative rearrangement, possibly through a 7,8-epoxide. The presence of kulactone and methyl kulonate suggests that side-chain oxygenation may occur before ring rearrangement. Collectively, these metabolites demonstrate the remarkable biosynthetic capacity of *M. azedarach* to generate hydroxylated, carbonyl-modified, semi-reduced, esterified, and ketal-stabilized acyclic tirucallane derivatives [37].

Additional acyclic tirucallane-type compounds from *M. azedarach* include kulonic acid (**87**), an eupha-7,24-diene (**88**), and methyl 3-oxotirucalla-8,24-dien-21-oate (**89**), all isolated from the bark using C₆H₁₄ [37]. In *Walsura piscidia*, several modified acyclic tirucallanes were isolated from leaf extracts, including acetyl-piscidinol A (**90**), 7,9(11)-tirucalladiene (euphadiene-type) (**91**), piscidinol A acetonide (**92**), an additional acetonide analogue (**93**), lupenone aldehyde (**94**), and a highly oxygenated pregnane-type metabolite, 7α,12α,21-triacetoxy-4,4,8,10,13-pentamethyl-17-(2,2-dimethylpropionyloxy)-pregnane (**95**) [57]. These structures show the extensive oxidative tailoring and acetalization reactions possible in the genus. The product was identified by a characteristic aldehydic proton resonance at δ_H_ 9.75 (t), and the observed chemical conversions corroborate the structural classification of compounds **90–92**. The configuration at C-20 was tentatively proposed as *S* on biogenetic grounds, given the widespread occurrence of tirucallane derivatives in Meliaceae family, while euphanes with a 20*R* configuration are mainly restricted to *Melia* species [57].

Three further acyclic congeners, 24-epi-piscidinol A (**96**), aglaiodiol (**97**), and moluccensin P (**98**), along with moluccensin Q (**99**), were reported from MeOH extract of *Aglaia andamanica* leaves, indicating strong chemotaxonomic linkage to *Aphanamixis* through similar oxygenation patterns [58]. EtOAc extracts of *Dysoxylum lukii* stem bark produced four additional tirucallane analogues, namely 3β-hydroxytirucalla-7,24-diene-6,23-dione (**100**), 3β-hydroxytirucalla-7,24-dien-23-one **(101**), 3β,26-dihydroxytirucalla-7,24-diene-6,23-dione (**102**), and methyl 6-oxomasticadienolate (**103**) [59], reflecting regioselective hydroxylation and carbonyl formation primarily at C-3, C-6, C-23, and C-26.

A group of structurally distinct compounds, toonapubesins D–G **(104–107)**, along with dihydroniloticin **(108)**, methyl-24,25,26,27-tetranortirucall-7-en-3-oxo-23-oate **(109)**, and 24*S*,25-dihydroxytirucall-7-en-3-one **(110)** were isolated from the stem bark of *Toona ciliata* var. *pubescens* through MeOH extraction, demonstrating the co-occurrence of nor-tirucallane and stereochemically enriched intermediates in the species [60]. Meliasenin E **(111)** was reported from the fruits of *Melia azedarach* [61], while *Melia toosendan* produced meliasenin A **(112)** and H **(113)** from the stem bark through EtOH extraction [48]. Further fruit-derived metabolites from *Chukrasia tabularis* include hispidol C **(114)** and tirucalla-7,24-dien-3β-ol **(115)**, representing a unique combination of oxidative side-chain cleavage and partial cyclic rearrangement in tirucallane skeleton [42]. Extensive oxidative diversification was also observed in *M. toosendan* fruits, where MeOH extraction yielded (3*S*,5*R*,9*R*,10*R*,13*S*,14*S*,16*S*,17*S*,20*S*)-3,16,21-trihydroxy-tirucalla-7,24-diene **(116)**, (3*S*,5*R*,9*R*,10*R*,12*R*,13*S*,14*R*,16*S*,17*S*,20*S*)-3,12-dihydroxy-tirucallane-7,24-diene-21-methyl ester **(117)**, and toosendine E **(118)** [62].

From bark of *Entandrophragma angolense* produced entandrophin C **(211)** and entanolide **(119)** using *n*-hexane/EtOAc extraction [16]. *Aphanamixis*-derived *seco*-tirucallane analogue, 2α-ethoxy-2,3-*seco*-tirucalla-2,29-epoxy-7-ene-23-oxo-3-oic acid **(120)**, was also reported from the leaves and twigs of *Aphanamixis grandifolia*, representing one of the most structurally rearranged members in the subclass [53]. In addition, tirucallane-related metabolites, including 4,4,14-trimethyl-3-oxo-24-nor-5α,13α,14β,17α,20S-chol-7-en-23-oic acid (**121**), toonapubesin B (**122**), and 3β,22S-dihydroxy-tirucalla-7,24-dien-23-one (**123**), were reported from the stem bark of *Dysoxylum gaudichaudianum* using *n*-hexane extraction [63]. The discovery and isolation of several novel and structurally diverse triterpenoid compounds, specifically derivatives of tirucallane and *seco*-tirucallane, from the bark and foliage extracts of *Entandrophragma angolense, Aphanamixis grandifolia,* and *Dysoxylum gaudichaudianum* underscore the significant chemical complexity and rich source of secondary metabolites in these plants, exemplified by the highly rearranged *seco*-tirucallane analogue. (3β)-22,23-epoxytirucall-7-ene-3,24,25-triol (**124**) originated from EtOH extract of Munronia delavayi leaves [64]. 3-oxo-24,25,26,27-tetranortirucall-7-ene-23(21)-lactone (**125**) were isolated from the twigs and stems of Amoora dasyclada using CHCl3/MeOH extraction [52]. The presence of a 23(21)-lactone ring in the tetranor derivatives **125**, can be rationalized through sequential oxidative transformations of the side chain while retaining the original chiral centres of tirucallane core. ROESY supported the assignment of (*S*)-configuration at C-20 [52]. Trichostemonoate (**126**) was isolated from the stem bark of *Walsura trichostemon*, through EtOAc extraction [65]. Toosendanone acid A (**127**), and B (**128**), all isolated through EtOH extraction [66]. Typical protolimonoids bearing a tetrahydrofuran ring at C-17, compound **126** features an unprecedented cyclopentanyl moiety at this position. The relative configuration of toosendanone Acid A (**127**) was determined by ROESY analysis, where correlations of H-3/CH_3_-28 and H-16/CH_3_-18 indicate that H-3 and H-16 are β-oriented. The splitting pattern of H-3 further supports a β-oriented hydroxyl at C-3. Comparison of NMR data for toosendanone acid B **128** and **127** showed identical structures except for the configuration at C-3, with NMR signals characteristic of an α-oriented hydroxyl, leading to the assignment of **128** as C-3 epimer of **266** [66]. Furthermore, the bark of *Toona ciliata* yielded toonamicrocarpavarin (**129**), and phellocin (**130**) through MeOH extraction [54]. The relative configuration of toonamicrocarpavarin (**129**) was shown to be identical to that of piscidinol A based on diagnostic NOE interactions, including correlations of H-5/H-9, H-9/H-18, H-18/H-20, and H-30/H-17, along with the absence of NOE cross-peaks between H-18/H-17, H-18/H-30, and H-9/H-19 [54]. An oxygenated tirucallane analogue, 3β-hydroxy-3-decarbonyl-24-epi-piscidinol A (**132**), was obtained from the seeds of *Xylocarpus moluccensis*, through EtOH extraction [67]. Trichostemonol (**131**) was obtained from the twigs using CH_2_Cl_2_ of *Walsura trichostemon*, [68]. Key correlations for (**132),** including CH_3_-28/CH_3_-19, CH_3_-19/CH_3_-30, and CH_3_-30/H-17, indicated β-orientation of the tetracyclic core, while correlations of H-3 with H-1β, H-2β, and CH₃-28 supported the α-configuration. The side-chain configuration at C-17 was assigned based on close NMR similarity to 3-epimesendanin S [69].

Fruits of *Melia azedarach* produced a chemically diverse set of melianol-type triterpenoids, melianol tetraol diene (**133**), and melianol bromoacetal (**134**) isolated through CHCl_3_ extraction [70]. The proposed acetal configuration in melianone is rationalized by the relative orientation of substituents in the pyran system. The formation of C(21)–C(24) ether bridge is facilitated by nucleophilic attack of C-24 oxygen on the cationic C-21 center, with stabilization provided by a neighboring oxygen atom. Conformational analysis requires an *R* configuration at C-24, as a dihedral angle of approximately 90° between H-23 and H-24 allows the substituents to adopt an orientation favourable for bromoacetal formation. In line with the stereochemical pattern, melianol bromoacetal (**134**) can be correlated as members of a common biosynthetic lineage retaining conserved *R* configurations at the side-chain stereocenters, despite extensive oxidative and cyclization modifications [70]. Extensive investigation of *Melia azedarach* bark has led to the discovery of numerous euphane-based congeners, including kulinone (**135**), kulinone acetate (**136**), 7,9(11)-heteroannular diene euphane-type (**137**), butyrospermol (**138**), dihydrobutyrospermol acetate (**139**), euphenol (**140**), 16-dehydrokulinone (**141**), and a series of functionalized derivatives (**142–150**) which were mainly from hexane fractions [37, 70]. Kulinone derivatives, butyrospermol-related compounds, and melianol-type triterpenoids share a common euphane/tirucallane carbon framework, indicating conservation of the relative stereochemistry at the ring junctions and side-chain chiral centers. Variations in configuration are mainly confined to specific positions, such as C-21 epimerization in melianol analogues, while the fundamental stereochemical architecture remains unchanged, enabling extensive functional diversification in this species [37, 70].

#### Cyclic side-chain 4C derivatives

Cyclic side-chain 4C derivatives consist of ten lactone-type tirucallane triterpenoids (**151–160**). The classification as cyclic side-chain 4C derivatives is attributed to oxidative truncation and cyclization of the C-17 side chain, resulting in the formation of a four-carbon lactone ring. These metabolites were exclusively isolated from the stem bark of *Melia azedarach* using *n*-hexane extraction followed by non-polar fractionation and chromatographic purification [37]. The identified compounds include kulactone (**151**), kulactone ketal (**152**), 24,25-dihydrokulactone (**153**), kulolactone (**154**), kulolactone acetate (**155**), 24,25-dihydro-3-epikulolactone (**156**), 24,25-dihydro-14d-epoxide kulactone derivative (**157**), 16-epikulactone 3-ethylene ketal (**158**), 24,25-dihydro-16-epikulactone (**159**), and 3-epi-16-epikulactone (**160**) [37]. Among these compounds, four have B, C, and D ring systems with an acetoxy group replacing the C-7 ketone. In addition, numerous C-nor and C-methyl triterpenoids representing different stages of oxidation and degradation have been reported from other members of Meliaceae family [37].

#### Cyclic side-chain 5C derivatives

Cyclic side-chain 5C derivatives (**161–214**) represent apotirucallane-type triterpenoids possessing five-carbon side-chain cyclization formed through advanced oxidative and epoxidative transformations. These compounds are classified as cyclic side-chain 5C derivatives because C-17 side chain experiences cyclization without carbon loss, forming a stable five-membered ring. This distinguishes the compounds from 4C cyclic derivatives, in which side-chain truncation reduces the ring to four carbons. The preservation of all five carbons in the side chain, combined with ring closure, justifies the inclusion in the 5C cyclic subclass. These constituents are widely distributed across multiple Meliaceae species and plant organs, primarily obtained through ethanol or methanol extraction. Four epoxy–methoxy apotirucallanes, including (**161**), (**162**), (**163**), and (**164**), were isolated from the stem bark of *Dysoxylum binectariferum* using EtOH extraction, featuring epoxide rings and methoxy groups that influence side-chain rigidity and reactivity [71]. Furthermore, the cortex of *Cedrela sinensis* yielded entagenic acid/methyl ester (**165**), 21α-methoxy-24,25-dihydroxyapotirucall-7-en-3-one (**166**), and dysolenticins C–E (**167–169**) through MeOH extraction. These compounds contain hydroxyl, carbonyl, and methoxy functionalities that enhance polarity and hydrogen-bonding potential [72]. Three epoxylanostane derivatives (**170–172**) were isolated from the stem bark of *Aphanamixis grandifolia* using EtOH extraction [73]. From the fruits of *Azadirachta indica*, 24,25-acetonide (**173**), 3-oxo-24,25-acetonide derivative (**174**), meliasenin S (**175**), meliantriol (**176**), meliasenin T (**177**), and indicalilacol C (**178**) were obtained using MeOH extraction [36]. Analysis of ^1^H-NMR and NOESY spectra showed that **178** shares the same C-17 side-chain configuration as **175**. Comparison with C-21 epimer of **152** further suggests that the stereochemistry at C-21 is a key factor influencing biological activity [36].

Methyl (3*R*,5*S*,10*S*,13*R*,14*R*,17*S*)-10,13-dimethyl-3-oxo-2,3,4,5,6,7,8,9,11,12,14,15,16,1tetradecahydro-1H cyclopenta[a]phenanthrene-17-carboxylate (**179**) was identified from *A. grandifolia* using EtOH extraction [40]. EtOAc extraction also yielded agladupol D (**180**) and E (**181**) from *Aglaia duperreana* leaves and bark [74]. Xylocarpol D (**182**) were isolated from the seeds, stems, and twigs of *Xylocarpus granatum* and *X. moluccensis* [41]. In xylocarpol D (**182**), the absolute configurations of C-3, C-23, and C-24 were assigned as *S*, and *R* respectively, establishing the structure as (3*S*,5*R*,9*R*,10*R*,13*S*,14*S*,17*S*,20*R*,22*S*,23*R*,24*S*)-3,23,24-trihydroxy-22,25-epoxytirucalla-7-ene. The relative configurations of tetracyclic tirucallane cores of agladupol D (**180**) and E (**181**) were determined through NOESY interactions, establishing the stereochemistry of rings A–D and the C-17 side chain [41]. Aerial parts of *Guarea guidonia* produced flindssone (**183**) and picroquassin E (**184**) [69], while *Toona ciliata* yielded toonaciliatine A (**185**) using *n*-hexane and CDCl₃ [75]. Other structurally related constituents include Odoratol (**186**), 6-dehydromexicanol (**187**), isoodoratol (**188**), and 22*S*,3α-dihydrotirucalla-7,24-dien-23-one (**189**) isolated from *T. ciliata* var. *henryi*, *Cedrela odorata*, and *Cedrela fissilis* through EtOH/H_2_O extraction [76]. Meliatetraolenone (**190**), 21α-methylmelianodiol (**191**), and toonaciliatin K (**192**) were produced from *T. ciliata* var. *ciliata* leaves and twigs, using EtOH/H_2_O extraction [76]. Furthermore, EtOH-extracted triterpenoids from ripe fruits of *Melia azedarach* include 21β-ethylmelianodiol (**193**) [30]. In *Aphanamixis polystachya* fruits, polystanin C (**194**) and D (**195**) were isolated by EtOH extraction. NOE correlations defined the stereochemistry of the tetracyclic cores and C-17 side chain [77]. *T. ciliata* also produced 25-dihydroxyapotirucalla-7-en-3-one (**196**) from the leaves and twigs [78]. NMR and NOE analyses confirmed a β-oriented substituent at C-21 and an *S* configuration at C-24, establishing the structure of 21β-methylmelianodiol (**197**), which is reported for the first time from *M. azedarach* fruits [30]. Additional compounds isolated include 21β-methylmelianodiol (**197**), (21*S*,23*R*)-epoxy-21β,25-dimethoxy (**198**), 21β,25-dimethylmelianodiol (**199**), 21,25-dimethylmelianodiol (**200**), (21*R*,23*R*)-epoxy-24-hydroxy-21β-methoxytirucall-7,25-dien-3-one (**201**), methoxytirucall-7,25-dien-3-one (**202**), 21*R*,23*R*-epoxy-21β,24-dihydroxy- (**203**), and tirucall-7,25-dien-3-one (**204**) [29]. Comparative NMR analysis of compounds **194** and **197** showed that **197** differs by having an ethoxyl group instead of a methoxyl at C-21, with corresponding shifts at C-17 (δ_C_ 45.1), C-21 (δ_C_ 103.54), and C-22 (δ_C_ 31.8), confirming the β-orientation [30].

Melianodiol (**205**) and indicalilacol B (**206**) were obtained from the stem bark of *Chisocheton pentandrus*, through MeOH extraction, confirming the orientations of hydroxyl and side-chain substituents [79]. Fruits of *Melia toosendan* yielded epoxy- and trihydroxy-lanostane derivatives, including (**207–209**) through EtOH extraction, with epoxide and hydroxyl functionalities established the relative and absolute configurations of the side chains [62]. Bark extracts of *Entandrophragma angolense* yielded entandrophrin A (**210**), B (**211**), and C (**212**) isolated with *n*-hexane/EtOAc [16]. Two stereoisomeric arylacylated derivatives of synthetic product, namely 3-*O*-[(*S*)-α-methoxy-α-(trifluoromethyl)phenylacetyl] ester **(213)** and 3-*O*-[(*R*)-α-methoxy-α-(trifluoromethyl)phenylacetyl] ester **(214)**, were isolated from the root bark of *Melia volkensii*. These compounds show a CF_3_-substituted phenylacetyl group that enhances lipophilicity and may strengthen protein–ligand interactions [14].

#### Cyclic side-chain 6C

Cyclic side-chain 6C derivatives **(215–238)** represent a structurally diverse subgroup of tirucallane triterpenoids characterized by a six-carbon ring-fused side chain enriched in epoxide, ester, diene, and polyoxygenated functionalities. These characteristics contribute to conformational rigidity and broadened biological potential. 2-endo-acetoxy-1-benzyl-1,4-epoxy-1,2,3,4-tetrahydronaphthalene **(215)**, isolated from leaves of *Trichilia hispida*, features a benzyl-bridged epoxy system, indicating stereochemical variation through side-chain cyclization [71]. Compounds such as toonaciliatavarin D **(216)**, 3-episapelin A **(217**), and hispidone **(218)** from *Toona ciliata* tissues retain tirucallane tetracyclic core while incorporating multi-oxygenated 6C side chains, indicative of extensive oxidative tailoring during biosynthesis [25]. Epoxy-rich congeners, including 3α,21β,25-triol-tirucalla-21,24-epoxy-23-one **(219)**, 21β,25-diol-tirucalla-21,24-epoxy-3,23-dione (**220**), and sapelinone A **(221)** from *Amoora dasyclada* demonstrate 21,24-epoxy bridging, which stabilizes the side-chain conformation and may enhance biological specificity [29].

Further members include bourjotinoline A **(222)** and chisiamols G **(223)** from *Chisocheton paniculatus*, which show multi-hydroxyl substitution in the fused side chain [5, 30]. *T. hispida* also produced hispidone diene **(224)** and bourjotinolone A diene **(203)**, where diene conjugation possibly contributes to enhanced electrophilicity and potential cytotoxicity [80]. Furthermore, the roots of *Trichilia connaroides* provided lipo-3-episapelin A **(225**) [81]. Dysoxylumstatin A **(226**) and dubione B (**227**) from the bark of *Dysoxylum lukii* demonstrate advanced oxygenation patterns associated with rearranged cyclic side-chain frameworks [59]. Meliasenin F **(228)** from *Melia toosendan* further exemplifies oxidatively reorganized tirucallane motifs, reinforcing the diversity of this subgroup [48]. The compound 2-acetoxy-1-benzyl-naphthalene (**229**) was isolated from the leaves of *Trichilia hispida* using EtOH extraction. The configuration can be deduced from the spatial arrangement of the acetoxy and benzyl groups on the naphthalene framework. The acetoxy group at C-2 and the benzyl group at C-1 adopt positions that reduce steric clashes, thereby establishing the molecule stereochemistry [33]. Two closely related epoxide-bearing tirucallane derivatives, namely 3α,21β,25-triol-tirucalla-21,24-epoxy-23-one (**230**), 21β,25-dioltirucalla-21,24-epoxy-3,23-dione (**231**), were isolated from the twigs and stems of *Amoora dasyclada* using CHCl_3_/MeOH extraction [52]. The presence of a 21,24-epoxy moiety in compounds **230** and **231**, as well as the formation of a 23(21)-lactone ring, can be rationalized through sequential oxidative transformations of the side chain while retaining the original chiral centres of tirucallane core. ROESY supported the assignment of (*S*)-configuration at C-20 [52]. Toosendine F (**232**), and G (**233**) were isolated from the bark of *Munronia delavayi* through EtOAc extraction [82]. 24,25,26,27-tetranortirucall-7-ene-3,21-dione (**234**) and 3α,21β-dihydroxy-24,25,26,27-tetranortirucall-7-ene-23-one (**235**) were obtained from the same plant part and solvent system [83]. Compounds **234–235** represent advanced side-chain degradation products, forming tetra nortirucallane skeletons with lactone or ketone functionalities at C-21/C-23 [83]. Finally, the leaves of *Trichilia hispida* yielded a group of sapelin- and bourjot-type derivatives, including sapelin A (**236**), bourjotinoione A (**237**), bourjotinolone A monoacetate (**238**), through EtOH extraction [59].

#### Cyclic side-chain 7C

Cyclic side-chain 7C derivatives (**239–245**) constitute a structurally diverse subgroup of tirucallane triterpenoids characterized by a seven-carbon ring-fused side chain. Toona triterpenoids A **(239)** from Bark *Toona sinensis* using MeOH extract demonstrate additional acyl modifications in this class [50]. *T. hispida* also produced bourjotinolone A diene (**240**), where diene conjugation possibly contributes to enhanced electrophilicity and potential cytotoxicity [80]. Compounds 2-Acetoxy-1-(α-acetoxybenzyl)naphthalene (**241**)) were isolated from EtOH extract of Trichilia hispida leaves. NOE interactions in 2-acetoxy-1-(α-acetoxybenzyl)naphthalene also showed α-orientation of the benzyl substituent [33]. Sapelin B (**242**) was isolated from the bark of Munronia delavayi using EtOAc extraction [82]. Leaves of *Trichilia hispida* yielded a group of sapelin- and bourjot-type derivatives, including hispidone diacetate (**243**), sapelin B acetonide (**244**), and hispidone acetonide (**245**) using EtOH extraction [59].

#### Cyclic–glycoside conjugates

Beyond the categories described, four glycosylated metabolites, munronoside I (**246**), II (**247**), III (**248**), and IV (**249**), were isolated from leaves of *Munronia delavayi* through EtOH extraction [64], further expanding the structural diversity of tirucallane-type triterpenoids. The stereochemistry of these compounds is defined by the configuration of tetracyclic tirucallane core and β-oriented glycosidic linkage at the side chain. NMR and NOE analyses show that the hydroxyl groups on aglycone and glycosidic attachments adopt orientations minimizing steric hindrance. This structure ensures the stability of cyclic–glycoside conjugates and potentially influences biological activity.

### Epoxide-modified cyclic sidechain

Epoxide modification adds further chemical diversity to tirucallane derivatives reported from Meliaceae family. The incorporation of an epoxide ring, usually at positions previously occupied by double bonds, introduces conformational rigidity and establishes new stereocenters, affecting the overall stereochemistry of the molecule. This modification also generates a reactive site capable of participating in further chemical transformations, including nucleophilic attacks or ring-opening reactions. Structurally, epoxide influences the orientation of adjacent functional groups, and functionally, it has the potential to enhance or modulate the biological activity of triterpenoids. Compounds 2-Acetoxy-1-(α-acetoxybenzyl)naphthalene (**250**) and 1,2-diacetoxynaphthalene (**251**) were isolated from EtOH extract of *Trichilia hispida* leaves. ^1^H and ^13^C NMR data confirmed the positions of the acetoxy and benzyl substituents on the naphthalene core, while key COSY and HMBC correlations established the connectivity between the aromatic ring and the α-acetoxybenzyl or additional acetoxy group. NOE interactions in 2-acetoxy-1-(α-acetoxybenzyl)naphthalene also showed α-orientation of the benzyl substituent [33]. The compound ( +)-21*R*,23*R*-epoxy-21α,25-dimethoxyapotirucall-7-en-3,24-dione (**252**) was isolated from the stem bark of *Dysoxylum binectariferum* using EtOH extraction [71]. NMR analysis indicated that the structure features a methoxyl group at C-25, as supported by HMBC correlations between C-25 (δ_C_ 81.8) and H-23 (δ_H_ 5.04) as well as OMe signal (δ_H_ 3.20), along with corresponding shifts of H-26, H-27, and C-25. The relative configuration of tetracyclic core was established by NOESY correlations and optical rotation [71].

Four related metabolites, including dysolenticin F (**253**), 25-epoxytirucall-7-ene-3,23-dione (**254**), dysolenticin H (**255**), and dysolenticin I (**256**), were i56solated from the twig and leaf EtOH extract of *Dysoxylum lenticellatum* [84]. Two highly oxidized epoxide-containing tirucallane derivatives (**257–258**) were subsequently isolated from the stem bark of *Aphanamixis grandifolia* using EtOH extraction [73]. Additional congeners from the same species compound (**259**), tirucallane carboxylate (**260**), hydroxy-urs-12-en ester bearing a dimethoxytetrahydrofuran moiety, and compound (**261**), γ-lactone functionalized ursane analogue, were also identified from EtOH stem-bark extract of *A. grandifolia* [40]. These compounds are consistent with typical tirucallane and ursane frameworks. NOESY and HMBC correlations confirmed the relative orientations of key functional groups, including the lactone and tetrahydrofuran rings, as well as the side-chain double bond configurations [40].

The compound 21α-acetylmelianone (**262**) was purified from the seeds of *Swietenia humilis* through CHCl_3_ extraction [43], and melianone (**263**) was also identified from the same plant [43]. Dysoxyhaine C (**264**) was reported from the stem bark of *Dysoxylum hainanense* using EtOH/H_2_O extraction [76], and nilocetin (**265**) was obtained from the roots of *Cedrela fissilis* [25]. The compounds feature a hydroxy group at C-3, supporting previous observations who stated that the free hydroxy at C-3 and the polarity of C-28 are key structural determinants [85].

Dyvariabilin H (**266**) was isolated from EtOH extract of *Dysoxylum variabile* stem bark [28], while altissimanin A (**267**) was obtained from EtOH extract of *Cedrela odorata* leaves and twigs [25]. Dymacrin H (**268**), I (**269**), and J (**270**) were purified from the stem bark of *Dysoxylum macranthum* using EtOH extraction. HMBC and COSY correlations facilitated the assignment of substituents, including hydroxyl, carbonyl, and epoxide groups, while also defining the positions in tetracyclic core and side chains. NOESY correlations further established the relative stereochemistry, indicating that the orientation of hydroxyl and epoxide groups in the side chain may influence molecular conformation and potentially modulate biological activity [26]. Compounds 3-β-tigloylmelianol (**271**) and 21-β-acetoxymelianone (**272**) were isolated from the fruits of *Melia azedarach* using MeOH extraction [27].

A group of structurally related tirucallane derivatives from *D. variabile* stem bark, namely dyvariabilins E (**273**), F (**274**), G (**275**), and niloticin (**276**), were obtained using MeOH extraction [28]. The compound 24,25-epoxy-3β,23-dihydroxy-7-tirucallene (**277**) was isolated from the stem bark of *D. hainanense* using EtOAc extraction [23]. The previously reported 24,25-epoxyflindissone (**278**) was extracted from the root wood of *Chisocheton paniculatus* through EtOH extraction [49], confirming the occurrence across multiple Meliaceae genera. Another compound 3-*O*-acetyl-21*R*-*O*-methyltoosendanpentol (**279**), was isolated from the bark of *Toona sinensis* using MeOH extraction [50]. Additionally, 3α-hydroxy-24,25,26,27-tetranortirucall-7-ene-23(21)-lactone (**280**) was obtained from the stems of the same species under identical extraction conditions [52]. A further modified analogue, 3α-acetoxy-21β-methoxy-24,25,26,27-tetranortirucall-7-ene-23(21)-lactone (**281**), was subsequently isolated from the stem bark of *Aphanamixis grandifolia* through EtOH extraction [53, 107].

Three multi-oxygenated tirucallane derivatives, including 3β,16-dihydroxy-25-hydroperoxytirucalla-7,23(24)-dien-6-oxo acid (**282**), 3β,16β-hydroxytirucalla-7,24(25)-dien-21,23-olide (**283**), and 3β,16β-hydroxytirucalla-7,24(25)-dien-6-oxo-21,23-olide (**284**) were isolated from the stem bark of *Melia toosendan* through EtOH extraction [86]. The conserved tirucallane framework and ROESY/NOESY-supported relative configuration, correlate well with the multi-oxygenated tirucallane derivatives **282** and **284** isolated from *Melia toosendan*. In these compounds, structural diversification mainly originates from oxidative modifications at C-6, C-16, as well as the side chain, including hydroperoxyl substitution at C-25 and lactonization between C-21 and C-23, while the core stereochemistry remains unchanged. The close similarity of NMR data and NOESY correlations between compound **284** and the closely related analogues supports a shared relative configuration, with differences attributable to variations in oxidation state and side-chain unsaturation. Therefore, compounds **282–284** represent advanced oxidative derivatives in the same biosynthetic lineage of tirucallane triterpenoid [86]. Six oxygen-rich tirucallane derivatives, namely toosendine A (**285**), B (**286**), C (**287**), and D (**288**) were isolated from the bark of *Munronia delavayi* through EtOAc extraction [82]. Three additional truncated tirucallane derivatives, incuding 3α,21β,25-triol-tirucalla-21,24-epoxy-23-one (**289**), 21β,25-diol-tirucalla-21,24-epoxy-3,23-dione (**290**), and 3α-acetoxy-24,25,26,27-tetranortirucall-7-ene-23(21)-lactone (**291**), were isolated from the twigs of *Amoora dasyclada* using CHCl_3_/EtOAc extraction [83]. Compounds **289** and **290** retain the characteristic 21,24-epoxy functionality, while compounds **291** represent advanced side-chain degradation products, forming tetra nortirucallane skeletons with lactone or ketone functionalities at C-21/C-23. These structural variations are consistent with sequential oxidative cleavage and cyclization of the side chain, while the relative configurations of the remaining chiral centers are preserved [83]. Furthermore, *Melia toosendan* bark produced three structurally allied toosendanone derivatives, namely toosendanone A (**292**) isolated through EtOH extraction [66]. The absolute configuration of toosendanone A (**292**) was established by CD spectroscopy, which showed a negative cotton effect at 296 nm, consistent with a 4,4-dimethyl-5-hydrogen-10-methylcyclohexan-3-one skeleton, allowing assignment of the 5*R*,10*R* configuration. In contrast to typical protolimonoids bearing a tetrahydrofuran ring at C-17, compound **292** features an unprecedented cyclopentanyl moiety at this position [66]. Furthermore, the bark of *Toona ciliata* yielded odoratone (**293**) through MeOH extraction [54]. Meanwhile, fruits of *Melia azedarach* produced a chemically diverse set of melianol-type triterpenoids, including melianol diacetate (**294**), melianone acetate (**295**), melianone lactone (**296**), melianone epoxy-triol (**297**), melianone tetraol (**298**), melianone 7,9(11)-heteroannular melianol diene (**299**), lipomelianol (**300**), melianone γ-lactone (**301**), melianol (**302**), melianol C_21_-α-OH epimer (**303**) and melianone turraenthin acetate (**304**) isolated through CHCl_3_ and C_6_H_14_ extraction [70]. The proposed acetal configuration in melianone is rationalized by the relative orientation of substituents in the pyran system. Formation of C(21)–C(24) ether bridge is facilitated by nucleophilic attack of C-24 oxygen on the cationic C-21 center, with stabilization provided by a neighboring oxygen atom. Conformational analysis requires an *R* configuration at C-24, as a dihedral angle of approximately 90° between H-23 and H-24 allows the substituents to adopt an orientation favourable for bromoacetal formation.

From leaves of *Aglaia andamanica*, 24-epi-melianodiol (**305**) was isolated using MeOH extraction, representing an additional oxygenated tirucallane-type analogue from this species [58]. Compound **305** shares the same molecular formula (C_30_H_48_O_5_) and tirucallane-type triterpenoid framework as melianodiol based on MS, IR, and NMR analyses [79]. Furthermore, dysoxylumstatin B (**306**) was isolated from the stem bark of *Dysoxylum lukii* using EtOH extraction [59]. This investigation also led to the characterization of several new tirucallane-type protolimonoids, designated as toonapubesins A–C (**307–309**), together with related oxygenated tirucallane derivatives, including 22*S*,23*S*,24*S*,25-diepoxy-tirucalla-7-en-3-one (**310**) and 24-methoxy-25-hydroxy-tirucalla-7-en-3-one (**311**). The known compounds, protolimonoid cneorin-NP_36_ (**312**) obtained from the stem bark through MeOH extraction. Spectroscopic analysis showed that these compounds share a common tirucallane-type protolimonoid nucleus (rings A–D), consistent with previously reported tirucallane analogues from this species, with structural variations mainly arising from differences in side-chain oxidation and substitution patterns [60]. Finally, 21-epimeric tirucallane derivatives bearing an α-oriented hydroxyl group at C-3 have also been reported from other members of Meliaceae family, such as *Aglaia duperreana* [74], suggesting a conserved stereochemical feature in this lineage. In line with this observation, chemical investigation of *Melia toosendan* fruits led to the isolation of meliasenin R (**313**) and Q (**314**) through EtOAc extraction, together with two highly oxygenated tirucallane congeners, namely 24,25-epoxy-3β-hydroxy-20-oxo-7-tirucallene (**315**) and 22,23,24,25-diepoxy-3β-hydroxy-7-tirucallene (**316**) [87]. These compounds share a common tirucallane scaffold and retain the same relative configuration in rings A–D, with structural diversity arising mainly from epimerization at C-21 and varying degrees of side-chain oxidation. Two stereospecific *abeo*-epoxy tirucallane derivatives, namely (24*S*)-7α,8α-epoxy-24-hydroxy-21α,25-dimethoxy-19(10 → 9β)*abeo*-tirucall-5(10)-en-3-one (**317**) and the C-24 epimer (24*R*)-7α,8α-epoxy-24-hydroxy-21α-methoxy-19(10 → 9β)*abeo*-tirucall-5(10),25-dien-3-one (**318**), were isolated from the fruits of *Chukrasia tabularis* using EtOAc extraction [42]. Collectively, these results show that epimerization at C-21 and C-24, while preserving the core tirucallane stereochemistry, represents a recurrent configurational variation among oxygenated tirucallane derivatives in Meliaceae.

### Degraded tirucallane-type

**Fig. 10:**
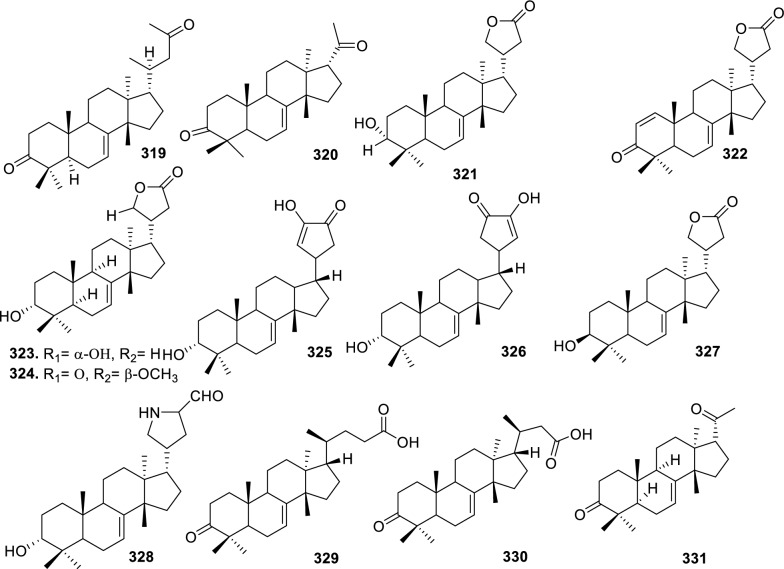
Degraded tirucallane-type** 319–331**

Degraded tirucallane-type arises as catabolic products generated by the stepwise degradation of parent frameworks (Table 2, Fig. 10). Although numerous reviews have addressed the chemistry, biological properties, and pharmacological significance of tirucallane-derived natural products, a dedicated and comprehensive assessment of the structural diversity and bioactivities remains scarce [88–93]. A series of structurally degraded tirucallane derivatives (**319–331**) has been characterized from several members of Meliaceae family, predominantly in the genera *Aphanamixis* and *Dysoxylum*. Bourjotone (**319**) was isolated from leaves of *Trichilia hispida* using EtOH extraction [33]. The transition from an equatorial to an axial hydroxyl orientation is supported by diagnostic ^13^C chemical-shift variations, particularly at C-1, C-5, and C-29, which are consistent with typical gauche/anti effects. The characteristic MS fragment losses of 44 and 58 Da, corresponding to acetaldehyde and acetone, respectively, also match the known fragmentation behavior of bourjotone **(319)**, thereby corroborating the structural and stereochemical assignment [33]. Meanwhile, dysolenticin G (**320**) was obtained from the twigs and leaves of *Dysoxylum lenticellatum* using EtOH extraction [42]. Tetracyclic core closely resembles those of compounds **2**–**4**, differing mainly by the absence of six carbons from the side chain, which indicates a structurally degraded tirucallane framework. In the HMBC spectrum, key correlations from δ_H_ 2.12 (3H, s, CH₃-21) to δ_C_ 61.5 (C-17) and 209.8 (C-20), together with a correlation from δ_H_ 2.83 (1H, t, *J* = 8.4 Hz, H-17) to δ_C_ 209.8, support the presence of an acetyl group at C-17. Furthermore, analysis of NOESY spectrum enabled assignment of the relative configuration, with the observed NOESY cross-peak between H-17 and H_3_-30 indicating a β-orientation of H-17 [42].

Seven degraded tirucallane analogues were isolated from the stem bark of *Aphanamixis grandifolia*, through EtOH/H_2_O extraction, including 3α-hydroxy-21α-ethoxy-24,25,26,27-tetranortirucall-7-ene 23(21)-lactone (**321**) as representative tetranortirucallane-type metabolites. In addition, four degraded ursane-derived congeners were also reported, including 3-oxo-urs-12-en-28-oic acid 17β-(2-oxotetrahydrofuran-3-yl) ester (**322**), 3β-hydroxy-urs-12-en-28-oic acid 17β-(2-hydroxy-2-oxotetrahydrofuran-3-yl) ester (**323**), 3-oxo-urs-12-en-28-oic acid 17β-(3-methoxy-2-oxotetrahydrofuran-3-yl) ester (**324**), and 3β,19-dihydroxy-urs-12-en-28-oic acid-23-al (**325**), along with the lactonized metabolite 3β-hydroxy-urs-12-en-28,23-olide (**326**) [53]. These compounds can be classified into three groups based on the key functional-group features. Compounds **321–322** are characterized by variation at C-3, bearing either a hydroxyl or a carbonyl group together with an acetal functionality in the γ-lactone ring. Other compounds **322**–**326** possess a carbonyl group at C-3 in combination with an acetal moiety, while compounds **325–326** lack the acetal functionality in the γ-lactone ring. These structural distinctions emphasize the role of the oxidation state at C-3 and the presence or absence of acetal functionality in defining the overall chemical diversity in the series [53]. Beyond *Aphanamixis*, two structurally related tetranortirucallane lactones-3-oxo-24,25,26,27-tetranortirucall-7-ene-23(21)-lactone (**327**) and 3-hydroxy-24,25,26,27-tetranortirucall-7-ene-23(21)-lactone (**328**) were isolated from the bark of *Dysoxylum laxiracemosum* using MeOH extraction [92]. Collectively, these compounds represent simplified downstream metabolites of tirucallanes, indicating that progressive side-chain truncation and lactone formation contribute to degraded triterpenoid diversity across Meliaceae taxa. (20*S*)-3-oxo-tirucalla-25-nor-7-en-24-oic acid (**329**) from *E. congoënse*, and 4,4,14-trimethyl-3-oxo-24-nor-5α,13α,14β,17α,20*S*-chol-7-en-23-oic acid (**330**) from *Toona sinensis* stem bark [38, 39], further underscore the diversity of acyclic tirucallane triterpenoids across Meliaceae species. Furthermore, methyl 3-oxotirucalla-7,24-dien-21-oate (**330**) was isolated from the stem bark of *Aphanamixis grandifolia* [40].

### *Seco*-tirucallane-type

**Fig. 11:**
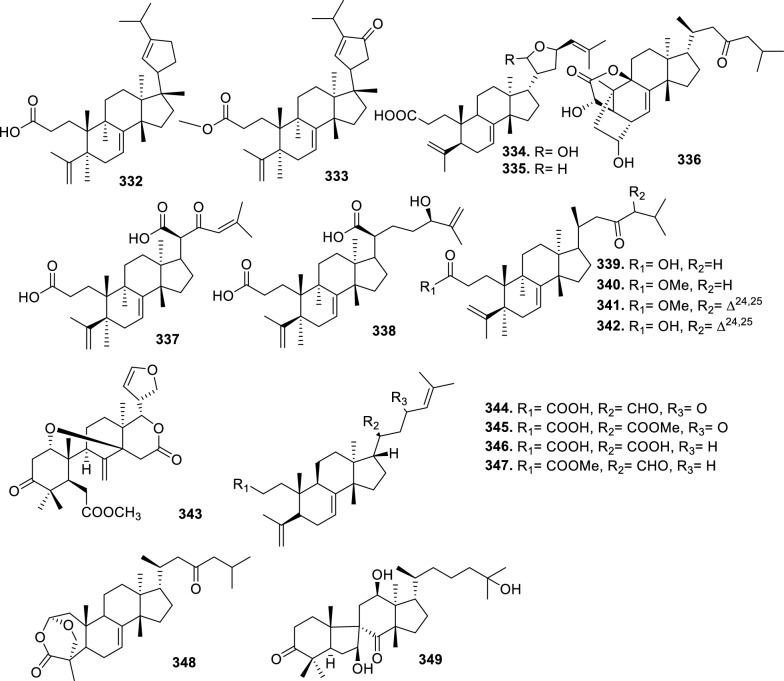
Seco-tirucallane-type **332–349**

*Seco*-tirucallane triterpenoids are oxidative ring-opened derivatives of tirucallane skeleton, characterized by specific C–C bond cleavage (e.g., 3,4-*seco*) and subsequent functionalization, while retaining the core stereochemistry of the parent framework [32]. *Seco*-tirucallane subclass comprises eighteen structurally modified congeners (**332–349**) characterized by oxidative C-3/C-4 bond cleavage and subsequent formation of epoxy- or diacid-type frameworks, representing advanced downstream metabolites relative to intact tirucallanes (Table 3, Fig. 11). The earliest reported members, *seco*-tiaminic acids B (**332**) and C (**333**), were isolated from the roots of *Entandrophragma congoënse* using CH₂Cl₂/MeOH extraction [32], marking the first evidence of ring-opening tirucallanes in the genus. Two epoxy-functionalized dicarboxylic analogues, guareolide A (**334**) and guareoic acid B (**335**), were subsequently obtained from the aerial parts of *Guarea guidonia* through MeOH extraction [52], suggesting a conserved oxidative rearrangement of C-21/C-23 epoxidation.

**Table 3:** *Seco-*tirucallane compounds from Meliaceae family

No	Compounds	Species	Parts	Extract	References
**332**	*Seco-*tiaminic acids B	*Entandrophragma congoënse*	Root	CH_2_Cl_2_/MeOH	[32]
**333**	*Seco-*tiaminic acids C	*E. congoënse*	Root	CH_2_Cl_2_/MeOH	[32]
**334**	3,4-*seco-*tirucalla-4(28),7(8),24(25)-trien-21-hydroxy-21,23-epoxy-3-oic acid, (Guareolide A)	*Guarea guidonia*	Aerial	MeOH	[52]
**335**	3,4-*seco-*tirucalla-4 (28),7 (8),24 (25)-trien-21,23-epoxy-3-oic acid, (guareoic acid B)	*G. guidonia*	Aerial	MeOH	[52]
**336**	Aphanamgrandiol A	*Aphanamixis grandifolia*	Stem	EtOH	[17]
**337**	23-oxo-3,4-*seco*tirucalla-4(28),7,24-trien-3,21-dioic acid	*Entandrophragma angolense*	Stem bark	EtOH	[93]
**338**	24(*R/S*)-hydroxy-3,4-secotirucalla-4(28),7,25(26)-trien-3,21-dioic acid	*E. angolense*	Stem bark	EtOH	[93]
**339**	Aphanamgrandin G	*Aphanamixis grandifolia*	Stems	EtOH	[34]
**340**	Aphanamgrandin H	*A. grandifolia*	Stems	EtOH	[34]
**341**	Aphanamgrandin I	*A. grandifolia*	Stems	EtOH	[34]
**342**	Aphanamgrandin J	*A. grandifolia*	Stems	EtOH	[34]
**343**	Methyl angolensate	*Entandrophragma angolense*	Bark	EtOH	[16]
**344**	3,4-*seco*tirucalla-23-oxo-4(28),7,24-trien-21-al-3-oic acid	*E. Angolese*	Leaves	*n-*hexane	[37]
**345**	3,4-*seco*tirucalla-23oxo-4(28),7,24-trien-3,21-dioicacid (21-methyl ester)	*E. Angolese*	Leaves	*n-*hexane	[37]
**346**	3,4-*seco-*tirucalla-4(28),7,24-trien-3,21-dioic acid	*E. Angolese*	Leaves	*n-*hexane	[37]
**347**	3,4-*seco-*tirucalla-4(28),7,24-trien-3,21-dioic acid, 3-methyl ester	*E. Angolese*	Leaves	*n-*hexane	[37]
**348**	2,3 *seco*tirucalla-2,3,2,29-diepoxy-7-ene-3,23-dione	*Aphanamixis grandifolia*	Leaves and twigs	EtOH	[99]
**349**	7β,25-dihydroxy-7,9-cycro-7,8-*seco-*tirucallane-3,8-dione	*Chukrasia tabularis*	Fruit	EtOH	[42]

Additional representatives include aphanamgrandiol A (**336**) from the stems of *Aphanamixis grandifolia* using EtOH extraction [17], followed by two further derivatives from *Entandrophragma angolense*, namely 23-oxo-3,4-*seco*-tirucalla-4(28),7,24-trien-3,21-dioic acid (**337**) and 24(*R*)-hydroxy-3,4-*seco*-tirucalla-4(28),7,25(26)-trien-3,21-dioic acid (**338**) [93]. The 3,4-*seco*tirucallane derivatives (**337)** and (**338)** are closely related to previously reported analogues whose absolute configurations were established by combined ECD calculations and ^13^C NMR DP4 analysis, confirming a 22*R* configuration. The strong agreement between experimental and calculated ECD spectra supports conservation of the core *seco*-tirucallane stereochemistry, with structural differences arising mainly from side-chain oxidation and hydroxylation [93]. A series of four structurally related congeners, namely aphanamgrandins G (**339**), H (**340**), I (**341**), and J (**342**), were isolated from *Aphanamixis grandifolia* stems through EtOH–H_2_O extraction [34]. That compounds presence of oxygen functionalities at C-2 and C-3 is a key prerequisite for 2,3-cleavage of the A ring, while 3,4-cleavage can occur with only a carbonyl group at C-3. This selective ring opening has been rationalized by a Baeyer–Villiger type oxidation mechanism, which facilitates oxidative rearrangement of tirucallane A ring. The widely studied methyl angolensate (**343**), obtained from the bark of *Entandrophragma angolense* using EtOH extraction [16], represents a methyl-ester-stabilized *seco*-tirucallane frequently cited as a chemotaxonomic marker within *Entandrophragma* spp. Several additional 3,4-*seco*-tirucallane acids **344–347** were isolated from leaves of *Entandrophragma angolense* using *n*-hexane extraction [37], further expanding the metabolic flexibility observed across the genus.

Two highly modified congeners showing diepoxy and cyclization rearrangements, namely 2,3-*seco*-tirucalla-2,3;2,29-epoxy-7-ene-3,23-dione (**348**) from *Aphanamixis grandifolia* [53] and 7β,25-dihydroxy-7,9-cyclo-7,8-*seco*-tirucallane-3,8-dione (**349**) from the fruits of *Chukrasia tabularis* [42], represent some of the most structurally divergent members within the subclass. The relative configuration of compound** 349** was assigned based on NOESY data. Key NOESY correlations, including H_3_-28/H-5/H-7 and H_3_-18/H-12, support β-oriented hydroxyl groups at C-7 and C-12, while correlations among H-17, CH_3_-30, and H_3_-21 indicate a β-orientation of the C-21 methyl group. These features confirm the assigned structure of 7β,25-dihydroxy-7,9-cyclo-7,8-*seco*-tirucallane-3,8-dione **(349)**, indicating a highly modified *seco*-tirucallane framework [42].

### Heteroatom tirucallane-type

**Fig. 12:**
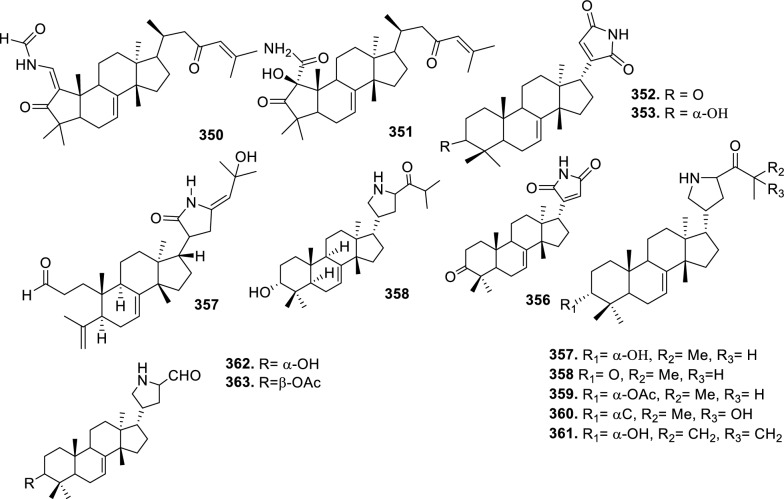
Heteroatom tirucallane-type **350–363**

Heteroatom tirucallane triterpenoids represent oxidatively transformed tirucallane derivatives in which heteroatoms, predominantly oxygen, are incorporated into the carbon framework, producing heterocyclic or bridged architectures while preserving biosynthetic tirucallane lineage. This subclass consists of fourteen structurally altered compounds (**350**–**363**) featuring nitrogen- or oxygen-linked esterified side chains, reflecting an advanced stage of post-terpenoid structural diversification distinct from conventional oxygenated tirucallane analogues (Table 4, Fig. 12) [72]. Additional compounds include aphanamgrandins E (**350**) and F (**351**) from the stems of *Aphanamixis grandifolia* extracted with EtOH [34]. These compounds represent heteroatom tirucallane derivatives characterized by pronounced A-ring modification and incorporation of nitrogen- and oxygen-containing functionalities. Both preserve tirucallane B/C/D ring system and side chain, while diversification occurs through 2,3-*seco* cleavage and 1,3-*abeo* rearrangement of the A ring. Compound **350** features an unusual five-membered A ring with an exocyclic Δ^1^,^2^ double bond and a formamido substituent at C-2, while compound **351** lacks this unsaturation but has a β-oriented hydroxyl and a carboxamide group at C-1 [34]. The first reported derivative, laxiracemosin H (**352**), was isolated from the bark of *Dysoxylum laxiracemosum* using methanolic extraction [92], followed by dysolenticin J (**353**) from the twigs and leaves of *Dysoxylum lenticellatum* through ethanol extraction [42]. A further representative, congoensin A (**354**), was obtained from the stem bark of *Entandrophragma congoënse* using CH₂Cl₂/MeOH extraction [39], demonstrating the occurrence of heteroatom substitution beyond epoxide- and lactone-type modifications commonly encountered in the family. Two uncommon pyrrolidinyl-esterified uranane analogues, namely 3β-hydroxy-urs-12-en-28-oic acid-17β-[1-(2-methylpropanoyl)pyrrolidin-2-yl] ester (**355**) and 3-oxo-urs-12-en-28-oic acid-17β-(2,5-dioxopyrrolidin-1-yl) ester (**356**) were subsequently isolated from the stem bark of *Aphanamixis grandifolia* using ethanol extraction [40], representing rare examples of N-heterocyclic incorporation into tirucallane-derived uranane scaffolds.

**Table 4:** Heteroatom tirucallane compounds from Meliaceae family

No	Compounds	Species	Parts	Extract	References
**350**	Aphanamgrandin E	*Aphanamixis grandifolia*	Stems	EtOH	[34]
**351**	Aphanamgrandin F	*A. grandifolia*	Stems	EtOH	[34]
**352**	Laxiracemosin H	*Dysoxylum laxiracemosum*	Bark	MeOH	[92]
**353**	Dysolenticin J	*D. lenticellatum*	Twigs and leaves	EtOH	[101]
**354**	Congoensin A	*Entandrophragma congoënse*	Stem bark	MeOH	[39]
**355**	3β-hydroxy-urs-12-en-28-oic acid-17β-[1-(2-methylpropanoyl)pyrrolidin-2-yl] ester	*A. grandifolia*	Stem bark	EtOH	[40]
**356**	3-oxo-urs-12-en-28-oic acid-17β-(2,5-dioxopyrrolidin-1-yl) ester	*A. grandifolia*	Stem bark	EtOH	[40]
**357**	Laxiracemosin A	*Dysoxylum laxiracemosum*	Bark	MeOH	[92]
**358**	Laxiracemosin B	*D. laxiracemosum*	Bark	MeOH	[92]
**359**	Laxiracemosin C	*D. laxiracemosum*	Bark	MeOH	[92]
**360**	Laxiracemosin D	*D. laxiracemosum*	Bark	MeOH	[92]
**361**	Laxiracemosin E	*D. laxiracemosum*	Bark	MeOH	[92]
**362**	Laxiracemosin F	*D. laxiracemosum*	Bark	MeOH	[92]
**363**	Laxiracemosin G	*D. laxiracemosum*	Bark	MeOH	[92]

Seven related congeners, including laxiracemosins A (**357**), B (**358**), C (**359**), D (**360**), E (**361**), F (**362**), and G (**363**), were isolated from the bark of *Dysoxylum laxiracemosum* through MeOH extraction [92]. The laxiracemosins constitute a coherent series of heteroatom-containing tirucallane triterpenoids that differ mainly by oxidative and substituent variations at C-3 and the C-17 side chain. More specifically, laxiracemosin A (**357**) has a pentacyclic tirucallane-7-ene core bearing a pyrrole ring attached at C-17, as established by HMBC correlations, representing a hallmark feature of this subclass. laxiracemosins B–E (**358–361**) are close analogues of **361**, arising from stepwise modifications at C-3 (3-oxo, 3-acetoxy) and side-chain oxidation or unsaturation, while retaining the same heteroatom-substituted framework. Another member, laxiracemosin F (**362**), shows a truncated side chain with an aldehyde-conjugated pyrrole, indicating a degraded derivative of the parent tirucallane skeleton. Laxiracemosin G (**363**) is C-3 acetate of **362**, with coupling constants of H-3 supporting a distinct stereochemical orientation. Collectively, these correlations demonstrate that compounds **357**–**363** represent a structurally related heteroatom tirucallane series derived through oxidation, acetylation, and side-chain degradation while preserving the same biosynthetic origin [92]. This subclass, in general, reflects one of the most structurally divergent groups of tirucallane derivatives, characterized by late-stage esterification or amino-acyl substitution of nitrogen-bearing moieties, expanding the chemical diversity observed in Meliaceae terpenoids.

### Highly rearranged tirucallane-type

**Fig. 13:**
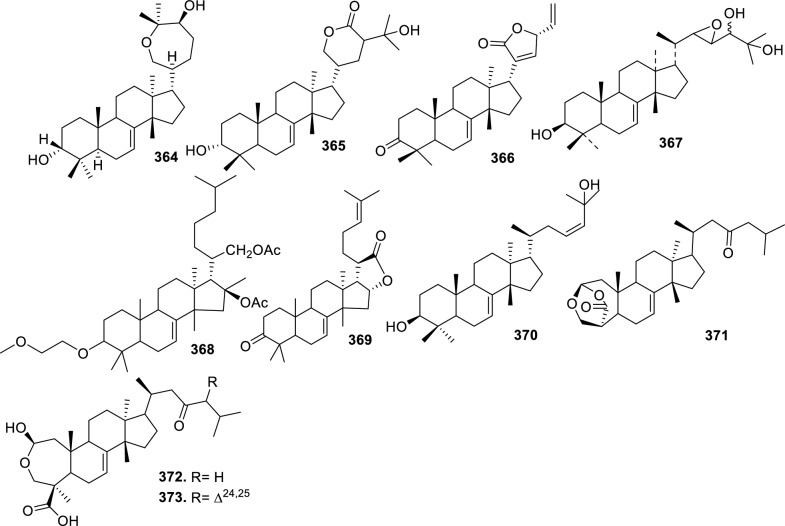
Highly rearranged tirucallane-type **364–373**

Highly rearranged tirucallane triterpenoids comprise derivatives that have experienced profound structural remodelling beyond the typical tirucallane scaffold. This class is distinguished by significant ring reorganization, ring opening followed by reclosure, side-chain transposition, or ketal/spiro-ketal formation, resulting in unconventional carbon frameworks that remain traceable to tirucallane biosynthetic origin. Accordingly, the highly rearranged tirucallane group (**364**–**373**) constitutes a small but structurally striking subset that departs significantly from the classical tirucallane biosynthetic topology (Table 5, Fig. 13) [79, 84]. The first member, sapelins B (**364**), was isolated from leaves of *Trichilia hispida* using ethanol extraction [33], marking one of the earliest examples of deep post-cyclization modification in Meliaceae. Further structural diversity was demonstrated by dysolenticin A (**365**) and dysolenticin B (**366**) obtained from the twigs and leaves of *Dysoxylum lenticellatum* through ethanol extraction [84], suggesting that extensive rearrangement capacity may be genus-linked rather than species-specific (Table 5).

**Table 5:** Highly rearranged tirucallane compounds from Meliaceae family

No	Compounds	Species	Parts	Extract	References
**364**	Sapelin B	*Trichilia hispida*	Leaves	EtOH	[33]
**365**	Dysolenticin A	*Dysoxylum lenticellatum*	Twigs and leaves	EtOH	[101]
**366**	Dysolenticin B	*D. lenticellatum*	Twigs and leaves	EtOH	[101]
**367**	22,23-epoxy-7-tirucalla-7-ene-3β,24,25-triol	*Dysoxylum hainanense*	Stem bark	EtOAc	[23]
**368**	16β,21-dihydroxyeupha-7,24-dien-3-one ketal diacetate	*M. azedarach* L	Fruits	C_6_H_14_	[37]
**369**	16-epikulactone	*M. azedarach* L	Fruits	C_6_H_14_	[37]
**370**	(23*Z*)-3β,26-dihydroxytirucalla-7,23-diene	*Dysoxylum lukii*	Stem barks	EtOH	[59]
**371**	Aphanamgrandin A	*A. grandifolia*	Stems	EtOH	[34]
**372**	Aphanamgrandin C	*A. grandifolia*	Stems	EtOH	[34]
**373**	Aphanamgrandin D	*A. grandifolia*	Stems	EtOH	[34]

The compound, 22,23-epoxy-7-tirucalla-7-ene-3β,24,25-triol (**367**) was isolated from the stem bark of *Dysoxylum hainanense* using EtOAc extraction [23], showing extensive rearrangement accompanied by regio-specific epoxidation and multi-hydroxylation. Two unusual fruit-derived rearranged tirucallanes, namely 16β,21-dihydroxyeupha-7,24-dien-3-one ketal diacetate (**368**) and 16-epikulactone (**369**), were subsequently isolated from the fruits of *Melia azedarach* using C_6_H_14_ extraction [37], indicating that non-polar extraction conditions may favor the detection of deep oxidation–ketal transformation products in the species. The final compound, (23*Z*)-3β,26-dihydroxytirucalla-7,23-diene (**370**), was isolated from the stem bark of *Dysoxylum lukii* via EtOH extraction [59]. Additionally, three rearranged tirucallane, namely aphanamgrandin A (**371**), C (**372**), and D (**373**), were obtained from the stems of *Aphanamixis grandifolia* using EtOH extraction [34], further expanding the structural diversity observed across the family.

## Biological activities

**Fig. 14:**
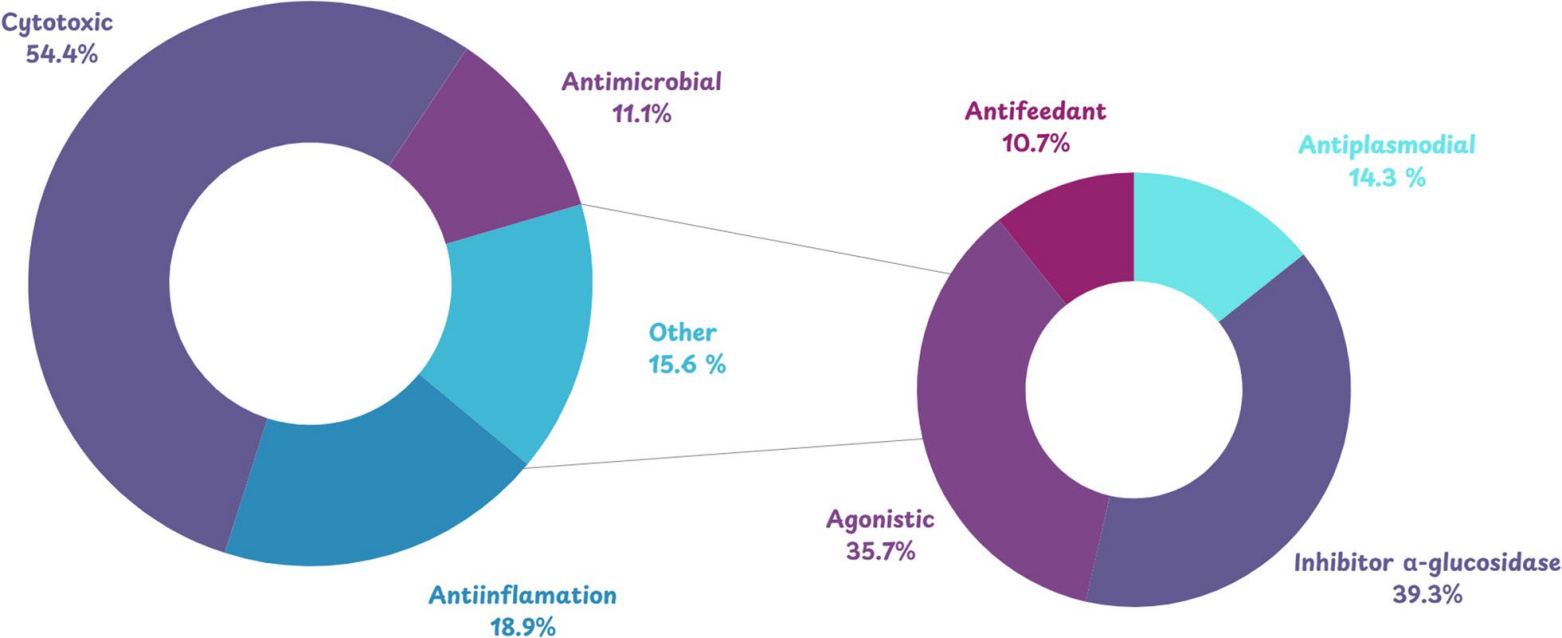
Biological activities of tirucallane-type triterpenoids from the Meliaceae family

Higher plants produce abundant tirucallane-type triterpenoids, a prominent class of secondary metabolites with diverse structural variations. Among various plant families that produce these compounds, Meliaceae family has been extensively investigated. However, a comprehensive review regarding the biological activities of tirucallane-type triterpenoids remains lacking. These compounds have a wide range of bioactivities, including cytotoxic, antimalarial, anti-inflammatory, antibacterial, antifungal, antidiabetic, anti-tobacco mosaic virus, antifeedant, and allelopathic effects. Biological activities of Meliaceae tirucallane-type triterpenoids are shown across Tables 6, 7, 8, 9 and Fig. 14 for clarity and comparison. Table 6 summarizes the available cytotoxicity data, and Table 7 compiles reported antimicrobial activities, including both antibacterial and antifungal evaluations. Table 8 shows anti-inflammatory studies, and finally, Table 9 captures other bioactivities reported for this metabolite class, including antimalarial, antidiabetic, anti-tobacco mosaic virus, antifeedant, and allelopathic effects. Collectively, the results show that tirucallane-type triterpenoids span multiple pharmacological endpoints and Meliaceae plants remain a rich as well as practical starting point for finding new bioactive leads [102–108].

**Table 6:** Cytotoxic activity of tirucallane compounds

Species	Parts	Compounds	Bioactivities	References
*C. sinensis*	Cortex	21α-methoxy-24,25-dihydroxyapotirucall-7-en-3-one **(166)**	IC_50_ = 3.3–9.9 mg/mL (P-388)	[72]
*T. hispida*	Leaves	Hispidol B **(3)**	IC_50_ = 5.2 mg/mL (P-388)	[33]
*A. grandifolia*	Stem bark	3α-hydroxy-21α-methoxy-24,25,26,27-tetranortirucall-7-ene 23(21)-lactone **(321)**	IC_50_ = 42.2 μM (MCF-7), 37.6 μM (HeLa), 31.4 μM (HepG2), 26.1 μM (SGC-7901), 24.2 μM (BGC-823)	[53]
		3-oxo-24,25,26,27-tetranortirucall-7-ene-23(21)-lactone** (125)**	IC_50_ = 67.1 μM (MCF-7), 24.3 μM (HeLa), 32.6 μM (HepG2), 21.3 μM (SGC-7901), 12.8 μM (BGC-823)	
		3-oxo-urs-12-en-28-oic acid 17β-(2-oxotetrahydrofuran-3-yl) ester **(322)**	IC_50_ = 166.5 μM (MCF-7), 95.5 μM (HeLa), 91.2 μM (HepG2), 70.9 μM (SGC-7901), 154.4 μM (BGC-823)	
		3β-hydroxy-urs-12-en-28-oic acid 17β-(2-hydroxy-2-oxotetrahydrofuran-3-yl) ester **(323)**	IC_50_ = 76.2 μM (MCF-7), 52.8 μM (HeLa), 71.8 μM (HepG2), 71.9 μM (SGC-7901), 41.7 μM (BGC-823)	
		3-oxo-urs-12-en-28-oic acid 17β-(3-methoxy-2-oxotetrahydrofuran-3-yl) ester **(324)**	IC_50_ = 50.2 μM (MCF-7), 76.2 μM (HeLa), 58.3 μM (HepG2), 108.0 μM (SGC-7901), 126.6 μM (BGC-823)	
		3β,19-dihydroxy-urs-12-en-28-oic acid-23-al **(325)**	IC_50_ = 57.2 μM (MCF-7), 64.1 μM (HeLa), 34.2 μM (HepG2), 38.2 μM (SGC-7901), 38.8 μM (BGC-823)	
		3β-hydroxy-urs-12-en-28,23-olide **(326)**	IC_50_ = 97.6 μM (MCF-7), 64.6 μM (HeLa), 25.7 μM (HepG2), 15.7 μM (SGC-7901), 33.0 μM (BGC-823)	
*D. lenticellatum*	Twigs and leaves	Dysolenticin A **(365)**	IC_50_ = 53.3 μM (MCF-7)	[101]
		Dysolenticin C **(167)**	IC_50_ ≥ 100 μM (MCF-7), IC_50_ = 15.3 μM (HeLa)	
		Dysolenticin D **(168)**	IC_50_ = 29.9 μM (HeLa) IC_50_ = 10.3 μM (MCF-7)	
*A. grandifolia*	Stem bark	(13α,14β,17α,23*Z*)-25-methoxy-21,23-epoxylanosta-7,20(22),23-triene-3,21-dione **(170)**	IC_50_ = 53.3 μM (MCF-7), IC_50_ = 21.4 μM (HeLa)	[73]
		(3*R*,5*R*,9*R*,10*R*,13*S*,14*S*,17*S*)-17-{(2*R*,3*S*,5*R*)-5-[(2*S*)-3,3-dimethyloxiran-2-yl]-2,5-dimethoxytetrahydrofuran-3 yl}-2,3,4,5,6,9,10,11,12,13, 14,15,16,17-tetradecahydro-4,4,10,13,14-pentamethyl-1H-cyclopenta[a]phenanthren-3-ol **(257)**	IC_50_ ≥ 100 μM (MCF-7), IC_50_ = 15.3 μM (HeLa)	
		(5*R*,9*R*,10*R*,13*S*,14*S*,17*S*)-17-{(2*R*,3*S*,5*R*)-5-[(2S)-3,3-dimethyloxiran-2-yl]-2,5-dimethoxytetrahydrofuran-3-yl}-1, 2, 4, 5, 6, 9, 10, 11, 12, 13, 14, 15, 16, 17-tetradecahydro-4,4,10,13,14-pentamethyl-3H-cyclopenta[a]phenanthren-3-one **(258)**	IC_50_ = 10.3 μM (MCF-7), IC_50_ = 29.9 μM (HeLa)	
*A. grandifolia*	Stems	Aphanamgrandin A **(371)**	IC_50_ = 160.74 μM (HeLa)	[34]
*C. pentandrus*	Stem bark	Indicalilacol B **(206)**	IC_50_ = 15.0 µg/mL (KB), 16.1 µg/mL (KB-C2), 19.0 µg/mL (KB-C2 + colchicine), 15.7 µg/mL (MCF-7)	[79]
*A. indica*	Fruit	Indicalilacol D **(12)**	IC_50_ = 4.60 µg/mL (KB-C2 + colchicine), 9.04 µg/mL (MCF-7)	[36]
		Meliasenin S **(175)**	Not active; MDR-reversal only → IC_50_ = 6.48 µg/mL (KB-C2 + colchicine)	
		Meliantriol **(176)**	IC_50_ = 25.7 µg/mL (KB), 15.9 µg/mL (KB-C2), 20.9 µg/mL (KB-C2 + colchicine), 0.27 µg/mL (MCF-7)	
		Meliasenin T **(177)**	IC_50_ = 17.1 µg/mL (KB), 15.6 µg/mL (KB-C2), 6.67 µg/mL (KB-C2 + colchicine), 16.0 µg/mL (MCF-7)	
*Entandrophragma congoënse*	Stem bark	(20*S*)-3-oxo-tirucalla-25-nor-7-en-24-oic acid **(329)**	IC_50_ = 7.8 µM (HL-60), 15.2 µM (A549), 18.4 µM (MCF-7), 12.1 µM (HepG2)	
*E. congoënse*	Root	Piscidinol A **(1)**	IC_50_ = 11.6 µM (HL-60), 14.5 µM (A549), 17.9 µM (MCF-7), 10.7 µM (HepG2); HL-60: 3.3 µg/mL; KB: 8.5 µg/mL; HeLa: 9.9 µg/mL IC_50_ 1.16 µM (A549), 3.01 µM (BGC-823 IC_50_ = 6.9 µM (MKN-28), > 10 µM (A-549), 8.5 µM (MCF-7)	[32]
*Trichilia hispida*	Leaves	Hispidol B **(3)**	IC_50_ ≥ 40 µM (HL-60), 32.4 µM (A549), 35.7 µM (MCF-7), 28.6 µM (HepG2)	[33]
*Toona sinensis*	Stem bark	4,4,14-trimethyl-3-oxo-24-nor-5α,13α,14β,17α,20*S*-chol-7-en-23-oic acid **(331)**	IC_50_ = 9.4 µM (HL-60), 13.5 µM (A549), 16.7 µM (MCF-7), 8.9 µM (HepG2)	[52]
		Bourjotinolone B **(17)**	IC_50_ = 8.1 µM (HL-60), 11.3 µM (A549), 14.8 µM (MCF-7), 9.6 µM (HepG2)	
*Guarea guidonia*	Aerial	3,4-*seco-*tirucalla-4 (28),7 (8),24 (25)-trien-21,23-epoxy-3-oic acid, (guareoic acid B) **(335)**	IC_50_ = 39 µM (Jurkat), 55 µM (HeLa), 75 µM (MCF-7), > 100 µM (PBMC)	[52]
		Flindssone **(183)**	IC_50_ = 25 µM (Jurkat), 27 µM (HeLa), 50 µM (MCF-7), > 100 µM (PBMC)	
		Picroquassin E **(184)**	IC_50_ = 88 µM (Jurkat), > 100 µM (HeLa), > 100 µM (MCF-7)	
*A. grandifolia*	Stems	Leucophyllone **(11)**	IC_50_ ≥ 30 µM (A549), > 30 µM (SK-OV-3), > 30 µM (SK-MEL-2), > 30 µM (XF498)	[34]
*T. Ciliatawas*	Bark	Toonaciliatavarin E **(74)**	IC_50_ = 17.1 ± 1.4 µM (MCF-7), 22.6 ± 3.8 µM (MCF-7/ADR), 10.2 ± 1.1 µM (KB), 32.1 ± 2.3 µM (KB/VCR), 19.4 ± 1.9 µM (SMMC-7721), 11.2 ± 0.9 µM (K562)	[54]
*Trichilia hispida*	Leaves	Hispidol B **(3)**	IC_50_ = 6.2 µg/mL (HL-60), 7.4 µg/mL (KB), 8.1 µg/mL (HeLa)	[33]
		Hispidol A **(2)**	IC_50_ = 3.8 µg/mL (HL-60), 5.9 µg/mL (KB), 6.7 µg/mL (HeLa)	
*Cedrela fissilis*	Roots	Nilocetin **(265)**	IC_50_ = 8.1 µg/mL (HL-60), 9.3 µg/mL (KB), 10.0 µg/mL (HeLa)	[25]
*T. ciliata* var.* ciliata*	Leaves and twigs	Toonaciliatin L **(35)**	IC_50_ = 21.8 µM (MCF-7), 29.7 µM (KB), 25.1 µM (K562)	[24]
*C. odorata*	Leaves and twigs	Altissimanin A **(267)**	IC_50_ = 25 µM (MCF-7), 23 µM (HepG2), 19 µM (HeLa)	[25]
*T. ciliata* var. *ciliata, T. ciliata* var.* henryi*	Stem bark, leaves and twigs	3-episapelin A **(217)**	IC_50_ = 50 µM (MCF-7), 43 µM (HepG2), 38 µM (HeLa)	[25]
*T. ciliata* var. *ciliata, T. ciliata* var.* henryi*	Stem bark, leaves and twigs	Toonaciliatavarin D **(216)**	IC_50_ ≥ 50 µM (MCF-7), > 50 µM (MCF-7/ADR), 39.5 ± 2.0 µM (KB), > 50 µM (KB/VCR), 31.4 ± 2.4 µM (SMMC-7721), 43.1 ± 2.1 µM (K562)	[25]
*T. ciliata*	Leave and twigs	Toonaciliatin K **(192)**	IC_50_ = 24.6 µM (MCF-7), 27.1 µM (KB), 28.4 µM (K562)	
*T. ciliata* var. *ciliata, T. ciliata* var.* henryi*	Stem bark, leaves and twigs	Hispidone **(218)**	IC_50_ = 6.5 µg/mL (HL-60), 8.2 µg/mL (KB), 9.0 µg/mL (HeLa)	[25]
*Dysoxylum macranthum*	Stem barks	Dymacrin B **(41)**	IC_50_ = 5.6 µg/mL (KB)	[26]
		Dymacrin C **(42)**	IC_50_ = 5.0 µg/mL (KB)	[26]
		Dymacrin H **(268)**	IC_50_ = 8.3 µg/mL (KB)	
		Dymacrin J **(270)**	IC_50_ = 1.0 µg/mL (KB)	
*M. azedarach*	Fruits	3-β-tigloylmelianol **(271)**	IC_50_ = 91.8 µg/mL (A549); CC50 = 64.7 µg/mL (A549)	[27]
*Swietenia humilis Zucc*	Seeds	Melianone **(263)**	IC_50_ = 3.6 μg/mL (A549)	[43]
*Dysoxylum variabile*	Stem bark	Tirucalla-7,24-diene-3β,23-diol **(49)**	IC_50_ = 10.0 µg/mL (KB cells)	[28]
*D. variabile*	Stem bark	Dyvariabilin E **(273)**	IC_50_ = 10.1 µg/mL (KB cells)	
		Dyvariabilin F **(274)**	IC_50_ = 10.1 µg/mL (KB cells)	
		Dyvariabilin G **(275)**	IC_50_ = 8.4 µg/mL (KB cells)	
		Niloticin **(276)**	IC_50_ = 9.2 µg/mL (KB cells)	
		Dyvariabilin H **(266)**	IC_50_ = 8.4 µg/mL (KB cells)	
*M. azedarach*	Ripe fruit	21α-methylmelianodiol **(191)**	IC_50_ ≥ 10 µM (A549), > 10 µM (BGC-823) IC_50_ 92.8 (HepG2), 47.6 (K562), 38.4 (SGC7901), 19.7 (HL60)	[29]
		21β-methylmelianodiol **(197)**	IC_50_ ≥ 10 µM (A549), > 10 µM (BGC-823) IC_50_ 27.9 (HepG2), 19.7 (K562), 27.9 (SGC7901), 16.8 (HL60)	
*Chisocheton pentandrus*	Stem bark	Melianodiol **(205)**	IC_50_ ≥ 10 µM (MKN-28, A-549, MCF-7)	[79]
*T. ciliata* var *Pubescens*	Stem bark	24*S*,25-dihydroxytirucall-7-en-3-one **(110)**	IC_50_ = 9.1 µM (MKN-28), 10.2 µM (A-549), > 10 µM (MCF-7)	[60]
		Niloticin **(276)**	IC_50_ ≥ 50 µM (HepG2), < 50 µM (Hep3B)	
*T. ciliata var* *Pubescens*	Stem bark	Dihydroniloticin **(108)**	IC_50_ ≅ 8.2 µM ( HT-1080)	[60]
*M. azedarach*	Ripe fruit	Meliasenin S **(175)**	IC_50_ ≥ 100 (HepG2), > 100 (K562), > 100 (SGC7901), 24.1 (HL60)	[29]
		Meliasenin T **(177)**	IC_50_ = 31.7 (HepG2), 13.7 (K562), 29.6 (SGC7901), 6.3 (HL60)	
		21β, 25-dimethylmelianodiol **(199)**	IC_50_ ≥ 100 (HepG2), 89.5 (K562), > 100 (SGC7901), 46.0 (HL60)	
		21, 25-dimethylmelianodiol **(200)**	IC_50_ ≥ 100 (HepG2), 25.2 (K562), 25.6 (SGC7901), 19.8 (HL60)	
		(21*R*, 23*R*)-epoxy-24-hydroxy-21β-methoxytirucall-7, 25-dien-3-one **(201)**	IC_50_ = 48.3 (HepG2), 42.1 (K562), 49.4 (SGC7901), 10.8 (HL60)	
		Methoxytirucall-7, 25-dien-3-one **(202)**	IC_50_ = 38.5 (HepG2), 34.3 (K562), 38.5 (SGC7901), 81.7 (HL60)	
		Tirucall-7, 25-dien-3-one **(204)**	IC_50_ = 41.6 (HepG2), 20.9 (K562), 41.6 (SGC7901), 22.3 (HL60)	
		21-oxo-melianodiol 14 21-oxo-meliantriol 15 toosendanic acid A **(60)**	IC_50_ ≥ 100 (HepG2), not tested (K562 & SGC7901), 52.4 (HL60)	
		Methyl kulonate (**47**)	IC_50_ = 81.1 (HepG2), not tested (K562 & SGC7901), 71.3 (HL60)	
*M. azedarach*	Fruit	Meliastatin **(61)**	IC_50_ = 62.9 (HepG2), not tested (K562 & SGC7901), 18.0 (HL60)	[27]
*A. grandifolia*	Stem bark	3α,24β,25-trihydroxy-21,21-dimethoxy-23-oxotirucall-7-ene **(72)**	IC_50_ = 11.2 µM (NCI-H460); 19.8 µM (HeLa)	[99]
*A. grandifolia*	Stem bark	3α-acetoxy-21β-methoxy-24,25,26,27-tetranortirucall-7-ene-23(21)-lactone **(281)**	IC_50_ = 38.9 µM (NCI-H460); 51.0 µM (HeLa)	[99]
*Melia toosendan*	Stem bark	3β,16β-hydroxytirucalla-7,24(25)-dien-21,23-olide **(282)**	IC_50_ = 5.6 µg/mL (A549); 4.3 µg/mL (SK-OV-3); 5.7 µg/mL (SK-MEL-2); 3.4 µg/mL (HCT15)	
*M. toosendan*	Stem bark	3β,16β-hydroxytirucalla-7,24(25)-dien-6-oxo-21,23-olide **(283)**	IC_50_ = 4.7 µg/mL (A549); 4.9 µg/mL (SK-OV-3); 5.0 µg/mL (SK-MEL-2); 3.2 µg/mL (HCT15)	[100]
*Dysoxylum laxiracemosum*	Bark	Laxiracemosin A **(357)**	IC_50_ = 3.1 µM (HL-60); 9.5 µM (SMMC-7721); 5.4 µM (A549); 16.8 µM (MCF-7); 7.2 µM (SW480)	[92]
		Laxiracemosin B **(358)**	IC_50_ = 12.8 µM (HL-60); 19.0 µM (SMMC-7721); 13.4 µM (A549); > 20 µM (MCF-7, SW480)	
		Laxiracemosin D **(360)**	IC_50_ = 6.8 µM (HL-60); > 20 µM (A549, MCF-7, SW480); ~ 20 µM (SMMC-7721)	
		Laxiracemosin E **(361)**	IC_50_ = 1.5 µM (HL-60); 2.7 µM (SMMC-7721); 3.7 µM (A549); 5.1 µM (MCF-7); 3.7 µM (SW480)	
*D. laxiracemosum*	Bark	Laxiracemosin F **(362)**	IC_50_ = 15.7 µM (HL-60); 15.6 µM (SMMC-7721); > 20 µM (A549, MCF-7, SW480)	[92]
*A. indica*	Leaves	24,25-epoxy-3β-hydroxy-20-oxo-7-tirucallene **(315)**	IC_50_ = 0.8 ± 0.1 µM	[103]
*M. toosendan*	Fruits	22,23,24,25-diepoxy-3β-hydroxy-7-tirucallene **(316)**	IC_50_ = 2.2 ± 0.2 µM	[87]
*D. hainanense*	Stem bark	24,25-epoxy-3β,23-dihydroxy-7-tirucallene **(277)**	IC_50_ = 1.9 ± 1.3 µM	[23]
*T. Ciliatawas*	Bark	Odoratone **(293)**	IC_50_ = 4.5 ± 1.1 µM	[54]
*M. azedarach*	Fruit	Meliasenin E **(111)**	IC_50_ = 0.65 µM (HL60), 2.8 µM (RPMI 1788)	[61]
*Chukrasia tabularis*	Fruit	7β,25-dihydroxy-7,9-cycro-7,8-*seco-*tirucallane-3,8-dion **(349)**	IC_50_ = 10.45 ± 0.04 µM (K562); 20–35 µM (HeLa, BEL-7402, SGC-7901, A549)	[42]
*Munronia delavayi* Franch	Bark	Toosendine A **(285)**	IC_50_ = 6.53 ± 0.27 µM (NO inhibition RAW 264.7); 12.63 ± 0.28 µM (U20S)	[82]
		Toosendine D **(288)**	IC_50_ = 12.51 ± 0.06 µM (NO inhibition RAW 264.7); 21.37 ± 1.28 µM (U20S)	
		Toosendine E **(118)**	IC_50_ = 21.13 ± 1.52 µM (NO inhibition RAW 264.7); > 50 µM (U20S)	
*D. macranthum*	Stem bark	Dysomollide A **(39)**	IC_50_ = 5.6, 5.0, 8.3, 1.0 µg/mL (KB cells)	
*Chisocheton patens* Blume	Stem bark	Chisopaten A **(68)**	IC_50_ = 4.33 ± 0.009 µM (MCF-7)	
*C. lasiocarpus*	Stem bark	Chisocarpene A **(69)**	IC_50_ = 26.56 ± 1.01 µM (MCF-7)	
*M. toosendan*	Bark	3β,16-dihydroxy-25-hydroperoxytirucalla-7,23(24)-dien-6-oxo acid **(282)**	IC_50_ = 14.2 µM (A549); 16.3 µM (SK-OV-3); 13.4 µM (SK-MEL-2); 15.3 µM (HCT-15)	[100]

**Table 7:** Antimicrobial activity tirucallane compounds

Species	Parts	Compounds	Bioactivities	References
*A. grandifolia*	Stems	Aphanamgrandin C** (372)**	MIC = 1.57–3.13 µg/mL (*Staphylococcus aureus*, MRSA)	[34]
		Aphanamgrandin D **(373)**	MIC = 1.57–3.13 µg/mL (*S. aureus*, MRSA)	
*A. grandifolia*	Leaves and Root	2α-ethoxy-2,3-*seco*tirucalla-2,29-epoxy-7-ene-23-oxo-3-oic acid **(52)**	MIC = 1.56 µg/mL (*S. aureus*); MIC = 25 µg/mL (*P. aeruginosa*); Insecticidal (*Artemia salina*) = 79.1%	[31]
		(23*E*)-2α-hydroxytirucall-7,23,25-triene-3-one **(53)**	MIC = 25 µg/mL (*S. aureus*); MIC = 25 µg/mL (*P. aeruginosa*); Insecticidal (*A. salina*) = 60.6%	
		2,3-*seco*tirucalla-2,3;2,29-diepoxy-7-ene-3,23-dione **(54)**	MIC = 25 µg/mL (*S. aureus*); MIC = 50 µg/mL (*P. aeruginosa*); Insecticidal (*A. salina*) = 26.0%	
*Swietenia humilis* Zucc	Seeds	Melianone **(263)**	IC_50_ ≅ 0.1 µM (antifungal, hyphal growth assay)	[43]
*Walsura trichostemon*	Roots	3-epimesendanin S 12-acetate **(62)**	MIC = 16–128 µg/mL (*Bacillus cereus, Bacillus subtilis*)	[47]
		3-epimesendanin S **(63)**	MIC = 16 µg/mL (*B. cereus*); MIC = 64 µg/mL (*B. subtilis*)	
*M. toosendan*	Stem Bark	Meliasenin G **(64)**	MIC = 64 µg/mL (*B. cereus, P. aeruginosa*); MIC = 128 µg/mL (*Escherichia coli*)	[48]
*A. grandifolia*	Leaves and Twigs	2α-ethoxy-2,3 *seco*tirucalla-2,29-epoxy-7-ene-23-oxo-3-oic acid **(120)**	Moderate activity: MIC = 12.5 mg/mL (Gram-positive bacteria: *S. aureus, B. subtilis*)	[99]
*D. lukii*	Stem Bark	3β-hydroxytirucalla-7,24-diene-6,23-dione **(100)**	MIC = 0.17–1.69 mM; 0.19–2.31 mM (reported ranges)	[59]
		3β-hydroxytirucalla-7,24-dien-23-one **(101)**	MIC = 0.17–1.69 mM; 0.19–2.31 mM (reported ranges)	
		3β,26-dihydroxytirucalla-7,24-diene-6,23-dione **(102)**	MIC = 0.17–1.69 mM; 0.19–2.31 mM (reported ranges)	
		(23*Z*)-3β,26-dihydroxytirucalla-7,23-diene **(370)**	MIC = 0.17–1.69 mM; 0.19–2.31 mM (reported ranges)	
		Methyl 6-oxomasticadienolate **(103)**	MIC = 0.17–1.69 mM; 0.19–2.31 mM (reported ranges)	
		Dysoxylumstatin A **(226)**	MIC = 0.17–1.69 mM; 0.19–2.31 mM (reported ranges)	
		Dysoxylumstatin B **(306)**	MIC = 0.17–1.69 mM; 0.19–2.31 mM (reported ranges)	
		Dubione B **(227)**	MIC = 1.57–3.13 µg/mL (*S. aureus*, MRSA)

**Table 8:** Anti-inflammatory activity of tirucallane compounds

Species	Parts	Compounds	Bioactivities	References
*Dysoxylum binectariferum*	Stem bark	( +)-21*R*,23*R*-epoxy-21α-methoxy-24*S*,25-dihydro xyapotirucall-7-en-3-one **(161)**	Inhibition COX-1 = 95.3%	[71]
		( +)-21*R*,23*R*-epoxy-21α methoxy-25-hydroxyapotirucall-7-en-3,24-dione **(162)**	Inhibition COX-1 = 94.7%	[71]
		( +) 21*R*,23*R*-epoxy-21α,25-dimethoxyapotirucall-7-en-3,24 dione **(252)**	Inhibition COX-1 = 94.2–94.4%	[71]
		( +)-21*R*,23*R*-epoxy-21α-methoxy-24,25-dihy droxyapotirucall-7-en-3-one **(163)**	Inhibition COX-1 = 88.1%	[71]
*A. grandifolia*	Stem bark	Methyl 3-oxotirucalla-7,24-dien-21-oate **(331)**	IC_50_ = > 100 µM (NO), > 100 µM (TNF-α)	[40]
		Methyl 3β-hydroxy-tirucalla-7,24-dien-21-oate **(18)**	IC_50_ = 55.8 µM (NO), > 100 µM (TNF-α)	[40]
		Methyl (3β,5β,9β)-3,5-dihydroxytirucalla-7,24-dien-21-oate **(19)**	IC_50_ = 25.1 µM (NO), > 100 µM (TNF-α)	[40]
		(3*R*,5*R*,9*R*,10*S*,13*S*,14*R*,17*S*)-9-hydroxy-10,13-dimethyl-3-oxo-2,3,4,5,6,7,8,9,11,12,14,15,16,17-tetradecahydro-1H-cyclopenta[a]phenanthrene-17-carboxylate **(20)**	IC_50_ = 23.5 µM (NO), 57.0 µM (TNF-α)	[40]
*A. grandifolia*	Stem bark	(3*R*,5*R*,9*R*,10*S*,13*S*,14*R*,17*S*)-3-oxo-10,13-dimethyl-2,3,4,5,6,7,8,9,11,12,14,15,16,17-tetradecahydro-1H-cyclopenta[a]phenanthrene-17-carboxylate **(259)**	IC_50_ = 94.5 µM (NO), > 100 µM (TNF-α)	[53]
		3-oxo-urs-12-en-28-oic acid 17β-*O*-[3-(1-ethoxy-1-hydroxy-2-methyl)propionyl] ester **(21)**	IC_50_ = > 100 µM (NO), > 100 µM (TNF-α)	[53]
		3-oxo-urs-12-en-28-oic acid 17β-*O*-[3-(hydroxy-2-methylpropanoyl)oxy]propyl ether (dimethoxy substituted) **(22)**	IC_50_ = 27.8 µM (NO), 44.3 µM (TNF-α)	[53]
		3-oxo-urs-12-en-28-oic acid 17β-*O*-[2-(1-hydroxy-2-methylpropanoyl)oxy]propyl ester **(23)**	IC_50_ = 42.2 µM (NO), > 100 µM (TNF-α)	[53]
		3-oxo-urs-12-en-28-oic acid 17β-*O*-[2-(1-hydroxy-2-methylpropoxy)propionyl] ester **(24)**	IC_50_ 34.8 µM (NO), > 100 µM (TNF-α)	[53]
		3β-hydroxy-urs-12-en-28-oic acid 17β-[4,4-dimethoxy-2,2-dimethyl-tetrahydrofuran-3-yl] ester **(260)**	IC_50_ = > 100 µM (NO), > 100 µM (TNF-α)	[53]
		3-oxo-urs-12-en-28-oic acid 17β-(2-oxotetrahydrofuran-3-yl) ester **(322)**	IC_50_ = 19.6 µM (NO), 83.2 µM (TNF-α)	[53]
		3β-hydroxy-urs-12-en-28-oic acid 17β-(2-hydroxy-2-oxotetrahydrofuran-3-yl) ester **(323)**	IC_50_ = 62.6 µM (NO), > 100 µM (TNF-α)	[53]
		3-oxo-urs-12-en-28-oic acid 17β-(3-methoxy-2-oxotetrahydrofuran-3-yl) ester **(324)**	IC_50_ = > 100 µM (NO), > 100 µM (TNF-α)	[53]
		3β-hydroxy-urs-12-en-28-oic acid-17β-[1-(2-methylpropanoyl)pyrrolidin-2-yl] ester **(355)**	IC_50_ = 35.0 µM (NO), > 100 µM (TNF-α)	[53]
		3-oxo-urs-12-en-28-oic acid-17β-[4,4-dimethoxy-5,5-dimethyl-γ-lactone] **(252)**	IC_50_ = 12.0 µM (NO), > 100 µM (TNF-α)	[53]
		3β,19-dihydroxy-urs-12-en-28-oic acid-23-al **(325)**	IC_50_ = 90.0 µM (NO), > 100 µM (TNF-α)	[53]
		3β-hydroxy-urs-12-en-28,23-olide **(327)**	IC_50_ = 45.6 µM (NO), > 100 µM (TNF-α)	[53]
*Dysoxylum hainanense*	Stem bark	Dysoxyhaine C **(264)**	Cox-1: < 0%; Cox-2: 21.1%; DPPH IC_50_ = 99.7 ± 3 µM; ABTS⁺ IC_50_ = 59.2 ± 3 µM	[76]
		Dysoxyhaine D **(32)**	Cox-1: < 0%; Cox-2: 24.3%; IC_50_ = 94.1 ± 3 µM; ABTS⁺ IC_50_ = 54.6 ± 3 µM	
*Chukrasia tabularis*	Fruits	(24*S*)-7α,8α-epoxy-24-hydroxy-21α,25-dimetoxy-19(10 → 9β)*abeo*-tirucallane-5(10)-en-3-on **(317)**	IC_50_ = 75.98 ± 2.12 µM	[55]
		(24*R*)-7α,8α-epoxy-24-hydroxy-21α-metoxy-19(10 → 9β)*abeo*-tirucallane-5(10),25-dien-3-on **(318)**	IC_50_ = 24.86 ± 1.16 µM

**Table 9:** Other activity of tirucallane compounds

Species	Parts	Compounds	Bioactivities	References
*Entandrophragma congoënse*	Root	*Seco-*tiaminic acids B **(332)**	IC_50_ = 5.08 µM (P. falciparum NF54)	[32]
		*Seco-*tiaminic acids C **(333)**	IC_50_ = 6.22 µM(P. falciparum NF54)	
		Piscidinol A **(1)**	IC_50_ = 8.7 µM (P. falciparum NF54)	
		Hispidol A **(2)**	IC_50_ = 2.45 μM (P. falciparum NF54)	
*Chisocheton cumingianus* subsp.* Balansae*	Twigs	Phellochin **(66)**	IC_50_ = 6.1 µM (α-glucosidase)	[49]
*M. toosendan*	Fruits	(3*S*,5*R*,9*R*,10*R*,12*R*,13*S*,14*R*,16*S*,17*S*,20*S*)-3,12-dihydroxy-tirucallane-7,24-diene-21-methyl ester **(117)**	IC_50_ = 57.5 ± 1.5 µM (α-glucosidase)	
		(3*R*,5*R*,9*R*,10*R*,13*S*,14*S*,17*S*,20*S*,21*S*,23*R*,24*R*)-21-ethoxy-3,24,25-trihydroxy-21,23-epoxy-tirucalla-7-ene **(207)**	IC_50_ = 32.5 ± 0.9 µM (α-glucosidase)	
		(3*S*,5*R*,9*R*,10*R*,13*S*,14*S*,17*S*,20*R*,22*R*,23*S*,24*S*)-lanost-7-ene-3,23,24-triol-22,25-epoxy **(208)**	IC_50_ = 89.47 ± 5.03 µM (α-glucosidase)	
		Cinamodiol **(209)**	IC_50_ = 243.4 ± 10.3 µM (α-glucosidase)	
*M. toosendan*	Stem Bark	Meliasenin G **(64)**	IC_50_ = 6.1 µM (α-glucosidase)	
		Toosendine E **(118)**	IC_50_ = 57.5 ± 1.5 µM (α-glucosidase)	
*Entandrophragma angolense*	Bark	Entandrophin A **(210)**	IC_50_ = 32.5 ± 0.9 µM (α-glucosidase)	[63]
		Entandrophin B **(211)**	IC_50_ = 89.47 ± 5.03 µM (α-glucosidase)	
		Entandrophin C **(212)**	IC_50_ = 243.4 ± 10.3 µM (α-glucosidase)	
		Methyl angolensate **(343)**	IC_50_ = 6.1 µM (α-glucosidase)	
*X. granatum, X. moluccensis*	Seeds, stems and twigs	Xylocarpol A **(25)**	FXR activation, agonist at 10 µM	[22]
		Xylocarpol B **(26)**	FXR activation, agonist at 10 µM	
		Xylocarpol C **(27)**	FXR activation, agonist at 10 µM	
		Xylocarpol D **(182)**	FXR activation, agonist at 10 µM	
		Agallochol A **(28)**	FXR activation, agonist at 1 µM and 10 µM	
		Agallochol B **(29)**	FXR activation, agonist at 1 µM and 10 µM	
		Agallochol C **(30)**	FXR activation, agonist at 10 µM	
		Agallochol D **(31)**	FXR activation, agonist at 10 µM	
*M. azedarach*	Ripe fruit	21β-ethylmelianodiol **(193)**	PI = 0.47–1.00	[30]
		21α-methylmelianodiol **(191)**	*Myzus persicae*: PI = −0.22 ± 0.13 *Rhopalosiphum padi*: PI = 0.09 ± 0.10 *Spodoptera littoralis*: PI = 0.33 ± 0.20 *Epilachna paenulata*: PI = 0.08 ± 0.42	
*T. hispida*	Leaves	Sapelin A **(236)**	LC_50_: 200 μg/Ml 23,3%, 53,3% (*brassica oleracea* var. *capitate*)	[59]

### Cytotoxic activity of tirucallane compounds

Tirucallane-type triterpenoids shown in Table 6 show diverse cytotoxic profiles. Among these compounds, 21α-methoxy-24,25-dihydroxyapotirucall-7-en-3-one (**166**) demonstrated moderate inhibition of P-388 cells with IC_50_ values ranging from 3.3 to 9.9 mg/mL [72]. Leaves of *Trichilia hispida* produced hispidol B (**3**), which showed an IC_50_ value of 5.2 mg/mL against P-388 cells [33]. A broader spectrum of activity was observed among tirucallane lactones isolated from the stem bark of *Aphanamixis grandifolia*, with 3α-hydroxy-21a-methoxy-24,25,26,27-tetranortirucall-7-ene-23(21)-lactone (**321**) showing cytotoxicity evidenced by IC_50_ values of 42.2 µM (MCF-7), 37.6 µM (HeLa), 31.4 µM (HepG2), 26.1 µM (SGC-7901), and 24.2 µM (BGC-823). The closely related compound, namely 3-oxo-24,25,26,27-tetranortirucall-7-ene-23(21)-lactone (**125**), showed moderate potency with IC_50_ values of 67.1 µM (MCF-7), 24.3 µM (HeLa), 32.6 µM (HepG2), 21.3 µM (SGC-7901), and 12.8 µM (BGC-823). Meanwhile, 3-oxo-urs-12-en-28-oic acid 17β-(2-oxotetrahydrofuran-3-yl) ester (**322**) demonstrated weaker cytotoxicity with IC_50_ values of 166.5 µM (MCF-7), 95.5 µM (HeLa), 91.2 µM (HepG2), 70.9 µM (SGC-7901), and 154.4 µM (BGC-823). The regioisomer, 3β-hydroxy-urs-12-en-28-oic acid 17β-(2-hydroxy-2-oxotetrahydrofuran-3-yl) ester (**323**), showed moderate inhibition with IC_50_ values of 76.2 µM (MCF-7), 52.8 µM (HeLa), 71.8 µM (HepG2), 71.9 µM (SGC-7901), and 41.7 µM (BGC-823). Another derivative, 3-oxo-urs-12-en-28-oic acid 17β-(3-methoxy-2-oxotetrahydrofuran-3-yl) ester (**324**), demonstrated cytotoxicity of 50.2 µM (MCF-7), 76.2 µM (HeLa), 58.3 µM (HepG2), 108.0 µM (SGC-7901), and 126.6 µM (BGC-823). Additional analogues, including 3β,19-dihydroxy-urs-12-en-28-oic acid-23-al (**325**) and 3β-hydroxy-urs-12-en-28,23-olide (**326**), had IC_50_ values ranging from 57.2 to 97.6 µM (MCF-7), 25.7 to 64.6 µM (HeLa), 32.7 to 38.8 µM (HepG2), 15.7 to 39.1 µM (SGC-7901), and 33.0 to 38.8 µM (BGC-823) [53]. Overall, comparison of these tirucallane-derived metabolites shows the critical influence of functional-group variation on cytotoxic profiles. The presence of a 3α-hydroxyl group combined with a 21-alkoxy substituent and a 23(21)-lactone moiety, as exemplified by compound **321**, is associated with enhanced cytotoxicity relative to the 3-oxo analogue (**125**), indicating the importance of hydroxylation at C-3. In contrast, the introduction of carbonyl groups at C-3, modification of the lactone or aldehyde functionalities, and variation in methoxy or hydroxy substitution in tetrahydrofuran side chain (**322–326**) led to attenuated or moderate activity. These observations collectively suggest that oxygenated functionalities at C-3 and C-21, together with lactonization or aldehyde formation, play a decisive role in modulating the cytotoxic potential, while excessive oxidation or side-chain modification may reduce activity.

Additional tirucallane derivatives from *Dysoxylum lenticellatum* and *Aphanamixis grandifolia* further show significant cytotoxic profiles. From *D. lenticellatum*, dysolenticin A (**365**) demonstrated moderate activity toward MCF-7 cells with an IC_50_ value of 53.3 µM [42]. However, dysolenticin C (**167**) had weak activity, presenting IC_50_ values greater than 100 µM against MCF-7 but improved potency toward HeLa cells with an IC_50_ of 15.3 µM. Dysolenticin D (**168**) showed moderate inhibition with IC₅₀ values of 29.9 µM in HeLa and 10.3 µM in MCF-7 cells, indicating cell-type selectivity [42]. These differences in activity are primarily attributed to functional-group variations at C-3, particularly hydroxy–oxo interconversion, together with oxidation or unsaturation at C-7/C-8 in the B ring, which tends to enhance cytotoxic effects, specifically toward HeLa cells. Oxidative modifications at C-17 and the side chain further modulate selectivity. Therefore, oxygenation at C-3, structural features in the B ring, and C-17 side-chain modifications were identified as the key determinants influencing the cytotoxic behavior of dysolenticin-type triterpenoids [42].

Complex lanostane-type tirucallane derivatives isolated from the stem bark of *Aphanamixis grandifolia* further showed diverse cytotoxicity profiles. One epoxy-lanostane derivative (**170**) demonstrated moderate inhibition with IC_50_ values of 53.3 µM (MCF-7) and 21.4 µM (HeLa). Two additional structurally related compounds (**257**) showed weak activity toward MCF-7 cells (IC_50_ > 100 µM), but each retained moderate potency toward HeLa cells with IC_50_ values of 15.3 µM. Another analogue (**258**) had stronger activity, with IC_50_ values of 10.3 µM (MCF-7) and 29.9 µM (HeLa) [73]. A comparative assessment of compounds 144, 219, and 220 showed that cytotoxic potency is largely determined by the nature and positional arrangement of oxygenated functional groups. In compound **170**, the coexistence of a 21,23-epoxy unit with carbonyl functionalities at C-3 and C-21 is associated with moderate activity. Modification at C-3 plays a critical role, as the 3-oxo analogue (**258**) shows higher cytotoxicity than the corresponding 3-hydroxy derivative (**257**). Aphanamgrandnin A (**371**), isolated from the stems of *Aphanamixis grandifolia*, demonstrated relatively weak cytotoxicity with an IC_50_ value of 160.74 μM against HeLa cells [34]. In contrast, the stem bark-derived compound indicalilacol B (**206**) from *Chisocheton pentandrus* showed moderate cytotoxicity, with IC_50_ values of 15.0 μg/mL (KB), 16.1 μg/mL (KB-C2), 19.0 μg/mL (KB-C2 + colchicine), and 15.7 μg/mL (MCF-7) [79]. Several metabolites from the fruits of *Azadirachta indica* had varying activities. More specifically, indicaliatocol D (**12**) showed cytotoxicity toward KB-C2 + colchicine and MCF-7 cells with IC_50_ values of 4.60 and 9.04 μg/mL, respectively. Meliasenin S (**175)** was inactive toward the tested cell lines but indicated MDR-reversal behavior, with an IC₅₀ of 6.48 μg/mL in the presence of colchicine. Meliantriol (**176**) showed moderate to strong activity, with IC_50_ values of 25.7 μg/mL (KB), 15.9 μg/mL (KB-C2), 20.9 μg/mL (KB-C2 + colchicine), and 0.27 μg/mL (MCF-7). Meliasenin T (**177**) also presented comparable cytotoxic effects, yielding IC_50_ values of 17.1 μg/mL (KB), 15.6 μg/mL (KB-C2), 6.67 μg/mL (KB-C2 + colchicine), and 16.0 μg/mL (MCF-7) [36]. Hydroxylation at C-3 is central to cytotoxic potency, and the effect is further enhanced by additional hydroxyl substituents at C-7, C-12, and the side-chain position C-21, as exemplified by **176** and **177**. In contrast, oxygenation at C-21 or the side chain in the absence of a C-3 hydroxyl group tends to promote MDR-reversal activity rather than direct cytotoxicity, as observed for **177** [36].

Compounds isolated from *Entandrophragma congoënse* further contributed to the cytotoxic profile of Meliaceae family. The stem-bark metabolite, namely (20*S*)-3-oxo-tirucalla-25-nor-7-en-24-oic acid (**329**), showed moderate cytotoxicity across several cancer cell lines, with IC_50_ values of 7.8 μM (HL-60), 15.2 μM (A549), 18.4 μM (MCF-7), and 12.1 μM (HepG2). Piscidinol A (**1**), obtained from the roots, had a more complex activity pattern, with IC_50_ values of 11.6 μM (HL-60), 14.5 μM (A549), 17.9 μM (MCF-7), and 10.7 μM (HepG2). Additional assays reported stronger activities in other panels, including 3.3 μg/mL (HL-60), 8.5 μg/mL (KB), and 9.9 μg/mL (HeLa). Further cytotoxic responses included IC_50_ values of 1.16 μM (A549), 3.01 μM (BGC-823), 6.9 μM (MKN-28), and > 10 μM (A549), as well as 8.5 μM toward MCF-7. Meanwhile, hispidol B **(3**) from leaves of *Trichilia hispida* showed weak to moderate cytotoxicity, with IC_50_ values > 40 μM (HL-60), 32.4 μM (A549), 35.7 μM (MCF-7), and 28.6 μM (HepG2) [32]. These observations emphasize the importance of oxidation at C-3 and appropriate side-chain functionalization, as a C-3 carbonyl group combined with a well-oxygenated side chain appears to favor enhanced cytotoxicity, while reduced oxidation or loss of key oxygenated functionalities results in diminished activity.

Cytotoxicity assessments of additional Meliaceae-derived metabolites demonstrated considerable variability in potency across cancer cell lines. From the stem bark of *Toona sinensis*, triterpenoid 4,4,14-trimethyl-3-oxo-24-nor-5α,13α,14β,17α,20S-chol-7-en-23-oic acid (**331**) showed moderate cytotoxicity, with IC_50_ values of 9.4 μM (HL-60), 13.5 μM (A549), 16.7 μM (MCF-7), and 8.9 μM (HepG2). Bourjotinolone B (**17**) from the same species also had comparable activity, with IC_50_ values of 8.1 μM (HL-60), 11.3 μM (A549), 14.8 μM (MCF-7), and 9.6 μM (HepG2) [52]. Furthermore, several compounds from *Guarea guidonia* have been evaluated, including the *seco-*triterpenoid guareoic acid B (**335**), which showed IC_50_ values of 39 μM (Jurkat), 55 μM (HeLa), 75 μM (MCF-7), and > 100 μM (PBMC), indicating low to moderate cytotoxicity. Flindssone (**183**) had slightly stronger activity, with IC_50_ values of 25 μM (Jurkat), 27 μM (HeLa), and 50 μM (MCF-7), while Picroquassin E (**184**) showed weaker effects with IC_50_ values of 88 μM (Jurkat) and greater than 100 μM in both HeLa and MCF-7 cells [52].

Stems of *Aphanamixis grandifolia* yielded leucophyllone (**11**), which was mostly inactive, showing IC_50_ values above 30 μM across all tested lines, including A549, SK-OV-3, SK-MEL-2, and XF498 [34]. In contrast, the bark produced toonaiciliatavarine E (**74**) with a significant cytotoxic profile indicated by IC_50_ values of 17.1 ± 1.4 μM (MCF-7), 22.6 ± 3.8 μM (MCF-7/ADR), 10.2 ± 1.1 μM (KB), 32.1 ± 2.3 μM (KB/VCR), 19.4 ± 1.9 μM (SMMC-7721), and 11.2 ± 0.9 μM (K562). Leaves of *Trichilia hispida* produced hispidol B (**3**), which showed IC_50_ values of 6.2 μg/mL (HL-60), 7.4 μg/mL (KB), and 8.1 μg/mL (HeLa), while hispidol A (**2**) demonstrated slightly weaker cytotoxicity, with IC_50_ values of 3.8 μg/mL (HL-60), 5.9 μg/mL (KB), and 7.1 μg/mL (HeLa) [33]. The difference in cytotoxic activity between hispidol A (**2**) and B (**3**) can be primarily attributed to variations in oxygenated functional groups, particularly at C-3 and the side chain. The roots of *Cedrela fissilis* produced nilocetin (**230**), which showed consistent cytotoxicity with IC_50_ values of 8.1 μg/mL (HL-60), 9.3 μg/mL (KB), and > 10 μg/mL (HeLa) [25]. Furthermore, the leaves and twigs of *Toona ciliata* var. *ciliata* yielded Toonaacilin L (**35**), with IC_50_ values of 25 μM (MCF-7), 20 μM (HeLa), and 14 μM (K562) [24]. Finally, *Cedrela odorata* produced Altissimanin A (**267**), which showed relatively potent cytotoxicity with IC_50_ values of 15.2 μM (MCF-7) and 11.0 μM (K562) [25]. Several additional cytotoxic metabolites have been reported from *Toona* and related Meliaceae species. From the stem bark and aerial parts of *Toona ciliata* and *Toona ciliata* var. *henryi*, 3-episapelin A (**217**) showed moderate cytotoxicity, with IC_50_ values of 50 μM (MCF-7), 43 μM (HepG2), and 38 μM (HeLa). Another metabolite, toonaiciliatavarin D (**216**), isolated from the same plant parts, had relatively weak activity, with IC_50_ values exceeding 50 μM against MCF-7, KB, and MCF-7/ADR cells, as well as KB/VCR. However, a moderate response was observed toward SMMC-7721 and K562 cells, with IC_50_ values of 39.5 ± 2.0 μM and 43.1 ± 2.1 μM, respectively. Toonaaciliatin K (**192**), isolated from the leaves and twigs of *Toona ciliata*, also showed moderate cytotoxicity, with IC_50_ values of 24.6 μM (MCF-7), 27.1 μM (KB), and 28.4 μM (K562) [25].

Hispidone (**218),** obtained from the bark, leaves, and twigs of *Toona ciliata* as well as the variety *henryi*, demonstrated more potent cytotoxicity, with IC_50_ values of 6.5 μg/mL (HL-60), 8.2 μg/mL (KB), and 9.0 μg/mL (HeLa). A series of dymacrin-type compounds isolated from the stem bark of *Dysoxylum macranthum* also demonstrated significant activity. Dymacrin B (**41**), C (**42**), H (**268**), and J (**270**) showed cytotoxic effects against KB cells, with IC_50_ values of 5.6 μg/mL, 5.0 μg/mL, 8.3 μg/mL, and 1.0 μg/mL, respectively, indicating that dymacrin J (**270**) is the most potent member of the group [26]. In addition, fruits of *Melia azedarach* yielded the limonoid 3-β-tigloylmelianol (**271**), which showed moderate cytotoxicity with an IC_50_ of 91.8 μg/mL (A549) and a CC_50_ value of 64.7 μg/mL in the same cell line [27]. Seeds of *Swietenia humilis* also produced melianone (**263**), a compound with comparatively stronger activity, showing an IC_50_ value of 3.6 μg/mL (A549) [43]. From *Dysoxylum variabile* stem bark, tirucalla-7,24-diene-3β,23-diol (**49**) demonstrated moderate cytotoxicity with an IC_50_ of 10.0 µg/mL against KB cells. A series of dyvariabilins E–H **273–275**,** 266** showed comparable activity, with IC_50_ values ranging from 8.4–10.1 µg/mL in the same cell line, while Nilotictin (**276**) had similar potency (IC_50_ = 9.2 µg/mL) [28]. The similar cytotoxic potencies of dyvariabilins E–H (**273–275**, **266**), and nilotictin (**276**) indicate that activity is mainly driven by C-3 oxygenation together with oxygenated side-chain functionalities.

Ripe fruit of *Melia azedarach* yielded two limonoid derivatives, namely 21α-methylimelianodiol (**191**) and 21β-methylimelianodiol (**197**), which demonstrated weak cytotoxicity with IC_50_ value of more than 10 µM toward A549 and BGC-823 cells. However, the activity varied across additional cell lines with IC_50_ values of 92.8 µM (HepG2), 47.6 µM (K562), 38.4 µM (SGC7901), and 17.9 µM (HL60), suggesting selective and cell-dependent effects. Moderate and cell-dependent activity was observed for **175** against other cancer cell lines, with IC_50_ values of 27.9 µM (HepG2), 19.7 µM (K562), 27.9 µM (SGC-7901), and 16.8 µM (HL-60), indicating selective cytotoxic effects [29]. The cytotoxic effects are primarily determined by the substitution at C-21, which affects both the potency and selectivity across different cell lines. β-oriented methyl group at C-21, together with the hydroxyl groups on the core structure, supports the moderate activity, while the absence of further oxidized groups restricts overall cytotoxic strength. *Chisocheton pentandrus* produced melianodiol (**185**), which showed only low activity with an IC_50_ value of more than 10 µM across MKN-28, A-549, and MCF-7 cell lines [79]. From *Toona ciliata* var. *pubescens* stem bark, 24*S*,25-dihydroxytirucall-7-en-3-one (**110**) demonstrated modest cytotoxicity with IC_50_ of 9.1 µM in MKN-28 and 10.2 µM in A549. Nilotictin (**276**) from this species also showed moderate to weak cytotoxicity with IC_50_ value above 50 µM in HepG2 and less than 50 µM for Hep3B [60]. Finally, dihydronotictin (**108**) isolated from the same plant had an IC_50_ of approximately 8.2 µM toward HT-1080, representing one of the strongest activities among the compounds reported [60].

A diverse set of tirucallane- and apotirucallane-type triterpenoids has been isolated from various species in Meliaceae family, many of which show measurable cytotoxicity. From *the* ripe fruit, several compounds showed only weak activity. Meliasenin S (**175**) had no significant activity, with IC_50_ values more than 100 µM across HepG2, K562, SGC7901, and HL60 cell lines, while meliasenin T (**177**) demonstrated moderate cytotoxicity, with IC_50_ values of 31.7 µM (HepG2), 13.7 µM (K562), 29.6 µM (SGC7901), and 6.3 µM (HL60), indicating preferential activity against HL60 cells. Derivatives including 21β,25-dimethylmeliandiol (**199**) and 21,25-dimethylmelianodiol (**200**) were generally weak, as indicated by IC_50_ more 100 µM in HepG2 and 25–90 µM across K562, SGC7901, and HL60. Additional tirucallane derivatives from *M. azedarach* showed moderate activity. The epoxy–methoxy compound (21*R*,23*R*)-epoxy-24-hydroxy-21β-methoxytirucall-7,25-dien-3-one (**201**) demonstrated IC_50_ values ranging from 10.8 to 49.4 µM across four cancer lines. Methoxytirucall-7,25-dien-3-one (**202**) and tirucall-7,25-dien-3-one (**204**) also showed similar profiles, with IC_50_ values between 20.9 and 81.7 µM for HepG2, K562, SGC7901 and HL60. The mixture of 21-oxo-melianodiol, 14,21-oxo-meliantriol 15 toosendanic acids A (**60**) demonstrated cytotoxicity primarily against HL60 cells with IC_50_ of 52.4 µM. Additional metabolites from *Melia azedarach* fruit included methyl kulonate (**47**) with IC_50_ values of 81.1 µM for HepG2 and 71.3 µM for HL60 [29]. Meliastatin (**61**) showed comparable weak activity with IC_50_ of 62.9 µM HepG2 and 18.0 µM HL60, although activity against K562 and SGC7901 was not assessed [46]. Therefore, the presence and positioning of oxygenated groups such as hydroxyl, methoxy, and epoxy moieties at C-21, C-23, and C-25 play a critical role in modulating both the strength and selectivity of cytotoxic effects in tirucallane and apotirucallane-type triterpenoids from *Melia azedarach* [10, 27].

Compounds from *Aphanamixis grandifolia* stem bark showed moderate cytotoxicity. The trihydroxy–dimethoxy triterpenoid 3α,24β,25-trihydroxy-21,21-dimethoxy-23-oxotirucall-7-ene (**72**) had IC_50_ values of 11.2 µM for NCI-H460 and 19.8 µM for HeLa. Additionally, the acetoxy–methoxy lactone derivative 3α-acetoxy-21β-methoxy-24,25,26,27-tetranortirucall-7-ene-23(21)-lactone (**281**) showed similar activity, with IC_50_ values of 38.9 µM (NCI-H460) and 51.0 µM (HeLa). From *Melia toosendan*, triterpenoids 3β,16β-hydroxytirucalla-7,24(25)-dien-21,23-olide (**282**) and 3β,16β-hydroxytirucalla-7,24(25)-dien-6-oxo-21,23-olide (**283**) demonstrated cytotoxicity in the low micromolar range [99]. Compound **250** had IC_50_ values of 4.3–5.7 µg/mL across A549, SK-OV-3, SK-MEL-2, and HCT15 cell lines, while compound **251** had IC_50_ values of 3.2–5.0 µg/mL across the same panel, indicating strong and broad cytotoxic potential [99].

Several laxiracemosins isolated from *Dysoxylum laxiracemosum* bark demonstrated relatively potent activity. More soecifically, laxiracemosin A (**357**) showed IC_50_ values of 3.1 µM for HL60 and 5.4–9.5 µM across A549, MCF-7, SW480, and SMMC-7721. Laxiracemosin B (**358**) was slightly weaker, while laxiracemosin D (**359)** demonstrated IC_50_ value of 6.8 µM for HL60 and more than 20 µM in A549 and MCF-7. Another member, laxiracemosin E (**360**), also showed moderate cytotoxicity. Laxiracemosin F (**361**) demonstrated selective activity toward A549 and MCF-7 with IC_50_ value of 22.9 µM and 20.2 µM, respectively. However, the activity was low toward HL60 and SW480. Cytotoxic effects of these triterpenoids are largely determined by both the presence and specific placement of oxygenated functional groups. Hydroxylation at positions C-3, C-16, C-24, C-25, together with methoxy and acetoxy substitutions at C-21, and the formation of a lactone ring between C-21, C-23.

Other tirucallane-type compounds showed variable activity across species. *Azadirachta indica* leaves yielded a highly potent cytotoxic agent, 24,25-epoxy-3β-hydroxy-20-oxo-7-tirucallene (**315**), with an IC_50_ of 0.8 ± 0.1 µM. *Melia toosendan* fruits yielded 22,23,24,25-diepoxy-3β-hydroxy-7-tirucallene (**316**) with IC_50_ of 2.2 ± 0.2 µM and a related 24,25-epoxy derivative (**277**) with value of IC_50_ of 1.9 ± 1.3 µM [23]. The exceptionally strong cytotoxic activity of these tirucallane-type compounds is largely determined by oxygenated functional groups, particularly the presence of epoxide moieties at C-24/C-25 or C-22/C-23, combined with a β-oriented hydroxyl at C-3 and a carbonyl group at C-20. In compound **320,** the 24,25-epoxy group, C-3 hydroxy, and C-20 ketone possibly act synergistically to confer the highest potency with IC_50_ value of 0.8 µM.

From *Toona ciliata* bark, odoratone (**293**) showed moderate cytotoxicity with IC_50_ of 4.5 ± 1.1 µM [54]. Additional metabolites included meliasenin E (**111**) from *M. azedarach*, with cytotoxicity of 00.65 µM (HL60) and 2.8 µM (RPMI 1788) [61]. *Seco-*tirukalana derivative (**349**) from *Chukrasia tabularis* fruit had IC_50_ values of 10.45 µM for K562 and 20–35 µM across HeLa, BEL-7402, SGC7901, and A549 [42]. Toosendine A (**285**) from *Munronia delavayi* showed cytotoxicity, with IC_50_ value of 12.63 ± 0.28 µM (U2OS). Other members, toosendine D (**288**) and E (**118**) showed similar profiles, with cytotoxic IC_50_ values ranging from 21 µM to 50 µM [82]. Furthermore, *Dysoxylum macranthum* stem bark yielded dysomollide A (**39**) with IC_50_ value of 5.6–8.3 µg/mL in KB cells, while *Chisocheton patens* produced chisopaten A (**68**) with IC_50_ value of 4.33 ± 0.009 µM in MCF-7, indicating strong activity. *Chisocheton lasiocarpus* yielded chisocarpene A (**69**) with moderate activity as indicated by IC_50_ value of 26.56 ± 1.01 µM [82]. Finally, 3β,16-dihydroxy-25-hydroperoxytirucalla-7,23(24)-dien-6-oxo acid (**282**) from *Melia toosendan* bark, demonstrated selective cytotoxicity with IC_50_ values of 14.2–15.3 µM across A549, SK-OV-3, SK-MEL-2, and HCT15 [100]. Among tirucallane-type triterpenoids, 24,25-epoxy-3β-hydroxy-20-oxo-7-tirucallene (**315**) from *Azadirachta indica* was identified to be exceptionally potent, with an IC_50_ of 0.8 ± 0.1 µM, indicating strong potential as a cytotoxic agent.

### Antimicrobial activity of tirucallane compound

A diverse range of tiruucalane compounds isolated from several Meliaceae species demonstrated significant antimicrobial and related bioactivities. In *Aphanamixis grandifolia*, the stem-derived compounds, aphanamgrandin C(**372**) and D (**373**), showed strong antibacterial activity against *Staphylococcus aureus* and methicillin-resistant *S. aureus* (MRSA), with MIC values of 1.57–3.13 µg/mL [34]. Additional *seco-*tirucallane derivatives **52–54** obtained from leaves and roots demonstrated moderate antibacterial activity against *S. aureus* and *Pseudomonas aeruginosa* with MIC value of 1.56–50 µg/mL, alongside variable insecticidal effects toward *Artemia salina* (26–79%) [31]. Furthermore, the seeds of *Swietenia humilis* yielded melianone (**263**), which showed potent antifungal activity with IC_50_ of approximately 0.1 µM [43]. From the roots of *Walsura trichostemon*, 3-epimesendanins S 12-acetate analogue (**62**) and **63** presented moderate antibacterial properties, with MIC values ranging from 16–128 µg/mL against *Bacillus cereus* and *B. subtilis* [47]. The stem bark of *Melia toosendan* produced meliassenin G (**64**), which showed relatively weak antibacterial activity with MIC values of 64–128 µg/mL in *Bacillus cereus, Pseudomonas aerugino,s* and *Escherichia coli* [48]. Additional compound (**120)** from *A. grandifolia* leaves and twigs indicated only moderate activity against Gram-positive bacteria, with MIC values of 12.5 mg/mL in *S. aureus* and *B. subtilis* [99]. In *Dysoxylum lukii*, several tirucallane-type triterpenoids and related derivatives have been evaluated for the antimicrobial potential. Compounds including 3β-hydroxytirucalla-7,24-diene-6,23-dione (**100**), 3β-hydroxytirucalla-7,24-dien-23-one (**101**), 3β,26-dihydroxytirucalla-7,24-diene-6,23-dione (**102**), (23*Z*)-3β,26-dihydroxytirucalla-7,23-diene (**370**), methyl 6-oxomasticadienolate (**103**), dysoxylumstatin A (**226**), and dysoxylumstatin B (**306**) had MIC values in the range of 0.17–1.69 mM, consistent with previously reported values of 0.19–2.31 mM [59]. In contrast, dubione B (**227**) showed selective activity against *Staphylococcus aureus* and methicillin-resistant *S. aureus* (MRSA), with MIC values of 1.57–3.13 µg/mL, indicating significant antibacterial potential. These results show that subtle variations in hydroxylation and ketone functionalities in tirucallane backbones can influence antimicrobial efficacy [59].

### Anti-inflammation activity of tirucallane compound

A diverse set of tirucallane derivatives isolated from several Meliaceae species demonstrated significant anti-inflammatory activities, primarily through cyclooxygenase inhibition as well as suppression of nitric oxide (NO) and TNF-α production. Stem bark extracts of *Dysoxylum binectariferum* yielded multiple epoxy-substituted apotirucallane triterpenoids, including ( +)-21*R*,23*R*-epoxy-21α-methoxy-24*S*,25-dihydroxyapotirucall-7-en-3-one (**161**) and ( +)-21*R*,23*R*-epoxy-21α-methoxy-25-hydroxyapotirucall-7-en-3,24-dione (**162**). Both compounds demonstrated strong COX-1 inhibition with activities exceeding 94–95% [71]. Additional analogues such as ( +)-21*R*,23*R*-epoxy-21α,25-dimethoxyapotirucall-7-en-3,24-dione (**252**) and related methoxy- or dimethoxy-substituted derivatives (**163**) showed COX-1 inhibition ranging from 88.1% to 94.4%, confirming a consistent anti-inflammatory trend in the structural class [71]. Epoxy moieties at C-21/C-23, hydroxyl groups at C-24 and C-25, along with methoxy substitutions at C-21, consistently enhance cyclooxygenase (COX-1) inhibition, as demonstrated by compounds **161**, **162**, **163**, and **252** [71].

Numerous tirucallane-type triterpenoids from the stem bark of *Aphanamixis grandifolia* showed significant inhibition of inflammatory mediators. Compounds such as methyl 3-oxotirucalla-7,24-dien-21-oate (**331**) and methyl 3β-hydroxytirucalla-7,24-dien-21-oate (**18**) had IC_50_ values above 100 μM against NO and TNF-α, while others demonstrated higher potency, including **19** with IC_50_ value of 23.5 μM for NO, 57.0 µM for TNF-α, as well as **259** with IC_50_ values of 94.5 µM and > 100 µM, respectively [40]. Several ursane-type triterpenoid esters, including **21–24, 260**, **322–327**, **355**, **260,** showed IC_50_ values ranging from 12.0 to 62.6 μM for NO inhibition, though most demonstrated weak TNF-α inhibition values of above 100 μM [53]. Substituted oxo-urs-12-en-28-oic acids containing furan or lactone moieties demonstrated moderate activity, with IC_50_ values between 12.0 and 49.8 μM, suggesting that esterification and heterocyclic substituents influence the anti-inflammatory response [53]. Two compounds, dysoxyhaine C (**264**) and D (**32**) from *Dysoxylum hainanense*, showed weak COX-1 and COX-2 inhibition value with less than 1% for COX-1, 21–24% for COX-2. However, the radical-scavenging properties were moderate, with DPPH IC_50_ values of 94–99 μM and ABTS IC_50_ values of 54–59 μM [76].

Fruit-derived phenolics from *Chukrasia tabularis* showed more significant activity. Compounds such as (24*S*)-7α,8α-epoxy-24-hydroxy-21α,25-dimethoxy-19(10 → 9β)*abeo*-tirukalana-5(10)-en-3-one (**317**) and the 25-dien analogue (**318**) demonstrated strong inhibition of lipoxygenase with IC_50_ value of 75.98 and 24.86 μM, respectively [55]. Collectively, the data indicate that phenolic and triterpenoid compounds from Meliaceae family possess significant anti-inflammatory potential, with the most potent activities connected to epoxy-substituted apotirucallane triterpenoids in *D. binectariferum* and selected ursane- or tirucallane-type esters in *A. grandifolia*. Structural features such as epoxy bridges, methoxy substitution, esterification patterns, and heterocyclic moieties appear to play a key role in modulating anti-inflammatory efficacy.

### Other activity of tirucallane compound

A wide range of tirucallane compounds isolated from various Meliaceae species have shown diverse biological activities, particularly antiparasitic, α-glucosidase inhibitory, and FXR-agonistic effects. The roots of *Entandrophragma congoënse* yielded *seco-*tiaminic acids B and C (**332–333**), piscidinol A (**1**), hispidol A (**2**), and phellochin (**66**), which demonstrated strong antiplasmodial effects against *Plasmodium falciparum* NF54, with IC_50_ values ranging from 2.45 to 8.7 μM [32, 49]. Similarly, the twigs of *Chisocheton cumingianus* subsp. *balansae* produced several tirucallane-type triterpenoids showing moderate α-glucosidase inhibition, including compounds **117**, **207–209,** with IC_50_ values of 57.5 ± 1.5 μM. The fruits of *Melia toosendan* contained structurally related triterpenoids demonstrating α-glucosidase inhibitory activity with IC_50_ value of 32.5 ± 0.9 μM to 89.47 ± 5.03 μM [49]. Meliasenin G (**64**) from the stem bark of *Melia toosendan* demonstrated strong α-glucosidase inhibition with an IC_50_ of 6.1 µM, while toosendine E (**118**) showed weaker activity with IC_50_ value of 57.5 ± 1.5 µM. These results indicate that subtle structural differences, such as hydroxylation or acetylation patterns, significantly impact potency. Similarly, bark-derived tirucallane derivatives from *Entandrophragma angolense* showed a range of α-glucosidase inhibition. Entandrophin A (**210**) and methyl angolensate (**343**) were the most active, with IC_50_ value of 6.1–32.5 µM, while entandrophins B (**211**) and C (**212**) showed moderate to weak effects with IC_50_ value of 89.5–243.4 µM. This suggests that side-chain oxidation and functional-group positioning at C-3 and C-21 are crucial for activity [63].

Tetranortriterpenoids from *Xylocarpus granatum* and *X. moluccensis*, including xylocarpols A–D (**26–28**, **182**) and agallochols A–D (**28**–**31**), acted as FXR agonists at 1–10 µM, demonstrating that epoxy and hydroxyl substituents in the side chain enhance receptor activation [22]. Ripe fruits of *Melia azedarach* also produced insecticidal tirucallane derivatives. The compound, 21β-ethylmelianodiol (**193**), showed a protein inhibition (PI) range of 0.47–1.00, while 21α-methylmelianodiol (**191**) had variable effects across insect species, with PI values from 0.22 to 0.33, underscoring the influence of C-21 methyl/ethyl orientation on insecticidal potency [30]. Leaves of *Trichilia hispida* yielded sapelin A (**236**), which showed moderate toxicity toward *Brassica oleracea* var. *capitata* with an LC_50_ of 200 µg/mL and inhibition percentages of 23.3–53.3%, suggesting that hydroxyl and ketone functionalities in the tetracyclic core contribute to pesticidal activity [59]. These data collectively indicate that the positions and types of oxygenated substituents, particularly hydroxyl, methoxy, acetoxy groups, and epoxides at C-3, C-21, as well as side chains, critically affect α-glucosidase inhibition, nuclear receptor activation, and insecticidal properties across Meliaceae triterpenoids.

## Conclusion

In conclusion, tirucallane-type triterpenoids from the Meliaceae family have been extensively reported between 1967 and 2025 across various plant parts, including the bark, stem bark, wood, roots, leaves, fruits, seeds, twigs, and aerial tissues. These metabolites are structurally diverse and classified into several groups, dominated by intact tirucallane derivatives, followed by *seco-*tirucallane, degraded tirucallane, heteroatom-containing, and highly rearranged tirucallane structures. The chemical classification analysis shows that the compounds are predominantly composed of intact tirucallane-type (86.1%). Smaller proportions of structurally modified derivatives were also identified, including *seco* (4.7%), heteroatom-containing (3.7%), degraded (2.9%), and highly rearranged tirucallane (2.6%) compounds. Further differentiation based on side-chain architecture showed that acyclic (38.7%) and cyclic side chains with epoxide groups (21.5%) were the dominant features, followed by cyclic 5-carbon (16.5%), cyclic 6-carbon (7.9%), cyclic 4-carbon (3.2%), cyclic 7-carbon (2.2%), and glycoside side chains (1.3%). From a bioactivity perspective, the compounds show a strong predominance of cytotoxic activity (54.4%), underscoring the potential relevance in anticancer studies. This is followed by anti-inflammatory (18.9%) and antimicrobial (11.1%) activities, with the remaining 15.6% of compounds showing other biological activities, dominated by α-glucosidase inhibitory (39.3%) and agonistic activity (35.7%), alongside antiplasmodial (14.3%) and antifeedant (10.7%) effects. Several compounds demonstrated significant activity, indicating potential as lead molecules for anticancer, anti-infective, and metabolic disorder therapies. Future studies should focus on mechanistic clarification, SAR analysis, advanced biosynthetic investigations, and comprehensive in vivo evaluations to improve pharmacological relevance. These results show that tirucallane-type triterpenoids from the Meliaceae family serve as a valuable natural source for future drug discovery and development.

## Data Availability

No data was used for the studies described in the article.

## Abbreviations

DCM: Dichloromethane
PE: Petroleum ether
MeOH: Methanol
EtOAc: Ethyl acetate
EtOH: Ethanol
ACHN: Human kidney cancer cells
MIC: Minimum Inhibitory Concentration
MCF-7: Human breast cancer cells
AGZY 83-a: Human lung cancer cells
SMMC-7721: Human liver cancer cells
TRAIL: Tumor necrosis factor-related apoptosis-inducing ligand
AGS: Human gastric adenocarcinoma cells
HL-60: Human leukemia cells
Bel7402: Hepatocellular carcinoma cells
HepG2: Human liver cancer cells
HeLa: Human cervical cancer cells
SW480: Human colon cancer cells
A549: Human lung cancer cells
K-562: Human erythroleukemic cells
NCI-H522: Lung tumor
P-388: Mouse lymphocytic leukemia
HT-29: Human colon adenocarcinoma
NF-kB: Nuclear factor kappa-light-chain-enhancer of activated B cells
TNF-α: Tumor necrosis factor alpha
IL-6: Interleukin-6
MRSA: Methicillin-resistant Staphylococcus aureus
Nrf2/HO-1: Nuclear factor erythropoietin-2-related factor 2/heme oxygenase 1
PTP1B: Human protein tyrosine phosphatase 1B
IC_50_: Half maximal inhibitory concentration
EC_50_: Half maximal effective concentration
ED_50_: The dose that produces a biological response in 50% of the population
G1_50_: 50% Of maximal inhibition of cell proliferation
Glucose-6P: Glucose-6 phosphate
